# Systematic reconstruction of cellular trajectories across mouse embryogenesis

**DOI:** 10.1038/s41588-022-01018-x

**Published:** 2022-03-14

**Authors:** Chengxiang Qiu, Junyue Cao, Beth K. Martin, Tony Li, Ian C. Welsh, Sanjay Srivatsan, Xingfan Huang, Diego Calderon, William Stafford Noble, Christine M. Disteche, Stephen A. Murray, Malte Spielmann, Cecilia B. Moens, Cole Trapnell, Jay Shendure

**Affiliations:** 1 Department of Genome Sciences, University of Washington, Seattle, WA USA; 2 The Rockefeller University, New York, NY USA; 3 The Jackson Laboratory, Bar Harbor, ME USA; 4 Medical Scientist Training Program, University of Washington, Seattle, WA USA; 5 Paul G. Allen School of Computer Science & Engineering, University of Washington, Seattle, WA USA; 6 Department of Pathology, University of Washington, Seattle, WA USA; 7 Department of Medicine, University of Washington, Seattle, WA USA; 8 Human Molecular Genomics Group, Max Planck Institute for Molecular Genetics, Berlin, Germany; 9 Institute of Human Genetics, University of Lübeck, Lübeck, Germany; 10 Division of Basic Sciences, Fred Hutchinson Cancer Research Center, Seattle, WA USA; 11 Brotman Baty Institute for Precision Medicine, Seattle, WA USA; 12 Allen Discovery Center for Cell Lineage Tracing, Seattle, WA USA; 13 Howard Hughes Medical Institute, Seattle, WA USA

**Keywords:** Embryogenesis, Computational biology and bioinformatics, Gene expression

## Abstract

Mammalian embryogenesis is characterized by rapid cellular proliferation and diversification. Within a few weeks, a single-cell zygote gives rise to millions of cells expressing a panoply of molecular programs. Although intensively studied, a comprehensive delineation of the major cellular trajectories that comprise mammalian development in vivo remains elusive. Here, we set out to integrate several single-cell RNA-sequencing (scRNA-seq) datasets that collectively span mouse gastrulation and organogenesis, supplemented with new profiling of ~150,000 nuclei from approximately embryonic day 8.5 (E8.5) embryos staged in one-somite increments. Overall, we define cell states at each of 19 successive stages spanning E3.5 to E13.5 and heuristically connect them to their pseudoancestors and pseudodescendants. Although constructed through automated procedures, the resulting directed acyclic graph (TOME (trajectories of mammalian embryogenesis)) is largely consistent with our contemporary understanding of mammalian development. We leverage TOME to systematically nominate transcription factors (TFs) as candidate regulators of each cell type’s specification, as well as ‘cell-type homologs’ across vertebrate evolution.

## Abstract (web summary)

Integrative analysis of single-cell RNA-sequencing datasets across mouse gastrulation and organogenesis identifies cell states and trajectories at successive developmental stages, along with transcription factors that could potentially mediate lineage choices.

## Main

A fundamental goal of developmental biology is to understand the lineage relationships of cells and cell types to one another, as well as the molecular programs that underlie each cell type’s emergence. In principle, developmental programs can be comprehensively described, as in Sulston and colleagues’ heroic reconstruction of the complete embryonic lineage of the roundworm *Caenorhabditis elegans*^1^. However, *C. elegans—*small, translucent, and developmentally invariant*—*remains the only model organism for which such a complete description has been realized.

Since 2016, we and others have developed and applied new technologies for single-cell molecular profiling at the ‘whole-animal’ scale, including worm, fly, zebrafish, frog and mouse^2–7^. Such studies lay the foundations for global views of animal development, such as by populating the Sulston lineage of *C. elegans* with the gene expression programs of each cell type^7,8^.

For mouse, the whole embryo has been profiled by scRNA-seq during implantation^9,10^, gastrulation^2^ and organogenesis^4^. Collectively, these studies span development from dozens of cells of a few types (E3.5) to millions of cells of hundreds of types (E13.5). However, the associated data have yet to be systematically integrated in a manner that permits their robust exploration. Such integration is challenging, both for technical reasons (e.g., different technologies and batch effects) and because of the sheer complexity of mouse development.

Here, we set out to systematically reconstruct the major cellular trajectories of mammalian embryogenesis from E3.5 to E13.5. Our primary strategy, inspired by Briggs and colleagues^5^, makes several assumptions: (1) although mouse development is variable, key patterns will be consistent across animals; (2) *omnis cellula e cellula* also applies to cell types (i.e., cell types observed at a given time point must have arisen from cell types present at the preceding time point); (3) we are sampling frequently and deeply enough that newly detected cell types will not arise from antecedent cell types undetected at the preceding time point; and (4) assuming sampling time points are closely spaced, transcriptional similarity is an effective means of linking related cell types across time.

A caution is that in contrast to the Sulston et al.’s seminal map of *C. elegans*, we focus here on reconstructing trajectories^11^, a concept related, but by no means equivalent, to lineage. Although it is a reasonable expectation that closely related cells (e.g., siblings) will be transcriptionally similar^8^, the converse is not necessarily true. For example, lineally distant cells might be insufficiently divergent, or even convergent, obscuring lineage relationships^12^. Furthermore, even the expectation that closely related cells will be transcriptionally similar is not always met, as rapid changes can lead to ‘gaps’ in trajectories^8^. In sum, our goal here is a continuous, navigable roadmap of the transcriptional states of cell types during mouse development. Such a roadmap may constrain the potential lineage relationships among constituent cell types, but it does not explicitly specify them.

## Results

### Intensive scRNA-seq of individual, somite-resolved embryos

**Fig. 1:**
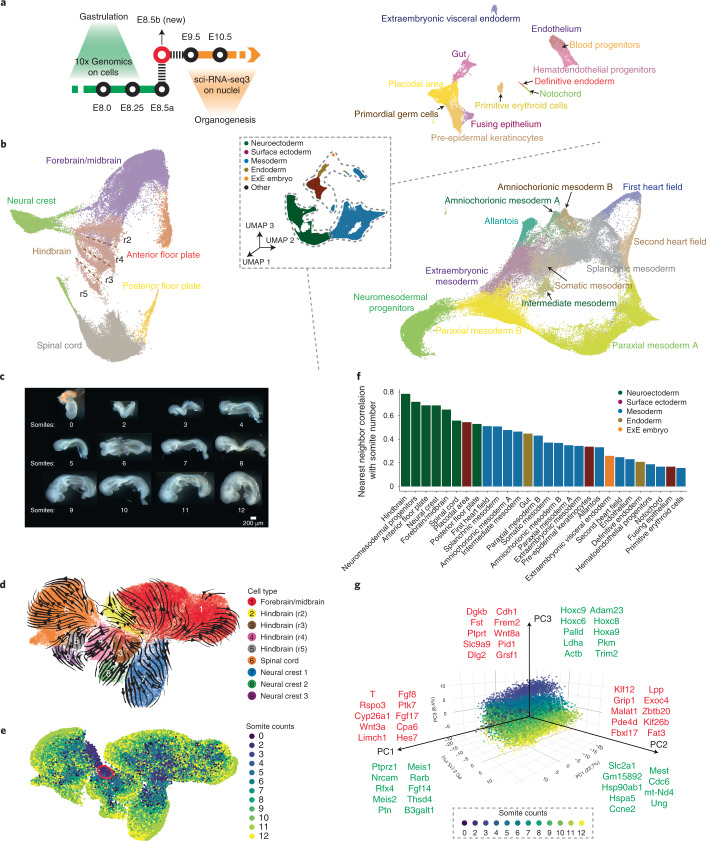
Intensive scRNA-seq of somite-resolved E8.5 mouse embryos. **a**, A new scRNA-seq dataset was generated from nuclei derived from individual E8.5 mouse embryos via an optimized sci-RNA-seq3 protocol to bridge existing data generated on E8.5 cells via 10x Genomics^2^ and E9.5 nuclei via sci-RNA-seq3 (ref. ^4^). **b**, 3D UMAP visualizations of the new E8.5 dataset (E8.5b). All nuclei colored by germ layer are shown in the center, along with separate embeddings of neuroectoderm (left), nonhematopoietic mesoderm (bottom right) and endoderm, extraembryonic and hematopoietic cell types (top right). **c**, Twelve mouse embryos, including a single primitive-streak-stage embryo and 11 embryos staged in 1-somite increments from 2 to 12 somites, were collected and their nuclei subjected to optimized sci-RNA-seq3. **d**, Re-embedded two-dimensional (2D) UMAP of cells annotated as forebrain, midbrain, hindbrain, spinal cord and neural crest. Arrows correspond to RNA velocity trends^97^. **e**, The same UMAP as in **d**, colored by somite counts. The subset of cells from rhombomere 4 that appear to emerge the earliest are highlighted in red circles (*Hoxa1*^+^ and *Hoxb1*^+^)^23,24^. **f**, For each cell type with >100 profiled cells, we calculated the Pearson correlation coefficient between the somite number of each cell of that type and the average somite number of its five nearest neighbors in the global 3D UMAP embedding. Colors indicate germ layers. **g**, 3D visualization of the top three PCs of gene expression variation in NMPs, calculated on the basis of the 2,500 most highly variable genes. Cells are colored by the somite count of the originating embryo. Genes most strongly correlated (Pearson), either positively (red) or negatively (green), with each PC are listed. ExE, extraembryonic; r2–r5: rhombomeres 2–5.

**Extended Data Fig. 1:**
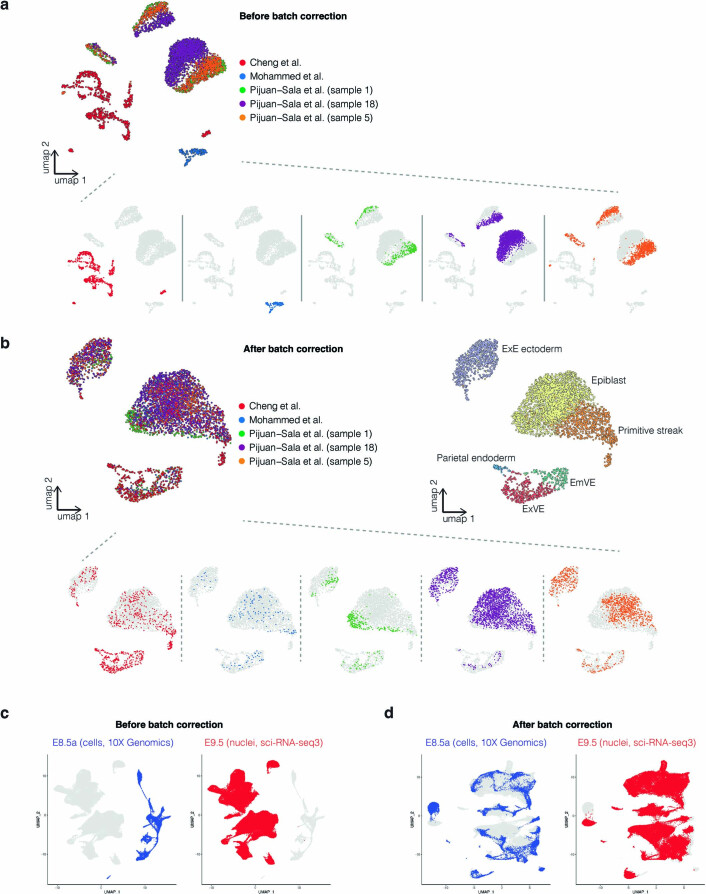
Integration of datasets generated by different groups using different scRNA-seq technologies. **a**, As illustrated by a UMAP of coembedded E6.5 cells, batch effects are observed between three studies, as well as different embryos from the same study. The same UMAP is shown several times on the bottom of the panel, with colors highlighting cells from different studies or samples. **b**, UMAP of the same cells as in panel a with batch correction prior to integration^13^. The same UMAP is shown several times on the bottom of the panel, with colors highlighting cells from different studies or samples. In addition, the same UMAP is shown on the upper right, but colored by cell-type annotation. **c**, UMAP visualization of co-embedding of data from E8.5a (cells) generated on the 10x Genomics platform^2^ and E9.5 (nuclei) generated using sci-RNA-seq3^4^, before batch correction^13^. The same UMAP is shown twice for both, with colors highlighting cells from either E8.5a (left) or E9.5 (right). E9.5 profiles were based on deeper sequencing of the same libraries reported in Cao *et al*.^4^. **d**, UMAP of the same cells as in panel c but with batch correction prior to integration^13^. Left and right as in panel c. ExE: extraembryonic. EmVE: embryonic visceral endoderm. ExVE: extraembryonic visceral endoderm.

**Extended Data Fig. 2:**
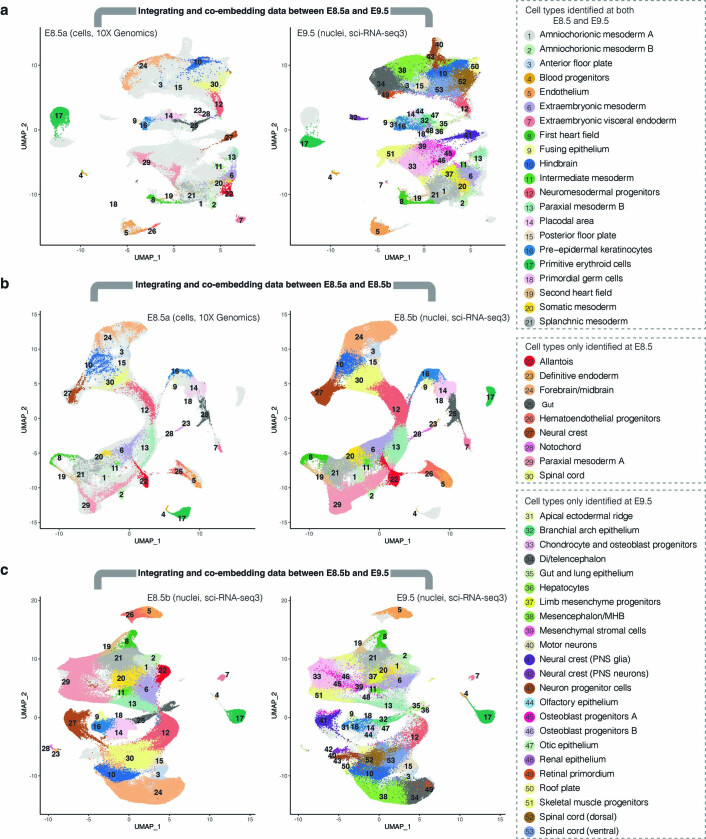
Integrating and coembedding cells from E8.5a, E8.5b and E9.5. For panels a and c, E9.5 profiles were based on deeper sequencing of the same libraries reported in Cao *et al*.^4^. **a**, UMAP visualization of coembedded cells at E8.5a generated on the 10x Genomics platform^2^ and nuclei at E9.5 generated using sci-RNA-seq3^4^ after batch correction^13^. The same UMAP is shown twice for both, with colors highlighting cells/nuclei from either E8.5a (left) or E9.5 (right). **b**, UMAP visualization of coembedded cells at E8.5a generated on the 10x Genomics platform^2^ and nuclei at E8.5b generated using sci-RNA-seq3^4^ after batch correction^13^. The same UMAP is shown twice for both, with colors highlighting cells/nuclei from either E8.5a (left) or E8.5b (right). **c**, UMAP visualization coembedded nuclei at E8.5b and nuclei at E9.5, both generated with sci-RNA-seq3^4^, after batch correction^13^. The same UMAP is shown twice for both, with colors highlighting nuclei from either E8.5b (left) or E9.5 (right).

The datasets that we sought to integrate were generated by different groups at different times with different technologies (Supplementary Table 1). To address this, we performed anchor-based batch correction^13^ prior to integration, which proved quite effective, including across technologies (Extended Data Fig. 1). However, the integration of E8.5 (cells, 10x Genomics) and E9.5 (nuclei, three-level single-cell combinatorial-indexing RNA-sequencing (sci-RNA-seq3)) data was particularly challenging. Numerous cell types appeared or disappeared between these time points^2,4^, and it was unclear which changes were due to technical differences versus bona fide developmental progression (Extended Data Fig. 2a). To address this, we set out to generate new data at E8.5 that might serve as a ‘Rosetta Stone’ of sorts (Fig. 1a,b).

Because of how quickly changes are occurring around this time point, we focused on individual, somite-resolved embryos. We selected 12 embryos from 2 litters harvested at E8.5, including a single primitive-streak-stage embryo (prior to somitogenesis) and 11 embryos staged in 1-somite increments from 2 to 12 (Fig. 1c). A simplified, optimized version^14^ of sci-RNA-seq3 markedly improved data quality relative to the original protocol^4^ (Methods, Supplementary Note 1 and Supplementary Fig. 1a). After quality filtering, we obtained profiles for 154,313 somite-staged E8.5 nuclei (median unique molecular identifier (UMI) count, 7,672; median genes detected, 3,463) (Supplementary Fig. 1b,c).

Batch correction and integration of published E8.5 data (cells, 10x Genomics; termed E8.5a) with these new data (nuclei, sci-RNA-seq3, termed E8.5b) worked very well except for primitive erythroid cells, possibly due to more extensive differences between cells versus nuclei in this cell type (Extended Data Fig. 2b). As expected, because they were generated on nuclei with the same technology, integration of E8.5b and E9.5 profiles also worked well (Extended Data Fig. 2c).

**Extended Data Fig. 3:**
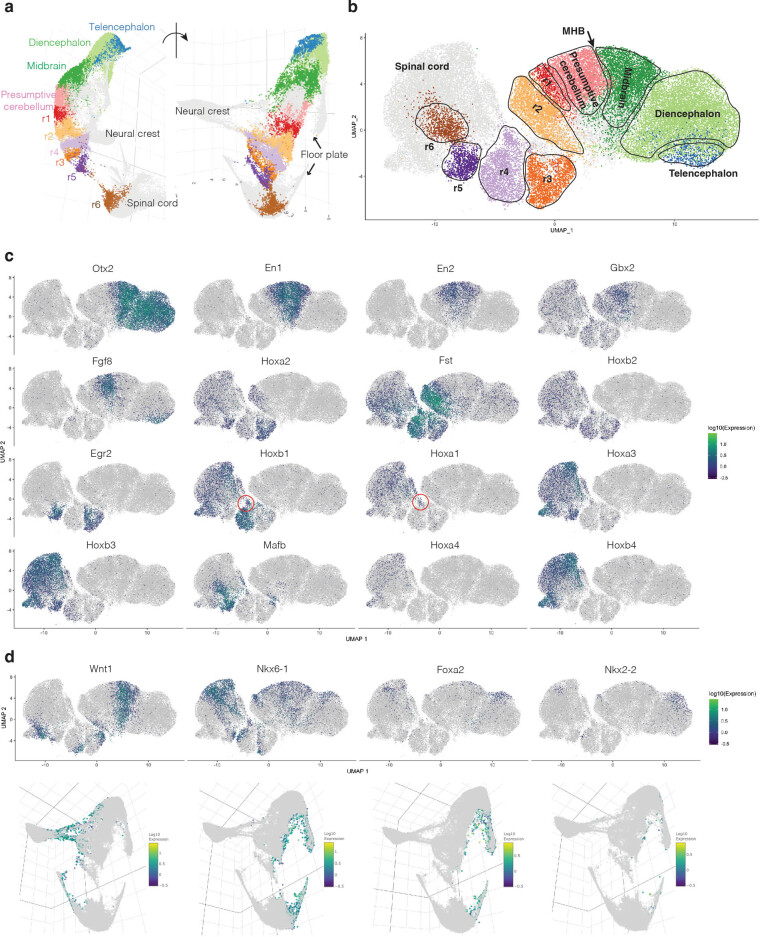
Resolution of hindbrain segmentation in newly created E8.5 dataset. **a**, Subview of global 3D UMAP visualization highlighting subsets of cells annotated as rhombomeres 1 - 6 (r1 - 6) in E8.5 data generated with optimized sci-RNA-seq3 protocol. **b**, Re-embedded 2D UMAP of cells annotated as forebrain, midbrain, presumptive cerebellum, r1–r6, spinal cord and neural crest, although neural crest cells are excluded from visualization. **c**, The same UMAP as in panel b, colored by gene expression of marker genes used for annotation of anatomical regions. Telencephalon: *Otx2*+, *Fgf8* + ; Diencephalon: *Otx2*+, *En1*-, *En2*-; Midbrain: *Otx2*+, *En1*+, *En2*+, *Fgf8*-; MHB (midbrain–hindbrain boundary): boundary of *Fgf8* and *Wnt1*; Presumptive cerebellum: *Fgf8*+, *En1*+, *En2*+, *Wnt1*-, *Gbx2* + ; r1: a ‘wedge’ between cerebellum and r2, *Fgf8*-, *Hoxa2*-; r2: *Fst*+, *Hoxa2*+, *Hoxb2*-; r3: *Egr2*+, *Hoxb2*+, *Hoxa3*-, *Hoxb3*-; r4: *Fst*+, *Hoxa1*+, *Hoxb1*+, *Hoxa3*-, *Hoxb3*-; r5: *Egr2*+, *Hoxa3*+, *Hoxb3*+, *Mafb* + ; r6: *Mafb*+, *Egr2*-, *Hoxb4*-^21,22,112–115^. The subset of cells from r4 which appear to emerge earlier than the other rhombomeres cells are highlighted by red circles in the third row (*Hoxa1*+, *Hoxb1*+)^23,24^. **d**, The same UMAP as in panel b, colored by gene expression of marker genes for the dorsal-ventral axis (*Wnt1* is a dorsal marker; *Nkx6-1*, *Foxa2* and *Nkx2-2* are ventral markers)^25,26^. The same genes are highlighted in the 3D subview of panel a are shown below. Gene expression values shown in panel c-d were calculated by normalizing the UMI counts by the estimated size factors followed by log10-transformation.

The E8.5b data enabled identification of the same 30 cell types as found in E8.5a data^2^ (Fig. 1b, Extended Data Fig. 2 and Supplementary Table 2). However, the depth of the new data, together with additional temporal resolution afforded by somite staging of individual embryos, facilitated the identification of substantial substructure. Examples include:

1. Floor plate: We observe two, clearly distinct subpopulations that express the floor plate markers *Foxa2* and *Shh* (Fig. 1b and Supplementary Fig. 2) (ref. ^15^). Although these appear to be converging toward a common transcriptional state, an anterior subpopulation (*Bmp7*^+^) arises from the forebrain/midbrain, whereas a posterior subpopulation arises from the spinal cord^16^.
2. Heart fields: We observe subpopulations arising from the splanchnic mesoderm that correspond to the first (*Tbx5*^+^ and *Hcn4*^+^) and second (*Isl1*^+^ and *Tbx1*^+^) heart fields (Fig. 1b and Supplementary Fig. 3) (refs. ^17–20^). Similar to the floor plate, although these appear to converge toward a common transcriptional state, the heart fields remain distinguished by these and other markers throughout early somitogenesis.
3. Rhombomeres: We observe four subpopulations of hindbrain, and two subpopulations within midbrain and spinal cord, that appear to correspond to rhombomeres 1–6 (Fig. 1b and Extended Data Fig. 3). These annotations are based on distinct combinations of Hox markers and other genes. For example, rhombomeres 3 and 5 specifically express *Egr2*, whereas rhombomere 5 further expresses *Hoxa3*, *Hoxb3* and *Mafb*^21,22^. Each rhombomere includes cells from embryos spanning somitogenesis, consistent with roughly concurrent, rather than sequential, differentiation. However, a subset of cells from rhombomere 4 are from the earliest embryos of the series and express *Hoxa1* and *Hoxb1*, consistent with the possibility that rhombomere 4 begins to develop first (Fig. 1d,e) (refs. ^23,24^). Although we must be cautious about interpreting uniform manifold approximation and projection (UMAP) topologies, the rhombomeres are ordered along a rostral–caudal axis in relation to other major aspects of neuroectoderm regionalization, with *Wnt1* and *Nkx6-1* expression further marking dorsal and ventral regions, respectively (Extended Data Fig. 3) (refs. ^25,26^).
4. Neural crest: In the global embedding, we observe three distinct subpopulations of neural crest cells (NCCs) that appear to derive from different subsets of neuroectoderm (Fig. 1b). Reanalysis with RNA velocity and examination of *Hox* gene expression suggests that these three populations may correspond to mesencephalic and pharyngeal arch 1 (PA1) NCCs, PA2 NCCs and PA3 NCCs (Fig. 1d and Supplementary Fig. 4). Differential patterns of early neural crest marker expression (e.g., *Foxd3*), as well as their distribution in relation to somitogenesis, are consistent with these subpopulations emerging asynchronously (Fig. 1e and Supplementary Fig. 4) (ref. ^27^).

**Extended Data Fig. 4:**
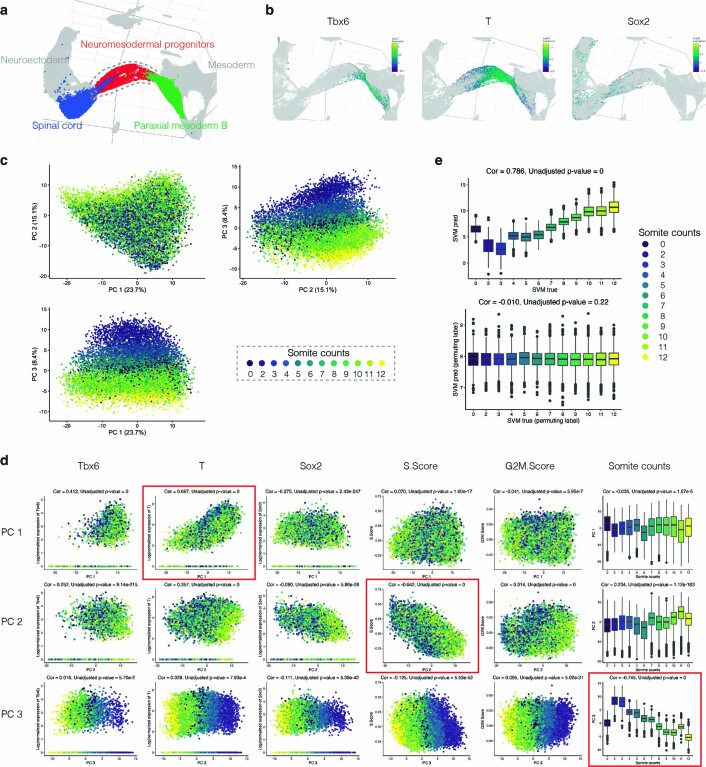
Decoding of transcriptional heterogeneity within NMPs. **a**, Subview of global 3D UMAP visualization highlighting spinal cord (blue), neuromesodermal progenitors (red), and paraxial mesoderm B (green). **b**, The same 3D UMAP as panel a but zooming in to highlight NMP cells, colored according to expression levels of markers of mesodermal (*Tbx6*, *T*) or neuroectodermal (*Sox2*) state^28,29^. Gene expression values were calculated by normalizing the UMI counts by the estimated size factors followed by log10-transformation. **c**, Embeddings of NMP cells (*n* = 14,869 cells) in PCA space with visualization of top three PCs, calculated on the basis of the 2,500 most highly variable genes, in 2D. Cells are colored by the somite count of the originating embryo. **d**, Correlations between top three PCs (rows 1-3) and the normalized expression of selected genes (*Tbx, T, Sox2*; columns 1-3), cell cycle indices (columns 4-5) or somite counts (column 6) (*n* = 14,869 cells). Red boxes highlight the strongest absolute correlation in each row. Coefficients and unadjusted p-values were calculated by two-sided Pearson correlation and are shown above the plots. Gene expression values were calculated from original UMI counts normalized to total UMIs per cell, followed by natural-log transformation. Cell cycle indices were estimated using the *CellCycleScoring* function of *Seurat*/v3 (S.Score and G2M.Score). **e**, The 114 genes most strongly correlated with PC3 (which appears to correlate to somite counts) were identified using two-sided Pearson correlation (out of the 5,000 most variable genes; FDR < 0.05 and absolute coefficients > 0.2; Supplementary Table 3). The *sklearn.svm.LinearSVR* function in scikit-learn/1.0 was applied to assess whether the somite counts of the originating embryos of NMP cells could be predicted from their transcriptional profiles. The distributions of true (*x*-axis) vs. predicted (*y*-axis) somite counts for NMP cells are shown, without (top) or with (bottom) permutation of somite count labels (*n* = 14,869 cells). Coefficients and unadjusted p-values were calculated by two-sided Pearson correlation and are shown above the plots. In the boxplots shown in panel d and e, the center lines show the medians; the box limits indicate the 25th and 75th percentiles; the whiskers extend to the 5th and 95th percentiles; the outliers are represented by the dots.

We next sought to systematically explore the extent to which the transcriptional dynamics of individual cell types are coordinated with the timing of somite formation. For each cell type, we calculated the correlation between cell somite counts and those of their five nearest neighbors in a global three-dimensional (3D) UMAP embedding. In this framing, high correlations are consistent with rapid, ‘within-cell-type’ changes in transcriptional state that are synchronized with somite counts. Consistent with our earlier analyses (Fig. 1e and Supplementary Fig. 2c), the highest such correlations were for neuroectodermal cell types, rather than the somites themselves (Fig. 1f). Focusing on neuromesodermal progenitors (NMPs), whose heterogeneous states bridge paraxial mesoderm and spinal cord neuroectoderm, the top principal components (PCs) of transcriptional variation are strongly correlated with mesodermal (*T* (Brachyury)^*+*^ and *Tbx6*^+^) versus neuroectodermal (*Sox2*^+^) state (PC1; 23.7% of variation), cell cycle index (PC2; 15.1% of variation) and somite count (PC3; 8.4% of variation) (Extended Data Fig. 4 and Supplementary Table 3) (refs. ^28,29^). The genes most highly correlated with these PCs are shown in Fig. 1g. For example, key regulators of mesoderm (*T*) (ref. ^30^), the somite segmentation clock (*Hes7*) (ref. ^31^) and Wnt signaling (*Wnt3a*, *Rspo3* and *Ptk7*) (ref. ^32,33^) are positively correlated with PC1, whereas regulators or effectors of neural adhesion or neurite outgrown (*Ptprz1*, *Nrcam* and *Ptn*)^34–36^, as well as retinoic acid signaling (*Rarb*), are negatively correlated.

### Reconstruction of trajectories spanning mouse embryogenesis

**Extended Data Fig. 5:**
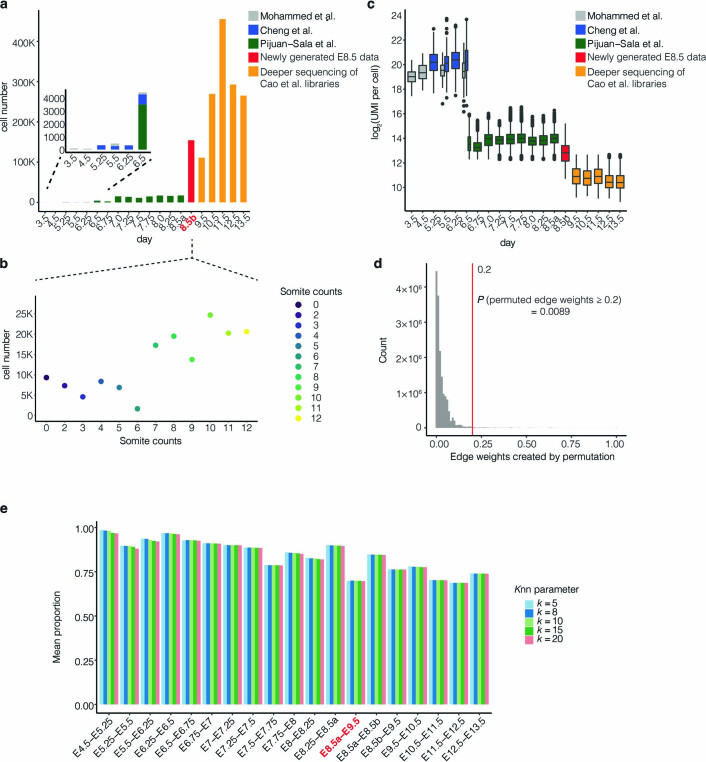
Integration of datasets spanning E3.5 to E13.5 of mouse development. **a**, The number of cells per stage obtained from three previous studies^2,9,10^, new E8.5 data obtained via optimized sci-RNA-seq3, and deeper sequencing of Cao *et al*.^4^. **b**, The number of cells per embryo corresponding to specific somite counts from new E8.5 data. **c**, Box plot of log2(UMI counts) per cell across the stages and studies (*n* = 1,658,968 cells).The center lines show the medians; the box limits indicate the 25th and 75th percentiles; the whiskers extend to the 5th and 95th percentiles; the outliers are represented by the dots. **d**, The same strategy of creating the edges between adjacent time points was performed after randomly shuffling the cell-state annotations for cells within each time point, followed by repeating this process 1,000 times, resulting in a null distribution of edge weights. After permutation, less than 1% of potential edges are assigned weights greater than 0.2 (red line). **e**, To quantify the quality of the integration between adjacent time points, we focused on cells at the later time point assigned to annotations that were also present at the earlier time point. We then calculated the fraction of these cells’ ancestral *k*-nearest neighbors (in the global 3D UMAP co-embedding) that were assigned the identical annotation. The mean proportion for different values of *k* are reported in the histogram. Of note, the lower value of this metric for E8.5a-E9.5 (red label) than E8.5a-E8.5b or E8.5b-E9.5 provides quantitative support for our claim that the new E8.5b data improved integration across the E8.5 to E9.5 (Extended Data Fig. 2).

We collated data from three studies spanning E3.5 to E8.5 (refs. ^2,9,10^), the new E8.5 data described above (Fig. 1a,b) and data from one study spanning E9.5 to E13.5 but with deeper sequencing of those libraries (Supplementary Fig. 1) (ref. ^4^). Altogether, these data derive from 480 samples (individual or small pools of embryos) from 19 stages spanning E3.5 to E13.5 (successive stages separated by 6 hours to 1 day) and include 1,658,968 cells or nuclei (67 to 455,124 per stage) (Supplementary Table 1 and Extended Data Fig. 5a–c). For each stage, we performed preprocessing, Louvain clustering and manual cluster annotation (Supplementary Figs. 5 and 6 and Supplementary Table 2). Here we use ‘cell state’ to mean an annotated cluster at a given stage. Altogether, we identified 473 cell states across the 19 stages, each of which received one of 94 cell-type annotations.

For each pair of adjacent stages, we performed anchor-based batch correction followed by projection into a shared embedding space^13^. We then applied a *k*-nearest-neighbor (*k*-NN) heuristic to connect cell states between adjacent stages (Supplementary Note 2). Because these are inferred relationships based on transcriptional similarity, analogous to pseudotime, we use ‘pseudoancestor’ and ‘pseudodescendant’ to refer to relationships between cell states across time.

**Fig. 2:**
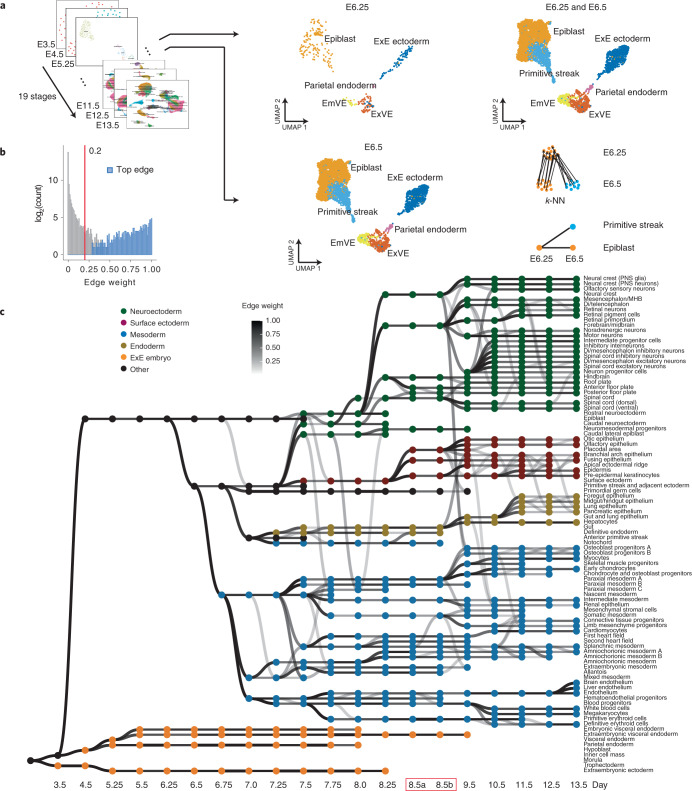
Systematic reconstruction of the cellular trajectories of mouse embryogenesis. **a**, Overview of approach. Cells from each pair of adjacent stages were projected into the same embedding space^13^. UMAP visualizations of coembedded cells from E6.25 and E6.5 are shown separately (middle column) or together (top right). A *k*-NN heuristic was applied to infer one or several pseudoancestors for each of the cell states observed at the later time point (bottom right). **b**, Histogram of all calculated edge weights. The *y* axis is on a log_2_ scale. Edges with weights above 0.2 (red line) were retained. Top edges are those with the highest weight amongst all potential antecedents of each cell state. **c**, Directed acyclic graph showing inferred relationships between cell states across early mouse development. Each row corresponds to one of 94 cell-type annotations, columns to developmental stages spanning E3.5 to E13.5, nodes to cell states and node colors to germ layers. All edges with weights above 0.2 are shown in grayscale. Of note, placental tissues were not actively retained during the isolation of embryos from later time points^4^. E8.5a and E8.5b were essentially treated as two distinct time points, because they are bridging datasets that are substantially different from a technical perspective (Fig. 1a and Extended Data Fig. 2). Di, diencephalon; EmVE, embryonic visceral endoderm; ExE, extraembryonic; ExVE, extraembryonic visceral endoderm; MHB, midbrain–hindbrain boundary; PNS, peripheral nervous system.

For example, clustering and annotation of data from two adjacent time points, E6.25 and E6.5, identified five and six cell states, respectively (Fig. 2a). Coembedding these data and following the aforedescribed procedure, we linked five states at E6.5 to five identically annotated states at E6.25. The new state at E6.5, annotated as primitive streak, was linked to E6.25 epiblast, which we assigned as its pseudoancestor (Fig. 2a). Applying this procedure to E6.5→E6.75 and E6.75→E7.0, the primitive streak was further assigned as the pseudoancestor of nascent mesoderm, anterior primitive streak and primordial germ cells (Supplementary Fig. 7).

We applied this approach to each pair of adjacent stages (Supplementary Figs. 8 and 9; E8.5a and E8.5b were treated as distinct, adjacent stages). Although the resultant edge weights were bimodally distributed, a cutoff of 0.2 was selected to be more inclusive of weaker relationships and ensure connectivity of the overall graph (Fig. 2b, Extended Data Fig. 5d,e and Supplementary Note 3). The resulting representation is a directed acyclic graph with 477 nodes and 577 edges that captures TOME (Fig. 2c).

### Do molecular trajectories recapitulate cellular phylogenies?

To reiterate, TOME does not reflect cell lineage but rather cell-state relationships inferred on the basis of transcriptional similarity. Nonetheless, under the supposition that lineally related cell types diverge from one another through a succession of continuous molecular states, we can ask whether or not established lineage relationships are recapitulated by TOME. In Supplementary Table 4, we show all edge weights and comment on inferred transitions. Several observations merit emphasis.

First, the graph largely respects germ layers (Fig. 2c). There are no edges between extraembryonic and embryonic cell states and few edges between embryonic cell states of different germ layers. Among the strongest edges crossing germ layers are E8.5–E9.5 edges connecting neural crest to osteoblast progenitors subtypes^37^ and an E7.5–E8.0 edge between caudal lateral epiblast and a paraxial mesoderm subtype^38^. Although these examples are supported by the literature, we also observe edges between epithelia derived from different germ layers that are probably consequent to transcriptional convergence rather than shared lineage^4,39^.

Second, 80% of cell types are strongly linked to a single pseudoancestor when they first appear (edge weight >0.7). These strong edges generally respect established lineage relationships, such as parietal and visceral endoderm arising from hypoblast^40^, notochord and definitive endoderm arising from the anterior primitive streak^41,42^, the first and second heart fields successively arising from splanchnic mesoderm^43^ and many others.

Third, apparent convergences (instances wherein we assign more than one pseudoancestor to a cell state) sometimes correspond to a given cell type persisting and ‘contributing’ to another cell type over several consecutive time points (e.g., hemoendothelial progenitors→endothelial cells). In other cases, apparent convergences may reflect incomplete separation between highly related cell types rather than ongoing differentiation (e.g., recurring edges between mesodermal subtypes). However, other cases may reflect instances where a cell type truly has multiple origins (e.g., neural crest and paraxial mesoderm A→osteoblast progenitors A and B^37^; nascent mesoderm and caudal lateral epiblast→paraxial mesoderm C (ref. ^38^)). Of note, not all ‘multiple origin’ instances are captured; for example, the established contribution of embryonic visceral endoderm to the gut^44^ is detected at E7.5–E7.75 but falls short of the edge weight threshold (Supplementary Table 4).

**Fig. 3:**
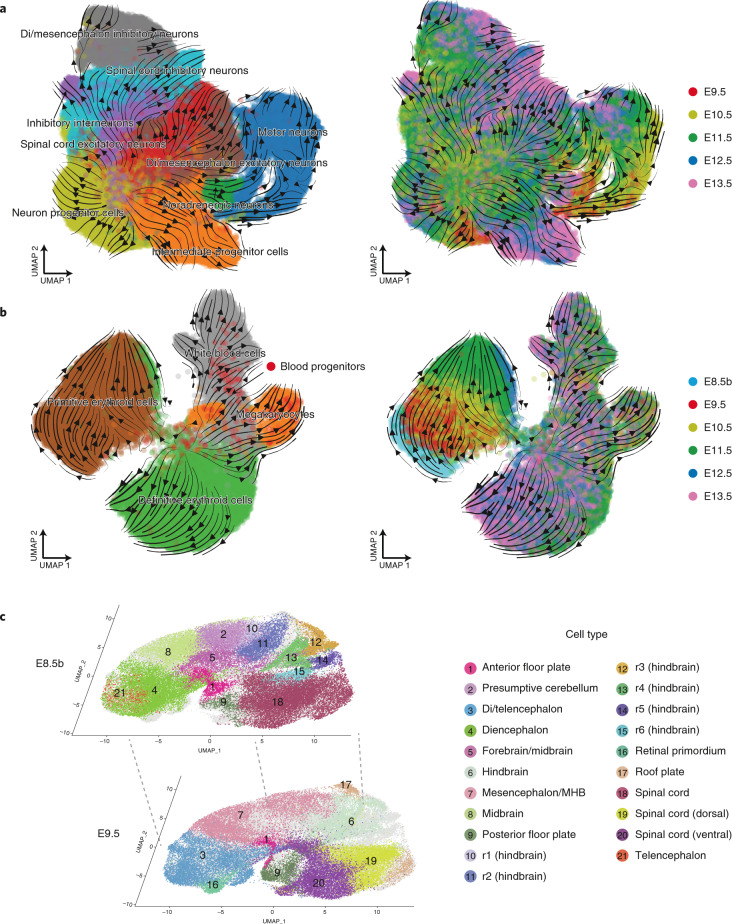
RNA velocity and spatially correlated coembeddings clarify relationships between cell types during neuronal differentiation, hematopoiesis and neural tube development. **a**, RNA velocity was estimated on the basis of the proportion of reads mapping to exonic versus intronic portions of genes using scVelo (ref. ^97^). Cells corresponding to motor neurons, noradrenergic neurons, di/mesencephalon inhibitory neurons, spinal cord inhibitory neurons, di/mesencephalon excitatory neurons, spinal cord excitatory neurons, inhibitory interneurons, intermediate progenitor cells and neuron progenitor cells from E9.5 to E13.5 were included in this analysis, after downsampling each cell state to 5,000 cells. UMAP visualization of coembedded cells and cell-state transition trends (arrows) are shown. Smaller panels show the same UMAP visualization but with coloring of cells from individual time points. **b**, Same as **a**, but for cells corresponding to blood progenitors, white blood cells, megakaryocytes, definitive erythroid cells and primitive erythroid cells from E8.5b to E13.5. **c**, UMAP visualization of coembedded cells from neural tube derivatives from E8.5b and E9.5 data after batch correction^13^. The same UMAP is shown twice for both, with colors highlighting cells and corresponding annotations from either E8.5b (top) or E9.5 (bottom).

**Extended Data Fig. 6:**
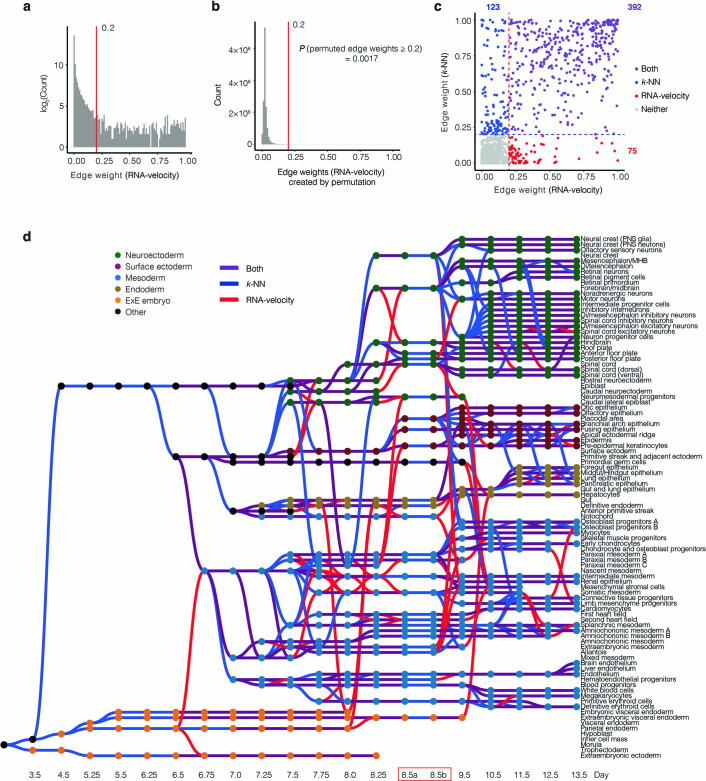
TOME edges nominated by *k*-NN versus RNA velocity-based heuristics are largely concordant. **a**, Histogram of all potential edge weights calculated by RNA velocity. The *y*-axis is on a log2 scale. Edges with weights above 0.2 (red line) were retained. **b**, After calculating the transition probability for individual cells between adjacent time points using *scVelo*^97^, the same strategy of creating the edges was performed after randomly shuffling the cell-state annotations for cells within each time point, followed by repeating this process 1,000 times, resulting in a null distribution of edge weights. After permutation, less than 1% of potential edges are assigned weights greater than 0.2 (red line). **c**, Ignoring edges prior to E6.5 as well as between E8.5a and E8.5b (see text), out of 15,261 potential edges, there were 123 edges nominated by the *k*-NN strategy only (weight > 0.2), and 75 edges nominated by the RNA velocity strategy only (weight > 0.2), and 392 nominated by both strategies. **d**, Directed acyclic graph showing inferred relationships between cell states across early mouse development. Layout identical to Fig. 2c. Each row corresponds to one of 94 cell-type annotations, columns to developmental stages spanning E3.5 to E13.5, nodes to cell states, and node colors to germ layers. Edges nominated with weights above 0.2 by RNA velocity only are shown in red, by *k*-NN in blue, and by both strategies in purple. ExE: extraembryonic. PNS: peripheral nervous system. MHB: midbrain–hindbrain boundary. Di: diencephalon.

Fourth, an important limitation of our heuristic approach, made apparent by a few clear inaccuracies in the graph, is that true lineage relationships for a given cell state can be obscured by the presence of a highly similar cell state at the preceding time point. Examples of such inaccuracies are discussed in Supplementary Note 4. Of note, at least some of these inaccuracies can be resolved through focused analyses that leverage the distinction between nascent and spliced transcripts (i.e., RNA velocity^45^) (Fig. 3a,b, Supplementary Note 4, Methods, Supplementary Figs. 10 and 11, Extended Data Fig. 6 and Supplementary Table 5).

Fifth, a further limitation is that our reliance on discrete cell states obscures aspects of development that are inherently continuous. For example, continuous spatial heterogeneity is obscured by cell-type or cell-state discretization. Nonetheless, although challenging to reduce to a graph-based representation, continuous aspects of heterogeneity, spatial or otherwise, might be retained in coembeddings across time points. For example, for neural-tube-derived cells from E8.5b and E9.5, the coembedding is potentially informative in both directions (e.g., to identify the subset of E8.5 diencephalon cells most related to E9.5 retinal primordium; or the subsets of E9.5 hindbrain cells most related to specific E8.5-annotated rhombomeres) (Fig. 3c).

In summary, molecular trajectories often recapitulate well-documented cellular phylogenies, but there are clear limitations. Nonetheless, the graph is largely consistent with our contemporary understanding of mammalian development, despite being constructed through automated procedures. To facilitate its exploration, we created an interactive website in which the nodes and edges shown in Fig. 2c can be navigated (http://tome.gs.washington.edu).

### Inference of the spatial locations of cell states

**Fig. 4:**
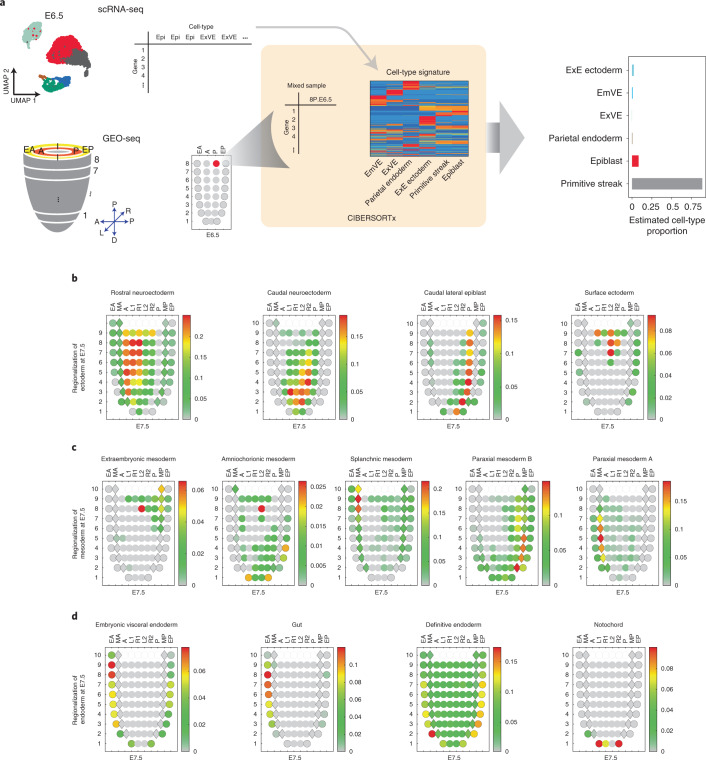
Inference of the approximate spatial locations of cell states during mouse gastrulation. **a**, Inference of cell-type contributors to each spatial territory of the gastrulating mouse embryo based on the application of CIBERSORTx to GEO-seq data^48,49^. GEO-seq yields bulk RNA-sequencing data from small numbers of cells dissected from precise anatomic regions of the gastrulating embryo^48^. We then estimated the proportional contribution of each cell state to each GEO-seq sample using CIBERSORTx (ref. ^49^). **b**, Corn plots^48^ showing the spatial pattern of inferred contributions of various ectodermal cell types at E7.5. **c**, Corn plots showing the spatial pattern of inferred contributions of various mesodermal cell types at E7.5. **d**, Corn plots showing the spatial pattern of inferred contributions of various endodermal cell types at E7.5, as well as notochord. In each corn plot, each circle or diamond refers to a GEO-seq sample and its weighted color to the estimated cell-type composition. Corn plot nomenclature from Peng et al.^48^. A, anterior; P, posterior; L, left lateral; R, right lateral; L1, anterior left lateral; R1, anterior right lateral; L2, posterior left lateral; R2, posterior right lateral; Epi1 and Epi2, divided epiblast; M, whole mesoderm; MA, anterior mesoderm; MP, posterior mesoderm; En1 and En2, divided endoderm; EA, anterior endoderm; EP, posterior endoderm.

**Extended Data Fig. 7:**
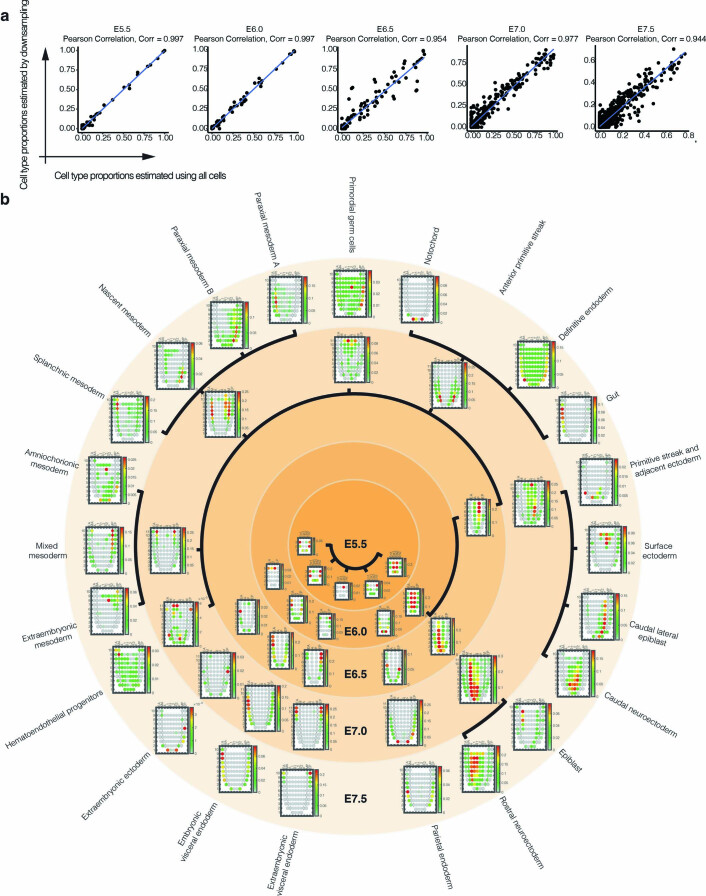
Estimated cell-type proportions for different regions of the gastrulating mouse embryo, arranged by inferred cell-type relationships over time. **a**, The inferred cell–state proportions of each GEO-seq territory are robust to downsampling. For time point which GEO-seq data was available (E5.5, E6.0, E6.5, E7.0, and E7.5), we estimated a gene expression signature for each cell state from scRNA-seq data, either by using all the cells or by downsampling to a maximum of 50 cells per state, and then repeated the inference of cell-type contributors to each spatial territory of the gastrulating mouse embryo based on the application of *CIBERSORTx* to GEO-seq data^48,49^. The Pearson correlation of resulting estimated cell–state proportions for each GEO-seq territory with downsampling (*y*-axes) or without downsampling (*x*-axes) are shown. Of note, we did not use downsampling in the results shown in Fig. 4b, As described in Fig. 4a, inference of cell-type contributor(s) to each spatial territory of the gastrulating mouse embryo based on the application of *CIBERSORTx* to GEO-seq data^48,49^. As scRNA-seq data from E6.0 was unavailable, we used data from E6.25 instead. Black edges correspond to edges between cell states over time estimated by TOME (only edges with the largest weights are shown). In each corn plot, each circle or diamond refers to a GEO-seq sample, and its weighted color to the estimated cell-type composition. Corn plot nomenclature from Peng *et al*.^48^. A, anterior; P, posterior; L, left lateral; R, right lateral; L1, anterior left lateral; R1, anterior right lateral; L2, posterior left lateral; R2, posterior right lateral; Epi1 and Epi2, divided epiblast; M, whole mesoderm; MA, anterior mesoderm; MP, posterior mesoderm; En1 and En2, divided endoderm; EA, anterior endoderm; EP, posterior endoderm.

The spatial relationships of cells are a crucial aspect of development, but this information is lost while profiling disaggregated cells or nuclei. Toward addressing this, several groups have developed in silico methods for integrating nonspatial scRNA-seq data with spatially resolved gene expression data^46,47^. For example, cryosectioning and bulk RNA-seq (geographical position sequencing (GEO-seq)) was recently applied to transcriptionally profile precise territories of the mouse embryo from E5.5 to E7.5 (ref. ^48^). Inspired by Peng et al.^48^, we leveraged TOME to estimate the abundance of individual cell types within each territory of this dataset^49^. For many cell types and territories, this approach appeared to work quite well (Fig. 4a, Extended Data Fig. 7 and Supplementary Table 6). For example, GEO-seq territories inferred to be composed of rostral and caudal neuroectoderm, caudal lateral epiblast and surface ectoderm are clearly distinguishable at E7.5 in a pattern consistent with expectation (Fig. 4b) (ref. ^50^). Also at E7.5, subtypes of paraxial mesoderm (A and B) are assignable to the anterior and posterior embryo, respectively (Fig. 4c). Finally, we observe the expected convergence of embryonic visceral endoderm and definitive endoderm cells during gut development, although the overlap is not complete^44^ (Fig. 4d and Extended Data Fig. 7b).

### Systematic nomination of key TFs for cell-type specification

Using pseudotime, embryos could be ordered by age, which in turn enabled us to calculate a smoothed expression profile for each gene along the path to each epiblast-derived cell type (Supplementary Note 5 and Supplementary Figs. 12–14). In these profiles, at least anecdotally, we observed that TFs with established roles in a given cell type were often upregulated in association with the cell type’s first appearance (Supplementary Fig. 12e).

**Fig. 5:**
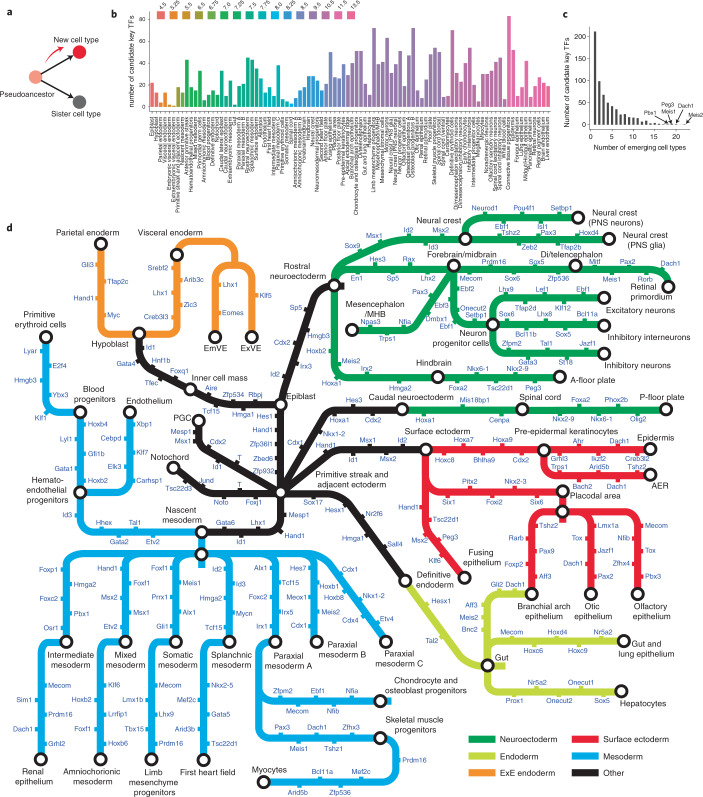
Systematic nomination of candidate key TFs for cell-type specification. **a**, We heuristically defined candidate key TFs as those that are expressed in the pseudoancestral cell state, are significantly upregulated in the newly emerged cell type and are not significantly upregulated at any sister edges. **b**, Histogram of the number of candidate key TFs for each cell type at the time point of its first emergence. **c**, The histogram of the number of cell types in which each TF was nominated as a candidate key TF. **d**, Diagram illustrating selected cellular trajectories from TOME, decorated with the top five scoring candidate key TFs for each edge. AER, apical ectodermal ridge. Style inspired by Morris et al.^98^.

Motivated by this, we sought to systematically identify TFs that are candidates for specifying each newly emerging cell type^51,52^. For each branchpoint at which a new cell type first emerged, we heuristically defined candidate key TFs as those (1) significantly upregulated in the newly emerged cell type, relative to the pseudoancestor; (2) detected in at least 10% of cells in the newly emerged cell type; and (3) not significantly upregulated at any ‘sister’ edges, relative to the newly emerged cell type (Fig. 5a). Qualifying TFs were ranked by a normalized score based on the extent of upregulation in the new cell type versus its ancestor/sister(s).

Altogether, we identified 632 candidate key TFs associated with the emergence of one or more of 92 cell types (27 ± 18 per cell type; Fig. 5b; Supplementary Table 7). 49% were specific to one or two cell types. For example, *Gsc* (goosecoid) was nominated as a key TF for the emergence of the anterior primitive streak, but no other cell type, and *Srf* solely for the first heart field^53–55^. On the other hand, a few TFs (e.g., *Meis2* and *Dach1*) were associated with the emergence of dozens of cell types (Fig. 5c). In Fig. 5d, we show the top-scoring TFs for selected trajectories. Despite our automated approach that relied on a handful of datasets, many of these TFs are established as playing critical roles in the emergence of the corresponding cell types. For example, the top three TFs identified are *Nkx2-5*, *Mef2c* and *Gata5*^56–58^ for the first heart field; *Foxj1*, *T* (Brachyury) and Noto^59–61^ for notochord; *Sox9, Msx1* and *Id2* (refs. ^62–65^) for neural crest; and *Etv2*, *Tal1* and *Gata2* (refs. ^66–68^) for hematoendothelial progenitors. In fact, when we performed a cursory literature search on the top five TFs for each cell type, we found relevant references for 494 of 533 (93%) nominations (Supplementary Table 8).

By a similar heuristic, we also identified 482 candidate key TF whose reduced expression is associated with the emergence of one or more of 90 cell types (23 ± 26 per cell type; Supplementary Table 9 and Methods). For example, at the split from inner cell mass to epiblast and hypoblast at E4.5, *Gata6* and *Nanog* are identified as decreasing in the respective emergence of the epiblast and hypoblast^69,70^. Also, *Pou5f1* (*Oct4*) was identified as a key TF with reduced expression in association with 20 cell types but increased expression with only one, consistent with its established role in stemness (Supplementary Fig. 15a)^71,72^. In sharp contrast, *Nfia* and *Nfib* (nuclear factors I/a and I/b) were nominated as key TFs at the emergence of 15 and 11 cell types, respectively, but in all cases upregulated, consistent with broad roles in lineage progression^73,74^.

### Core promoter motifs associated with cell-type specification

Although single-cell chromatin accessibility profiling is increasingly enabling the ascertainment of *cis*-regulatory programs in embryonic and fetal tissues^75–77^, such data are not yet available for a dense time course of early mouse development. As a step forward with scRNA-seq data alone, we sought to identify motifs enriched in the core promoters of developmentally regulated genes in TOME. First, we extended the approach described above to nominate key TFs to all genes. This yielded 8,307 ‘key genes’ whose upregulation or downregulation was associated with the emergence of one or more of 92 cell types (470 ± 433 per cell type; Supplementary Fig. 15b and Supplementary Table 10). Second, we applied HOMER (ref. ^78^) to discover motifs enriched in the core promoters of key genes of each cell type. Finally, we estimated *q* values for discovered motifs by data label permutation. At a false discovery rate of 10%, we implicated 119 de novo and 235 known promoter motifs in the emergence of 57 and 34 mouse cell types, respectively (Supplementary Tables 11 and 12).

**Extended Data Fig. 8:**
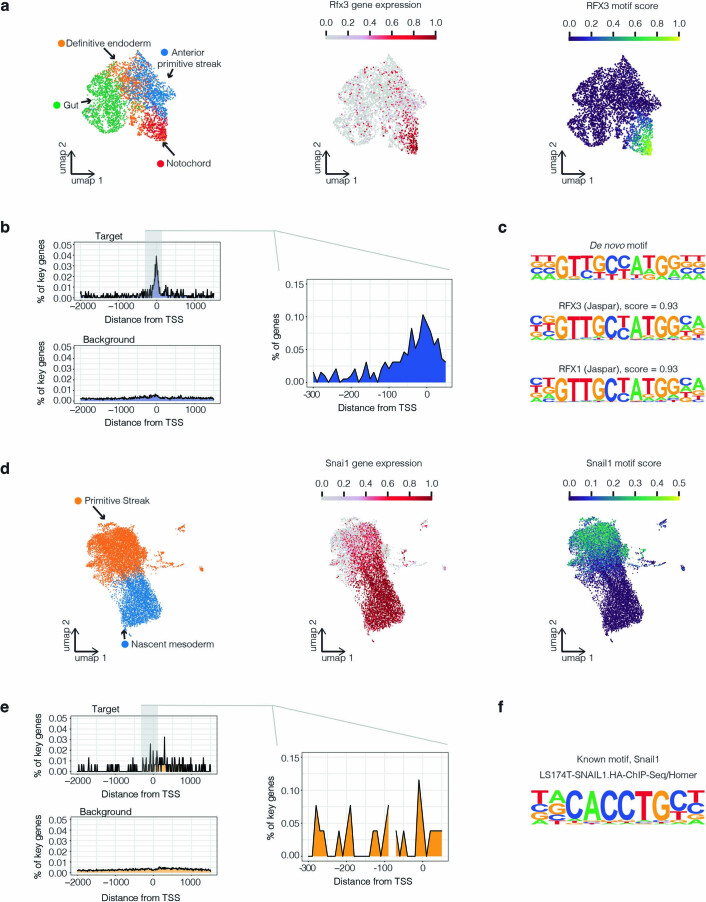
Correlation between key TF expression and up- or downregulation of putative targets of regulation. **a**, UMAP visualization of coembedded cells from cell states including anterior primitive streak, definitive endoderm, gut, and notochord (mouse E7.25 → E7.5) colored by cell type (left), *Rfx3* gene expression (middle) or RFX3 motif score (right), respectively. The RFX3 motif score for each cell was calculated by averaging the gene expression of 135 key genes for notochord emergence bearing this motif in their core promoters, and then subtracting the mean expression of a reference set of randomly sampled genes, using the *score_genes* function of *Scanpy*^101^. **b**, Positional bias of RFX3 binding motif along the core promoters of key genes for notochord emergence (right panel), an expanded region for key genes for notochord emergence (left top panel), or an expanded region for background (left bottom panel). The *y*-axes indicate the % of key genes or background genes with the RFX3 motif with 10 bp bins. **c**, The motif logo of the top *de novo* motif for notochord emergence and its two best alignments in the known motif database. **d**, UMAP visualization of coembedded cells from cell states including primitive streak and nascent mesoderm (mouse E6.5 → E7.25) colored by cell types (left), *Snai1* gene expression (middle) or SNAIL1 motif score (right), respectively. The SNAIL1 motif score was calculated as in panel a, based on 21 key genes for nascent mesoderm emergence bearing this motif in their core promoters. **e**, Positional bias of SNAI1 binding motif along the core promoters of key genes for nascent mesoderm emergence (right panel), an expanded region for key genes for nascent mesoderm emergence (left top panel), or an expanded region for background (left bottom panel). The *y*-axes indicate the % of key genes or background genes with the SNAIL1 motif with 10 bp bins. **f**, The known motif logo of SNAIL1.

We then asked whether these core promoter motifs corresponded to the binding sites of candidate key TFs for the same cell types, which would provide a plausible confirmation of their role. We identified 38 such instances, 33 as positive and 5 as negative correlations (Supplementary Table 13). For example, the transcriptional activator *Rfx3* is sharply upregulated at the emergence of the notochord at E7.25, and its cognate motif is strongly enriched at the promoters of genes upregulated in these same cells (Extended Data Fig. 8a–c) (refs. ^59,79^). In contrast, the transcriptional repressor *Snai1* (Snail) is upregulated at the emergence of nascent mesoderm at E6.75, but its cognate motif is strongly enriched in the promoters of downregulated key genes (Extended Data Fig. 8d–f) (refs. ^80,81^). Interestingly, these enrichments are highly localized near the transcription start site (TSS) for the RFX3 motif but more diffuse for the SNAIL1 motif (Extended Data Fig. 8b,e).

A limitation of these analyses is that we restricted our search for enriched sequence motifs to the core promoters of up- or downregulated key genes. As single-cell, genome-wide chromatin accessibility datasets spanning mouse development are generated, such analyses can be extended to enhancer-mediated regulation.

### Nomination of cell-type homologs among mouse, frog and fish

**Fig. 6:**
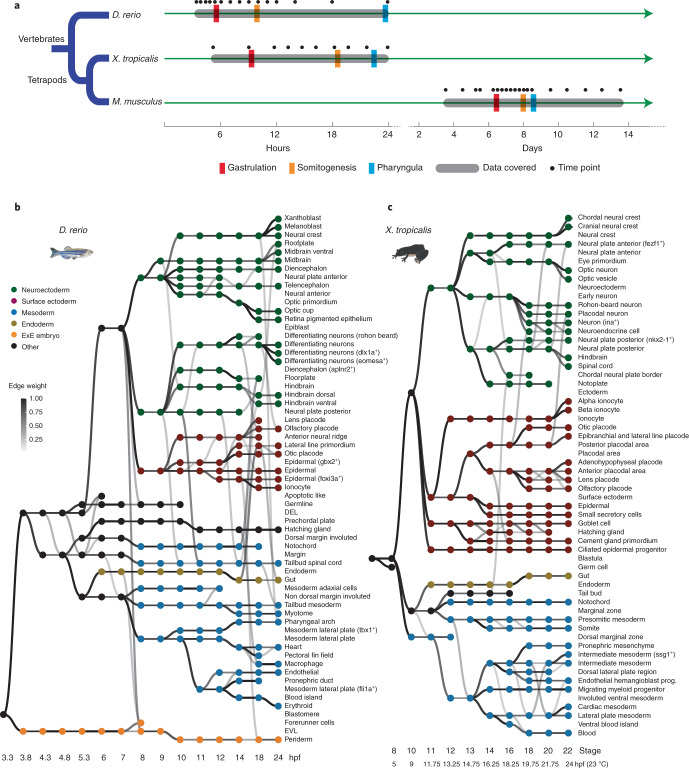
Reconstruction of the cellular trajectories of zebrafish and frog embryogenesis. **a**, Comparative developmental timelines for mouse, zebrafish and frog, spread over two time scales, and approximate (as temperature dependent, particularly for frog). Gastrulation and somitogenesis refer to the timing of onset of these processes^99^. Pharyngula refers to the timing of onset of PA formation^100^. Black dots refer to time points sampled across seven studies. Gray rounded rectangles indicate developmental windows covered by cellular trajectory reconstructions. **b**, Directed acyclic graph showing inferred relationships between cell states across early zebrafish development. Each row corresponds to one of 63 cell-type annotations and columns to developmental stages spanning hours postfertilization 3.3 (hpf3.3) to hpf24. Nodes denote cell states, and node colors denote germ layers. All edge weights greater than 0.2 are shown in grayscale. **c**, Directed acyclic graph showing inferred relationships between cell states across early frog development. Each row corresponds to one of 60 cell-type annotations, columns to developmental stages spanning S8 (hpf5, 23 °C) to S22 (hpf24, 23 °C), nodes to cell states and node colors to germ layers. All edge weights greater than 0.2 are shown in grayscale. DEL, deep cell layer; EVL, enveloping layer.

The origin and evolution of vertebrate cell types are fascinating topics on which the single-cell profiling of embryogenesis may shed light^82^. However, it remains unclear how best to identify ‘cell-type homologs’ across vast evolutionary distances. To facilitate the alignment of cell types across vertebrates, we applied the same strategy used for TOME to zebrafish (*Danio rerio*) and frog (*Xenopus tropicalis*) embryogenesis, again relying on published scRNA-seq datasets (Supplementary Note 6, Fig. 6 and Supplementary Tables 1 and 14–21).

**Fig. 7:**
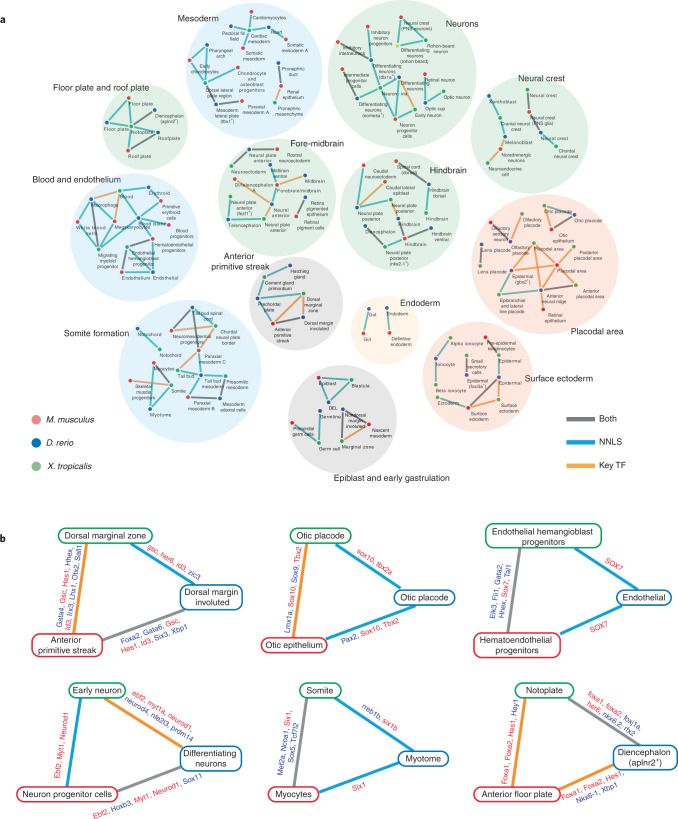
The union of candidate cell-type homologs, identified among three species (mouse, zebrafish and frog) by two strategies. **a**, Candidate cell-type homologs were identified either by comparison of transcriptomes via nonnegative least-squares regression or by examining overlap between upregulated candidate key TFs (key TF). Nominated pairings were manually reviewed, and a subset retained based on biological plausibility. Colors of nodes indicate the species of a given cell type, and colors of edges indicate which approach(es) identifies the pairing. Sets of connected candidate cell-type homologs are further grouped by germ layer or developmental system. **b**, Selected examples of ‘three-way’ pairwise cell-type homology from different germ layers in the above network. Upregulated candidate key TFs shared by each pair of species are listed, with the subset shared by all three species in red font. Of note, key TFs shared by mouse (mm) versus zebrafish (zf) and mm versus frog (xp) are shown by mouse gene symbols, whereas key TFs shared by zf versus xp are shown by zebrafish gene symbols. NNLS, nonnegative least squares.

**Extended Data Fig. 9:**
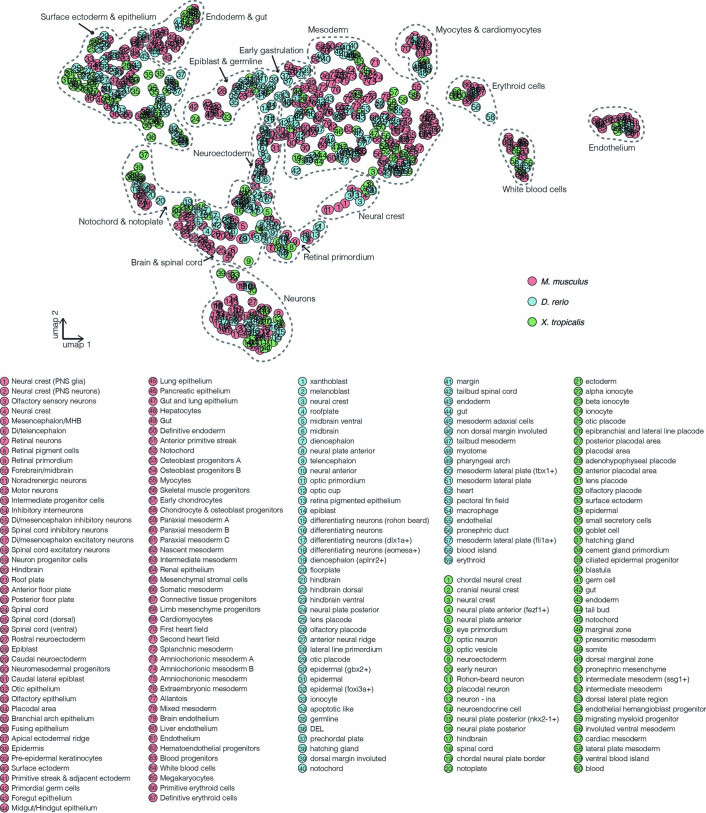
Coembedding of 825 cell states from three species by integrating their transcriptional features. For cell states spanning multiple time points, cells from each time point were treated separately for the purposes of this analysis. To create a transcriptional feature corresponding to each cell state (*that is* a pseudocell), we first averaged cell-state-specific UMI counts, normalized by the total count, multiplied by 100,000 and natural-log-transformed after adding a pseudocount. We then divided all resulting 825 pseudo-cells from the three species into four groups: the mouse single-cell group (n = 151), the mouse single-nucleus group (n = 277), the zebrafish group (n = 205), and the frog group (n = 192), and performed the anchor-based batch correction^13^. UMAP visualization shows coembedded pseudo-cells from the mouse (red), the zebrafish (blue), and the frog (green). Each circle corresponds to a pseudocell, and the numbers correspond to the cell–state labels shown below. The grey dotted curves (manually added) highlight 15 major groups, each including representatives from all three species. Cell states from the extraembryonic lineages (inner cell mass, hypoblast, parietal endoderm, extraembryonic ectoderm, visceral endoderm, embryonic visceral endoderm, and extraembryonic visceral endoderm for the mouse; blastomere, EVL, periderm, forerunner cells for the zebrafish) were excluded from this analysis. For E6.5 of mice, we only used cells from a single study^2^. PNS: peripheral nervous system. MHB: midbrain–hindbrain boundary. Di: diencephalon. DEL: deep cell layer. EVL: enveloping layer.

**Extended Data Fig. 10:**
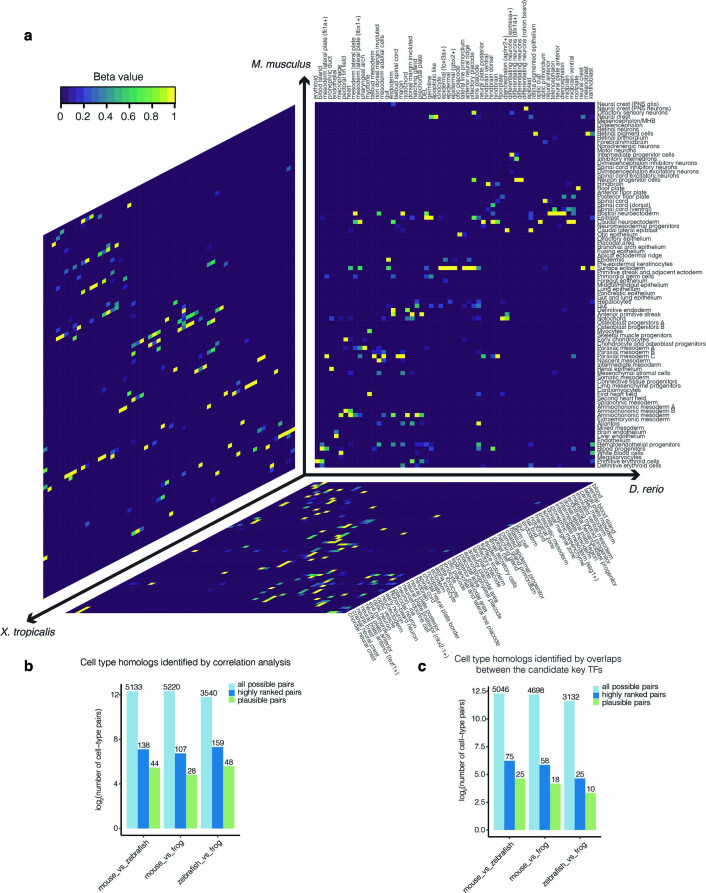
Correlated cell types between species based on nonnegative least-squares regression. **a**, Correlated cell types between each pair of species based on nonnegative least-squares (*NNLS*) regression (Methods). Shown here is a heat map of the normalized *β* values between 87 cell types from the mouse, 59 cell types from the zebrafish, and 60 cell types from the frog. The order of cell types listed in the heat map is the same as each cellular trajectory plot (Fig. 2c; Fig. 6b,c). PNS: peripheral nervous system. MHB: midbrain–hindbrain boundary. Di: diencephalon. DEL: deep cell layer. **b**, The log2-scaled number of all possible pairs, highly ranked pairs, and biologically plausible pairs of cell types evaluated by nonnegative least-squared (*NNLS*) regression. ‘All possible pairs’ refers to all potential cell type pairings considered; ‘highly ranked pairs’ refer to pairings with *β* > 1e-4 and that ranked highly from the perspective of both species; ‘plausible pairs’ refer to pairings which were retained after manual review for biological plausibility (Supplementary Table 23). Actual numbers are shown above each bar, with *y*-axis on log2 scale. **c**, The log2-scaled number of all possible pairs, highly ranked pairs, and biologically plausible pairs of cell types evaluated on the basis of overlapping, orthologous candidate key TFs. ‘All possible pairs’ refers to all potential cell type pairings considered; ‘highly ranked pairs’ refer to pairings with with estimated relative likelihoods more extreme than 99% of permutations; ‘plausible pairs’ refer to pairings which were retained after manual review for biological plausibility (Supplementary Table 24). Actual numbers are shown above each bar, with *y*-axis on log2 scale.

Because mouse (*Mus musculus)* is separated from zebrafish and frog by ~450 million and ~360 million years of evolution, respectively, the identification of cell-type homologs based on cross-species coembedding proved more challenging than is the case for more closely related species such as mouse and human^83,84^ (Extended Data Fig. 9). We therefore attempted two alternative strategies, one based on the comparison of transcriptomes and the other on the comparison of candidate key TFs, resulting in the assignments shown in Fig. 7a (Supplementary Note 6, Extended Data Fig. 10 and Supplementary Tables 22–25). Of note, the set of apparent cell-type homologs was noisy prior to manual filtering; fully automating these assignments remains an outstanding challenge.

Overall, we were able to assign at least one cell-type homolog to 52 of 87 embryonic mouse cell states, 49 of 59 zebrafish embryonic cell states, and 45 of 60 frog embryonic cell states. Some loosely annotated cell types were resolved through homology. For example, zebrafish *eomesa*^+^ and *dlx1a*^+^ differentiating neurons were homologous to mouse intermediate progenitor cells and inhibitory interneurons, respectively. In certain cases, we observed three-way pairwise homology involving a shared candidate key TF (Fig. 7b). For example, *Gsc*, a canonical TF of the Spemann–Mangold organizer^85^, was nominated as a key regulator of the anterior primitive streak (mouse), dorsal margin involuted (zebrafish) and dorsal marginal zone (frog). Other such three-way-nominated TF regulators and associated cell types include *Sox7* for hemogenic endothelium^86^*, Tbx2* for the otic placode^87,88^ and *Six1* for myocytes^89^ (Fig. 7b).

## Discussion

Here, we sought to leverage heterogeneously acquired single-cell transcriptional data to reconstruct a ‘roadmap’ of the trajectories that cells traverse throughout mouse embryogenesis. Although the resulting graph is a highly reductionist representation of mammalian development, we believe that it provides a useful entry point for analyses that benefit from a global view. For example, in addition to nominating specific TFs as potential regulators of the emergence of each cell type, we are able to systematically assess which TFs and genes have relatively specific versus general roles (Fig. 5 and Supplementary Fig. 15a,b), as well as other characteristics (e.g., upregulated key TFs are associated with broad H3K27me3 domains; Supplementary Fig. 15c) (ref. ^90^). Global views also facilitate the identification of ‘cell-type homologs’ through approaches that consider all cell types in each pair of species, analogous to comparative genomics (Figs. 6 and 7).

For integrating time series data collected by destructive methods, the consistency of in vivo development is a terrific feature relative to in vitro systems, which may vary by laboratory, operator, cell line, etc. Of note, by profiling individual embryos staged at one-somite increments around E8.5, we captured rapid, highly coordinated changes in gene expression for some cell types. Extending this higher temporal resolution to the entirety of mouse development, from fertilization to birth, remains an outstanding challenge. TOME also provides a scaffold onto which additional single-cell data types can be layered (e.g., chromatin accessibility, methylation and histone modifications). We are particularly excited about the possibility of linking the temporal unfolding of combinatorial TF expression to enhancer accessibility and then enhancer accessibility to expression of *cis*-regulated genes.

Nearly 40 years ago, Sulston and colleagues painstakingly mapped out the entirety of the invariant embryonic cell lineage of *C. elegans*, comprising 671 cells^1^. The Sulston map provided a foundational scaffold for the integration of future experimental results, as well as a precise nomenclature that facilitates the scholarly discussion of specific cells within the developing worm. Recently, Packer and colleagues intersected the Sulston lineage with the transcriptional profiles of the same cells, shedding fresh light on the relationship between cell states and fates^8^.

Can equivalently comprehensive views be achieved for the developing mouse? For reasons including scale, complexity, variance and accessibility, this is an extraordinary challenge and one that may take decades to fully come to fruition, if indeed it ever does. However, given the pace at which relevant technologies are emerging and evolving, it feels increasingly possible. For example, organism-scale lineage recording, originally developed in zebrafish, has recently been adapted to the mouse^91–94^. Although such methods remain far from delivering the resolution and clarity of the Sulston lineage, they continue to advance from a technical perspective^95,96^. In particular, the concurrent recording of cell lineage and molecular histories may pave the way to more detailed models that explicitly relate patterns of cell division with the unfolding of cell states throughout the developing mouse embryo.

## Methods

### Data reporting

For newly generated E8.5b data, no statistical methods were used to predetermine sample size. Embryos used in experiments were randomized before sample preparation. Investigators were blinded to group allocation during data collection and analysis: embryo collection and sci-RNA-seq3 analysis were performed by different researchers in different locations. All animal use at The Jackson Laboratory was done in accordance with the Animal Welfare Act and the American Veterinary Medical Association Guidelines on Euthanasia, in compliance with the Institute for Laboratory Animal Research Guide for Care and Use of Laboratory Animals, and with prior approval from the animal care and use committee under protocol AUS20028.

### Generating new E8.5 data using an optimized version of sci-RNA-seq3

For newly generated E8.5b data, C57BL/6NJ mice (strain 005304) were used to collect 12 E8.5 embryos (5 males and 7 females) at The Jackson Laboratory. Mice were housed in a barrier research animal facility that maintained a 12-h light/12-h dark light cycle, ambient temperature of 65–75 °F (~18–23 °C) and 40–60% humidity. Noon of the day on which a vaginal plug was observed following overnight mating was defined as E0.5. In brief, timed matings of mice were performed via standard husbandry procedures. On the morning of E8.5, individual deciduae were removed and placed in ice-cold PBS during the harvest. Individual embryos were dissected free of extraembryonic membranes and imaged, and the number of somites present was noted prior to snap freezing in liquid nitrogen (Fig. 1c). Samples were stored at −80 °C until further processing.

We performed a simplified version of sci-RNA-seq3, further optimized for ‘tiny’ samples^4^. Briefly, to each tube, 100 µl of a hypotonic, PBS-based lysis buffer was added with diethyl pyrocarbonate as an RNase inhibitor. The resulting nuclei were then fixed with four volumes of a mix of methanol and dithiobis (succinimidyl propionate). After rehydrating and washing the nuclei carefully in a sucrose/PBS/Triton buffer, the nuclei were distributed to a 96-well plate for reverse transcription (RT), allocating eight wells per embryo. After RT, nuclei were pooled, washed in sucrose/PBS/Triton buffer and redistributed to a fresh plate for ligation of the second index primer with T4 DNA ligase. Nuclei were then again pooled, washed and redistributed to five final plates for second-strand synthesis, extraction, tagmentation and polymerase chain reaction (PCR) to add the third index plus a plate index. Products were pooled by PCR plate, size-selected and sequenced on an Illumina NovaSeq. A more detailed version of the streamlined, tiny sci-RNA-seq3 protocol is available in Martin et al.^14^. The sequences of all oligonucleotides used are provided in Supplementary Table 26.

### Processing of sequencing reads of new E8.5 data

For newly generated E8.5b data, read alignment and gene count matrix generation were performed using the pipeline that we developed for sci-RNA-seq3 (ref. ^4^), with minor modifications; base calls were converted to fastq format using Illumina’s bcl2fastq/v2.20 and demultiplexed based on PCR i5 and i7 barcodes using maximum likelihood demultiplexing package deML (ref. ^102^) with default settings. Downstream sequence processing and single-cell digital expression matrix generation were similar to sci-RNA-seq (ref. ^7^), except that RT index was combined with hairpin adaptor index, and thus the mapped reads were split into constituent cellular indices by demultiplexing reads using both the RT index and ligation index (Levenshtein edit distance (ED) < 2, including insertions and deletions). Briefly, demultiplexed reads were filtered based on RT index and ligation index (ED < 2, including insertions and deletions) and adaptor-clipped using trim_galore/v0.6.5 with default settings. Trimmed reads were mapped to the mouse reference genome (mm10) for mouse embryo nuclei using STAR/v2.6.1d (ref. ^103^) with default settings and gene annotations (GENCODE VM12 for mouse). Uniquely mapping reads were extracted, and duplicates were removed using the UMI sequence (ED <2, including insertions and deletions), RT index, hairpin ligation adaptor index and read 2 end coordinate (i.e., reads with UMI sequence less than 2 ED, RT index, ligation adaptor index and tagmentation site were considered duplicates). Finally, mapped reads were split into constituent cellular indices by further demultiplexing reads using the RT index and ligation hairpin (ED <2, including insertions and deletions). To generate digital expression matrices, we calculated the number of strand-specific UMIs for each cell mapping to the exonic and intronic regions of each gene with Python/v2.7.13 HTseq package^104^. For multimapped reads, reads were assigned to the closest gene, except in cases where another intersected gene fell within 100 bp of the end of the closest gene, in which case the read was discarded. For most analyses, we included both expected-strand intronic and exonic UMIs in per-gene single-cell expression matrices.

After the single-cell gene count matrix was generated, cells with low quality (UMI <200 or detected gene <100 or unmatched_rate ≥0.4) were filtered out, and 239,533 cells were left. Each cell was assigned to its original mouse embryo on the basis of the RT barcode. For the detection of potential doublet cells, we first split the dataset into subsets for each individual and then applied the scrublet/v0.1 pipeline^105^ to each subset with parameters (min_count = 3, min_cells = 3, vscore_percentile = 85, n_pc = 30, expected_doublet_rate = 0.06, sim_doublet_ratio = 2, n_neighbors = 30 and scaling_method = ‘log’) for doublet score calculation. Cells with doublet scores over 0.2 were annotated as detected doublets. We detected 2% potential doublet cells in the whole dataset.

For detection of doublet-derived subclusters for cells, we used an iterative clustering strategy based on Scanpy/v.1.6.0^101^. Briefly, gene count mapping to sex chromosomes were removed before clustering and dimensionality reduction, and then genes with no count were filtered out and each cell was normalized by the total UMI count per cell. The top 1,000 genes with the highest variance were selected and the digital gene expression matrix was renormalized after gene filtering. The data were log transformed after adding a pseudocount and scaled to unit variance and zero mean. The dimensionality of the data was reduced by PC analysis (PCA) (30 components) first and then with UMAP, followed by Louvain clustering performed on the 30 PCs with default parameters. For Louvain clustering, we first fitted the top 30 PCs to compute a neighborhood graph of observations with local neighborhood number of 50 by scanpy.pp.neighbors. We then cluster the cells into subgroups using the Louvain algorithm implemented as scanpy.tl.louvain function. For UMAP visualization, we directly fit the PCA matrix into scanpy.tl.umap function with min_distance of 0.1. For subcluster identification, we selected cells in each major cell type and applied PCA, UMAP, Louvain clustering similarly to the major cluster analysis. Subclusters with a detected doublet ratio (by Scrublet) over 15% were annotated as doublet-derived subclusters.

For data visualization, cells labeled as doublets (by Scrublet) or from doublet-derived subclusters were filtered out. For each cell, we only retain protein-coding genes, long intergenic noncoding RNA genes and pseudogenes. Genes expressed in less than 10 cells and cells in which fewer than 100 genes were detected were further filtered out. The downstream dimension reduction and clustering analysis were done with Monocle/3-alpha. The dimensionality of the data was reduced by PCA (50 components), first on the top 5,000 most highly dispersed genes and then with UMAP (max_components = 2, n_neighbors = 50, min_dist = 0.01, metric = ‘cosine’). Cell clusters were identified using the Louvain algorithm implemented in Monocle/3 (resolution = 1 × 10^−6^). We found that the above Scrublet and iterative clustering based approach is limited in marking cell doublets between abundant cell clusters and rare cell clusters (e.g., less than 1% of total cell population). To further remove such doublet cells, we took the cell clusters identified by Monocle/3, downsampled each cell cluster to 2,500 cells and computed differentially expressed genes (DEGs) across cell clusters with the top_markers function of Monocle/3 (reference_cells = 1,000). We then selected a gene set combining the top ten gene markers for each cell cluster (filtering out genes with fraction_expressing <0.1 and then ordering by pseudo_R2). Cells from each main cell cluster were selected for dimension reduction by PCA (10 components), first on the selected gene set of top cluster specific gene markers and then by UMAP (max_components (the dimensionality of the reduced space) = 2, n_neighbors (the number of neighbors to use during kNN graph construction) = 50, min_dist = 0.1 (the minimum distance to be passed to UMAP function), metric = ‘cosine’), followed by clustering identification using the Louvain algorithm implemented in Monocle/3 (resolution = 1× 10^−4^ for most clustering analysis). Subclusters showing low expression of target cell cluster specific markers and enriched expression of nontarget cell cluster-specific markers were annotated as doublets derived subclusters and filtered out in visualization and downstream analysis. We further filtered out the potential low-quality cells by investigating the numbers of UMIs and the proportion of reads mapping to the exonic regions per cell (Supplementary Fig. 1b,c), resulting in a set of 154,313 cells (median UMI count 7,672; median genes detected 3,463) that were used for reconstructing cellular trajectories.

### Deeper sequencing of previously reported libraries (E9.5–E13.5)

To obtain higher-quality data across E9.5–E13.5, we performed a deeper sequencing (specifically, three additional NovaSeq runs) of previously reported libraries^4^. We merged the new reads with the previous reads and performed the same strategy of data processing that we applied to the newly created E8.5 data. After the single-cell gene count matrix was generated, cells with low quality (UMI <200, detected gene <100 or unmatched_rate ≥0.4) were filtered out, and 2,432,186 cells remained. Compared to the previous data^4^, the median UMI count per cell improved from 671 to 1,434, whereas the median genes detected per cell improved from 518 to 735 (Supplementary Fig. 1a).

Each cell was assigned to its original mouse embryo on the basis of the RT barcode. After removing doublets, we further filtered out potential low-quality cells based on UMI counts and the proportion of reads mapping to the exonic regions per cell (Supplementary Fig. 1b,c), resulting in 1,393,565 cells (median UMI count 1,744; median genes detected 851) that were used for reconstructing cellular trajectories.

### Decoding the transcriptional heterogeneity of NMP cells

To systematically identify cell types whose transcriptional dynamics are most highly correlated with somite counts, we first manually excluded cell types with fewer than 100 cells, and then for each cell type, we calculated the Pearson correlation between cells’ somite counts and those of their top five nearest neighbors in a global 3D UMAP embedding.

We applied two different strategies to identify the genes (among the top 5,000 highly variable genes) that were significantly correlated with the top three PCs of NMP cells. As the first strategy, we performed a generalized linear regression using the fit_models function (model_formula_str = ~individual_PC) in Monocle/3 across the NMP cells. As the second strategy, we performed a Pearson regression between each individual PC and the gene expression values, which were calculated from original UMI counts normalized to total UMI per cell, followed by natural-log transformation. The PCs were calculated on NMP cells only. The significant results (false discovery rate <0.05 and absolute coefficients >0.2 by Pearson correlation) are shown in Supplementary Table 3.

### Systematic reconstruction of the cellular trajectories of mouse embryogenesis

Single-cell or single-nucleus RNA-seq data were collected from three studies from other laboratories^2,9,10^ and supplemented with the new E8.5 data (‘E8.5b’) as well as data from Cao et al.^4^ but supplemented and reanalyzed after deeper sequencing of the same libraries, as described above. These data span 19 time points between E3.5 and E13.5 of mouse embryogenesis, collectively 1,658,968 cells/nuclei from 480 samples, where each sample consists of either a single mouse embryo or a small pool of embryos from the same time point. Further details are provided in Supplementary Table 1. For each dataset, we took the UMI count matrix (feature × cell) from the data source and separated cells by time point. For each time point, we performed conventional scRNA-seq data processing using Seurat/v3: (1) normalizing the UMI counts by the total count per cell followed by log transformation; (2) selecting the 2,500 most highly variable genes and scaling the expression of each to zero mean and unit variance; (3) applying PCA and then using the top 30 PCs to create a *k*-NN graph, followed by Louvain clustering (resolution = 1); (4) performing UMAP visualization in 2D space (dims (which dimensions to use as input features) = 1:30, min_dist = 0.75)^13^. For some time points, we observed obvious batch effects with respect to either study or sample identity. We therefore performed an additional batch correction before the PCA, following the standard pipeline for dataset integration in Seurat/v3 (https://satijalab.org/seurat/v3.2/integration.html), using either the study or sample identity to split datasets, followed by identifying ‘anchors’ between pairs of post-splitting subsets of the datasets (features = 2,500, *k*.filter = 200, dims = 1:30) (Extended Data Fig. 1a,b).

For cell clustering, we manually adjusted the resolution parameter toward modest overclustering and then manually merged adjacent clusters if they had a limited number of DEGs relative to one another (for this purpose, DEGs were defined as genes expressed at mean >0.5 UMIs per cell across the pair of clusters with a more than fourfold difference between the clusters) or if they both highly expressed the same literature-nominated marker genes. Subsequently, we annotated individual cell clusters using two to five literature-nominated marker genes per cell-type label (Supplementary Table 2). Many of the cell-type labels and associated marker genes were obtained from the four studies that generated the data. However, we double checked each cell-type assignment, often with additional marker genes. Importantly, we revisited and revised some of the cell-type or trajectory annotations of Cao et al.^4^ (e.g., ependymal cell→roof plate or isthmic organizer cells→mesencephalon/MHB). A full list of these annotation revisions is provided in Supplementary Table 27. To benchmark the robustness of cell-type annotations, we applied the sklearn.svm.LinearSVC function in scikit-learn/1.0 with fivefold cross-validation using the expression values of all genes as predictors (Supplementary Figs. 5 and 6).

To connect each cell state observed at a given time point with its pseudoancestors, we first merged all cells from that time point and the preceding time point using Seurat/v3. Integration and batch correction were performed as described above, except that we also split based on time point identity (features = 2,500, k.filter = 200, dims = 1:30). Because of the very large number of cells, we used a reciprocal PCA-based space^13^ to find anchors for pairs of time points that included data from (Cao et al.)^4^. After integration, we performed PCA and then used the top 30 PCs to coembed cells as a 3D UMAP (min_dist = 0.75), from which we calculated Euclidean distances between individual cells from the earlier and later time points.

We then determined edge weights between cell states of the successive time points using a bootstrapping strategy. For cells of each cell state at the later time point, we identified their five closest neighbor cells from the earlier time point and then calculated the proportion of these neighbors derived from each potential antecedent cell state. We repeated these steps 500 times with 80% subsampling from the same embedding. We then took the median proportions as the set of weights for edges between a cell state and its potential antecedents. To evaluate the robustness of this approach to the choice of coembedding space, we repeated it using Euclidean distances between cells in PCA space (dims = 30) instead of UMAP space (dims = 3). The resulting edge weights were highly correlated (Pearson correlation coefficient = 0.993). We evaluated the above approach with *k* parameters (for the *k-*NN) other than five and found the resulting edge weights to be highly correlated with those obtained with *k* = 5 (Pearson correlation coefficients from 0.9994 to 0.9999 for *k* = 8, 10, 15 and 20). Edge weights >0.2 from the UMAP embedding were retained for the resulting acyclic directed graph shown in Fig. 2c.

We repeated this strategy to generate similar graphs for zebrafish (*D. rerio*) and frog (*X. tropicalis*) embryogenesis, again relying on publicly available scRNA-seq datasets. For zebrafish, we integrated data from two studies that overlapped at three time points (hpf6, hpf8 and hpf10); we excluded cells from hpf4 because of excessive batch effects^3,6^. For frog, we used cells from a single study^5^. Further details regarding data sources are available in Supplementary Table 1.

### RNA velocity analysis

Three datasets were used in performing RNA velocity analysis: the Pijuan-Sala et al. dataset, the newly generated E8.5 dataset and the dataset resulting from deeper sequencing of Cao et al. 2019 libraries^4^. For the Pijuan-Sala et al. dataset, which was generated on the 10x Genomics platform, we downloaded the raw data (E-MTAB-6967) and reprocessed them using kb-python^106^. For the new E8.5 data as well as the deeper sequencing of Cao et al. 2019 libraries, both generated with sci-RNA-seq3, we processed the raw data using the basic sci-RNA-seq pipeline followed by extracting the spliced reads and unspliced reads for each cell using velocyto^4,45^. The RNA velocity analysis and UMAP visualization were performed with Scanpy/v.1.6.0 and scVelo^97,101^. Briefly, genes with low expression were filtered out (min_counts (minimum number of counts required for a gene to pass filtering (spliced)) = 5, min_counts_u (minimum number of counts required for a gene to pass filtering (unspliced)) = 5), and each cell’s counts were normalized toward the median UMI counts per cell by a scaling factor. The 3,000 genes with the highest variance were selected, and the data were log transformed after adding a pseudocount. The spliced and unspliced count matrices were similarly filtered and normalized. We then applied scvelo.pp.memoments and scvelo.tl.velocity for velocity estimation (n_pcs = 30, n_neighbors = 30), followed by scvelo.tl.velocity_graph and scvelo.tl.umap for data visualization (min_dist = 0.75).

To infer the cell-state transitions between adjacent time points based on RNA velocity, cells from each pair of adjacent time points were integrated, and this was followed by applying the RNA velocity analysis using scVelo^97^. Of note, we did not perform RNA velocity analysis for cell states before E6.5 and during the transition between E8.5a and E8.5b because of limited numbers of cells or the major technological transition, respectively. For cell states from E8.5b onward, we performed a random downsampling on each cell state to 1,500 cells prior to RNA velocity analysis to reduce computational costs. The resulting transition probabilities between individual cells (stored in a velocity_graph matrix), were calculated using cosine correlation between the potential cell-to-cell transitions and the inferred velocity vector (ranging from 0 to 1). To calculate the transition probability from cell state A at the earlier time point to cell state B at the later time point, we summed the transition probabilities of all cells within A to all cells within B, followed by normalizing the total cell number of B. Finally, the edge weight from A to B was further calculated by normalizing their transition probability to the total transition probabilities that originated from A.

### Inferring the molecular histories of individual cell types

For this particular analysis, because one dataset did not include the extraembryonic tissues^4^, we excluded cells annotated as derived from the extraembryonic lineages (embryonic visceral endoderm, extraembryonic visceral endoderm, parietal endoderm and extraembryonic ectoderm). For E6.5, the sequencing depths were very different between datasets, so we only used cells from the Pijuan-Sala et al. dataset. In addition, the Pijuan-Sala et al. dataset pooled multiple embryos per sample, so we used sample identity instead of embryo identity. In the end, four samples from the Cheng et al. dataset, 34 samples from the Pijuan-Sala et al. dataset, 12 samples from the new E8.5 data (E8.5b) and 61 samples from the deeper sequencing of Cao et al. libraries were used for the pseudobulk analysis. UMI counts mapping to each sample were aggregated to generate a pseudobulk RNA-sequencing profile for each sample. We then applied the fit_models function of Monocle/3 to identify genes that were highly correlated with the embryos’/samples’ staged age (model_formula_str = ~stage + dataset). To mitigate major batch effects between cell versus nucleus-derived subsets of the data, we separately performed DEG analysis on the samples from before and including E8.5a (*n* = 34, from Pijuan-Sala et al. dataset) versus including and after E8.5b (*n* = 73), and we then took the union of the top 3,000 genes with the lowest *q* values identified in each subset. We then filtered out genes that were significantly different between the pre- and post-E8.5a/b subsets (*P* value < 0.05). This left 534 genes, which were used to construct a pseudotime trajectory using DDRTree as implemented in Monocle/v*2* (ref. ^107^). Each embryo/sample was assigned a pseudotime value on the basis of its position along the trajectory. Of note, this ordering was highly robust to 80% subsampling (all Pearson correlation coefficients were >0.99 between pseudotimes derived from 100 iterations of 80% subsampling versus the full dataset).

### Deconvolution of cell composition of GEO-seq sample using CIBERSORTx

This analysis was performed by running deconvolution on each GEO-seq sample using CIBERSORTx with default parameters^48,49^. GEO-seq samples were collected from distinct spatial positions in the mouse embryo with mixed cell populations from E5.5, E6, E6.5, E7 and E7.5^48^. For each stage, we first learned a gene expression signature for each cell state at the corresponding time point. Because single-cell profiles from E6 were missing from the scRNA-seq data integrated here, we used data from E6.25 instead.

### Systematic nomination of key TFs for cell-type specification

The list of 1,636 mouse proteins that are putatively TFs was collated from AnimalTFDB/v3 (http://bioinfo.life.hust.edu.cn/AnimalTFDB/)^108^. For each edge in TOME at which a given cell type first emerged, we used three criteria to identify key TF candidates: (1) its expression significantly increased in the newly emerged cell type relative to the pseudoancestral cell state (Seurat/v3; adjusted *P* value < 0.05, nonparametric Wilcoxon rank-sum test), (2) it was significantly more highly expressed in the newly emerged cell type relative to its sister edges deriving from the same pseudoancestor (by the same test and threshold) and (3) it was detected in at least 10% of cells of the newly emerged cell type. For each such candidate key TF, we scaled its log fold-change calculated by either criterion 1 or criterion 2 to unit variance and zero mean (across the set of candidate key TF identified for a given newly emerged cell type) and then averaged these scaled fold-change values to determine a score intended to convey its importance relative to other candidate key TFs for the same cell type.

To identify TFs whose reduced expression was associated with the emergence of each cell type, we looked for those that (1) were detected in at least 10% of cells of the pseudoancestral cell type, (2) were significantly downregulated in the newly emerged cell type relative to the pseudoancestor (Seurat/v3; adjusted *P* value < 0.05, nonparametric Wilcoxon rank-sum test) and (3) were both detected in at least 10% of cells and significantly more highly expressed at sister edges relative to the newly emerged cell type (by the same test and threshold).

The list of 2,547 zebrafish TFs and 1,236 frog TFs was collated from AnimalTFDB/v3 (http://bioinfo.life.hust.edu.cn/AnimalTFDB/)^108^. Candidate key TFs for each cell-type emergence in these species were identified and scored as described above for mouse.

### Coembedding of cell states from three species

We first created a list of orthologous genes across the three species by liftover of all gene identities from the three species to the corresponding human gene identities based on either BioMart (Ensembl Genes 102)^109^ or the original study in the case of frog^5^. A list of 22,815 genes was compiled, wherein each of the genes was orthologous in at least two species. Of note, we retained all of the possible orthologous gene pairs learned from BioMart, including ‘1-to-1’, ‘1-to-many’ and ‘many-to-many’ categories. To create the transcriptional features of each cell state, we first averaged cell-state-specific UMI counts, normalized by the total count, multiplied by 100,000 and natural-log-transformed after adding a pseudocount. We then divided all the cell states from three species into four groups: the mouse single-cell group (*n* = 151), the mouse single-nucleus group (*n* = 277), the zebrafish group (*n* = 205) and the frog group (*n* = 192). We treated each cell state as a pseudocell, performing the anchor-based batch correction approach implemented by *Seurat*/v3 (n_features = 5,000, *k*.filter = 100, dims = 1:30, min_dist = 0.6) (ref. ^13^). For cell states spanning multiple time points, cells from each time point were treated as a separate pseudocell for the purposes of this analysis.

### Identification of interspecies correlated cell types using nonnegative least-squared regression

We first created a list of orthologous genes between each pair of species (*n* = 17,333 for *mm* versus *zf*, *n* = 14,249 for *mm* versus *xp* and *n* = 13,326 for *zf* versus *xp*) based on either BioMart (Ensembl Genes 102)^109^ or the original study in the case of frog^5^. Of note, we retained all of the possible orthologous gene pairs learned from BioMart, including 1-to-1, 1-to-many and many-to-many categories. To identify correlated cell types between each pair of species, we first calculated an expression value for each gene in each cell type by averaging the log-transformed normalized UMI counts of all cells of that type across all time points at which the cell type appeared. Extraembryonic cell types (inner cell mass, hypoblast, parietal endoderm, extraembryonic ectoderm, visceral endoderm, embryonic visceral endoderm and extraembryonic visceral endoderm for the mouse; blastomere, enveloping layer, periderm and forerunner cells for the zebrafish) were excluded from this analysis. For mouse E6.5, we only used cells from a single study^2^. For each pair of species, we took homologous genes and applied nonnegative least-squares regression to predict gene expression in target cell type (*T*_*a*_) in dataset A based on the gene expression of all cell types (*M*_*b*_) in dataset B, *T*_*a*_ = *β*_*0a*_ + *β*_*1a*_*M*_*b*_, based on the union of the 1,200 most highly expressed genes and 1,200 most highly specific genes in the target cell type. We then switched the roles of datasets A and B; that is, predicting the gene expression of target cell type (*T*_*b*_) in dataset B from the gene expression of all cell types (*M*_*a*_) in dataset A, *T*_*b*_ = *β*_*0b*_ + *β*_*1b*_*M*_*a*_. Finally, for each cell type *a* in dataset A and each cell type *b* in dataset B, we combined the two correlation coefficients, *β* = 2(*β*_*ab*_ + 0.001)(*β*_*ba*_ + 0.001), to obtain a statistic for which high values reflect reciprocal, specific predictivity.

To identify candidate cell-type homologs, we manually reviewed pairings with a *β* score >1 × 10^−4^ that ranked highly from the perspective of both species (i.e., where cell type B was one of the top five matches for cell type A and vice versa). We next performed a manual selection based on the following criteria: (1) excluding pairs of cell types which derive from different germ layers or major groups (Extended Data Fig. 9) (e.g., blood progenitors (*mm*) versus optic cup (*zf*)); (2) excluding pairs of cell types that emerged at very different temporal stages (e.g., rostral neuroectoderm (*mm*) versus DEL (*zf*)); (3) excluding cell types only expected in one species or the other (e.g., hatching gland (*zf*) is not expected in mouse); (4) for cell types that were correlated with multiple cell types with ancestor-descendant relationships in the other species, we selected the one that was more ancestral (e.g., hindbrain (*mm*) was correlated with both hindbrain ventral (*zf*) and hindbrain (*zf*), and we assigned it to hindbrain (*zf*)); (5) for cell types that were correlated with multiple cell types in the other species that lacked a clear ancestor–descendant relationship, we selected the pair with the highest *β* score. The details of manual selection are provided in Supplementary Table 23.

### Identification of correlated cell types between species based on overlapping key TF candidates

For each possible interspecies pairing of cell types, we identified orthologous TFs that were nominated in both species and then calculated, as an estimate of relative likelihood, the product of the frequencies in which each of these TFs were nominated as key in their respective species (to account for the fact that some TFs are nominated in many cell types and therefore more likely to overlap; Fig. 5c). To identify which such instances were potentially significant, we repeated these procedures after taking random samples of key TFs without replacement (10,000 times) and retained pairings with estimated relative likelihoods more extreme than 99% of permutations. We then performed a similar manual selection, details of which are provided in Supplementary Table 24.

Of note, we also attempted interspecies cell-type pairing using key genes instead of key TFs for each cell type (Supplementary Table 28). However, the correlated cell types identified by overlapping key genes were noisier than other approaches. For example, anterior floor plate (mm) was correlated to diencephalon (*aplnr2*^+^) (zf) as expected, but it was also correlated to seven other cell types from zebrafish, including erythroid, midbrain ventral, myotome, diencephalon, roof plate, mesoderm lateral plate (*tbx1*^+^) and dorsal margin involuted. As the other strategies appeared less noisy and therefore easier to manually curate, we did not carry this third approach forward.

We compared our cell-type alignments between zebrafish versus frog to a recent study^110^ that also sought to align the same datasets. We could find consistent alignments for 35 of 46 pairs of cell types that they identified (Supplementary Table 29). Note that neither we nor they simply used the original data and annotations, but we reprocessed them in different ways. For example, we combined scRNA-seq data from two zebrafish studies^3,6^ followed by reannotation of the merged set of cells from each individual time point, whereas the other study sometimes merged multiple cell types into one (optic cup and retina pigmented epithelium→optic). These differences make a full comparison challenging. Nonetheless, at least on a high-level check, these entirely independent efforts are mostly in agreement, which is encouraging.

### Identification of *cis-*regulatory motifs involved in in vivo cell-type specification

As a first step toward identifying *cis-*regulatory motifs involved in cell-type identification, we extended to all genes the approach described above to nominate key TFs whose upregulation or downregulation is associated with the emergence of each cell type. For each edge in TOME at which a given cell type first emerged, we used three criteria to identify key gene candidates: (1) its expression significantly increased in the newly emerged cell type relative to the pseudoancestral cell state (Seurat/v3; adjusted *P* value < 0.05, nonparametric Wilcoxon rank-sum test), (2) it was significantly more highly expressed in the newly emerged cell type relative to its sister edges deriving from the same pseudoancestor (by the same test and threshold) and (3) it was detected in at least 10% of cells of the newly emerged cell type. To identify genes whose reduced expression was associated with the emergence of each cell type, we looked for those that (1) were detected in at least 10% of cells of the pseudoancestral cell type, (2) were significantly downregulated in the newly emerged cell type relative to the pseudoancestor (Seurat/v3; adjusted *P* value < 0.05, nonparametric Wilcoxon rank-sum test) and (3) are both detected in at least 10% of cells and significantly more highly expressed at sister edges relative to the newly emerged cell type (by the same test and threshold).

We used HOMER/v4.11 (ref. ^78^) to identify DNA sequence motifs that are specifically enriched in the core promoters of key genes (−300 to +50 bp of annotated TSSs). Running the findMotifs.pl function with default parameters, each test set was defined as the core promoters of either upregulated or downregulated key genes at specific cell edges (excluding sets with fewer than five key genes) and compared to a background set of core promoters of key genes from all edges not in the test set. Motif quality was evaluated based on a *q* value, which was calculated for each motif by 100 iterations of randomizing data labels and rerunning HOMER. In addition, motifs were aligned to known motif binding sequences based on the JASPAR and internal HOMER databases with default parameters^111^. Mapping of specific motif positions around the TSS was assessed with the HOMER function annotatePeaks.pl using the following parameters: tss mm10 -hist 10 -ghist.

### Reporting Summary

Further information on research design is available in the Nature Research Reporting Summary linked to this article.

## Online content

Any methods, additional references, Nature Research reporting summaries, source data, extended data, supplementary information, acknowledgements, peer review information; details of author contributions and competing interests; and statements of data and code availability are available at 10.1038/s41588-022-01018-x.

## Supplementary information

Supplementary Information Supplementary Notes 1–6 and Figures 1–15.

Reporting Summary.

Peer Review Information.

Supplementary Table 1 Supplementary Tables 1–29.

## Data Availability

All data have been made freely available via http://tome.gs.washington.edu. The data generated in this study can be downloaded in raw and processed forms from the National Center for Biotechnology Information (NCBI) Gene Expression Omnibus (GEO) under accession numbers GSE186069 (new E8.5 data) and GSE186068 (deeper sequencing of Cao et al. libraries). The following publicly available datasets were used in this project: AnimalTFDB/v3 (http://bioinfo.life.hust.edu.cn/AnimalTFDB/), the mouse gastrulation dataset generated by Pijuan-Sala et al. (https://github.com/MarioniLab/EmbryoTimecourse2018 and ArrayExpress (E-MTAB-6967)), the mouse pregastrulation dataset generated by Mohammed et al. (NCBI GEO (GSE100597)), the mouse pregastrulation dataset generated by Cheng et al. (NCBI GEO (GSE109071)), the zebrafish embryogenesis dataset generated by Farrell et al. (NCBI GEO (GSE106587)), the zebrafish embryogenesis dataset generated by Wagner et al. (NCBI GEO (GSE112294)) and the frog embryogenesis dataset generated by Briggs et al. (NCBI GEO (GSE113074)).

## Code availability

The Python and R codes used to analyze the RNA-sequencing data are available at https://github.com/ChengxiangQiu/tome_code. The following common, freely available data analysis software packages were used in this project: bcl2fastq version 2.20 (https://support.illumina.com), deML version 1.1.3 (https://github.com/grenaud/deML), HTseq version 0.6.1 (https://github.com/htseq/htseq), trim_galore version 0.6.5 (https://github.com/FelixKrueger/TrimGalore), STAR version 2.6.1d (https://github.com/alexdobin/STAR), scrublet version 0.1 (https://github.com/swolock/scrublet), Scanpy version 1.6.0 (https://github.com/theislab/scanpy), Monocle version 2, 3, and 3-alpha (https://cole-trapnell-lab.github.io/monocle3), DDRTree version 0.1.5 (https://github.com/cole-trapnell-lab/DDRTree), Seurat version 3 (https://github.com/satijalab/seurat), scikit-learn version 1.0 (https://github.com/scikit-learn/scikit-learn), kb-python version 0.25.0 (https://github.com/pachterlab/kb_python), velocyto version 0.6 (https://github.com/velocyto-team/velocyto.py), CIBERSORTx (https://github.com/ysuzukilab/Cibersortx) and HOMER version 4.11 (https://github.com/IGBIllinois/HOMER).
