# Supplementary material for: Systematic reconstruction of cellular trajectories across mouse embryogenesis
Source: Nat Genet. 2022 Mar 14;54(3):328–41. doi: 10.1038/s41588-022-01018-x (PMC8920898; doi:10.1038/s41588-022-01018-x)
Supplement: Supplementary file 1 — Supplementary Notes 1–6 and Figures 1–15. [file 41588_2022_1018_MOESM1_ESM.pdf]

nature portfolio

https://doi.org/10.1038/s41588-022-01018-x

Supplementary information

Systematic reconstruction of cellular trajectories across mouse embryogenesis

In the format provided by the authors and unedited

Systematic reconstruction of cellular trajectories across mouse embryogenesis

Supplementary Notes

Supplementary Note 1

Nuclei from each E8.5 somite-staged embryo were deposited to individual wells (8 wells per embryo) for sci-RNA-seq3, such that the first index identified the originating embryo of any given cell, and then processed in a single experiment. This contrasts with the study of Pijuan-Sala and colleagues[1], in which multiple E8.5 embryos were pooled prior to profiling. The optimized sci-RNA-seq3 method[2] markedly improved data quality, with 9-fold higher UMIs and 6-fold higher gene detection per nucleus, relative to the original protocol[3] (**Supplementary Fig. 1a**). Even after deeper sequencing of the original libraries from that study[3] to a similar duplication rate, the improvement remained substantial (4-fold higher for UMI counts per nucleus; **Supplementary Fig. 1a**).

Supplementary Note 2

For each pair of adjacent stages, we performed anchor-based batch correction followed by projecting cells into a shared embedding space[4]. After co-embedding, we applied a *k*-nearest neighbor (*k*-NN) based heuristic to connect cell states between adjacent stages. Briefly, for each cell state at the later timepoint, we identified the 5 closest cells from the antecedent timepoint in the co-embedding. Bootstrapping to obtain a robust estimate (500 iterations with 80% subsampling), we then calculated the median proportion of such neighbors derived from each potential antecedent cell state, and treated this as the weight of the corresponding edge.

Supplementary Note 3

Of note, we introduced 4 “dummy nodes”, corresponding to morula at E3.0 (as a root for trophectoderm and inner cell mass), trophectoderm at E3.5 and E4.5 (which had been removed at these timepoints by immunosurgery[5]) and parietal endoderm at E6.75 (undetected, likely due to undersampling). For technical reasons (see above), we also introduced an edge between primitive erythroid cells at E8.5a and E8.5b.

Supplementary Note 4

An important limitation of our heuristic approach, made apparent by a few clear inaccuracies in the graph, is that true lineage relationships for a given cell state can be obscured by the presence of a highly similar cell state at the preceding timepoint. For example, E9.5 neuron progenitor cells are assigned as the pseudo-ancestor of multiple neuronal subtypes that appear at E10.5, but we do not observe these same relationships to recur at subsequent timepoints, although neuronal differentiation is surely ongoing. This is probably because at timepoints subsequent to E10.5, each derivative neuronal subtype is most similar to itself at the preceding timepoint, such that it fails to be linked back to the persisting neuron progenitors. This same phenomenon probably explains another error, wherein when definitive erythroid cells first appear at E10.5, they are linked to E9.5 blood progenitors (expected) but also to E9.5 primitive erythroid cells (unexpected). Another example involves motor neurons, which are most closely related to the hindbrain and spinal cord when E9.5 is looked at in isolation (expected), but to the forebrain/midbrain when integrating with E8.5 (unexpected). In this case, the error would likely require sampling at higher temporal resolution in order to correct. For a more exhaustive consideration of the ways in which trajectory-based inference can be misleading about cell lineage histories, see (Wagner and Klein 2020)[6].

Of note, at least some of the inaccuracies noted above are resolvable by focused analyses that leverage the distinction between nascent and spliced transcripts, *i.e.* RNA velocity[7]. For example, if we reanalyze these problematic subsets of TOME with *scVelo*[8], the heterogeneity and ongoing contributions of neuron progenitors are more evident[9] (**Fig. 3a**), and primitive and definitive hematopoiesis are much more clearly separated (**Fig. 3b**). To approach this more systematically, we calculated edge weights between cell states at adjacent timepoints with an alternative heuristic that was based on RNA velocity (**Methods**). We observed that out of 515 edges with weights > 0.2 that were nominated by the *k*-NN strategy, 392 had velocity-based transition probabilities > 0.2 (76%) (**Supplementary Fig. 10-11**; **Extended Data Fig. 6; Supplementary Table 5**). However, there were also 123 edges nominated by the *k*-NN strategy only, and 75 edges nominated by the RNA velocity strategy only (**Extended Data Fig. 6c**). Although we may assign greater confidence to edges nominated by both methods, edges supported by one method or the other may include both true and false positives. As an example of a likely true positive supported by RNA velocity only, the connection between embryonic visceral endoderm (E8.0) and gut (E8.25), fell short of the edge threshold by the *k*-NN strategy (weight 0.14) but was strongly supported by the RNA velocity strategy (weight 0.96).

Supplementary Note 5

We sought to infer continuous expression levels for individual genes over the course of each cellular trajectory, focusing on derivatives of the epiblast from E6.25 onwards. First, we leveraged the fact that individual embryos do not correspond precisely to their intended timepoints. Using pseudotime, we ordered the pseudobulk expression profiles of individual embryos, or pools of embryos comprising each sample, in the case of (Pijuan-Sala *et al.*)[1]. The resulting ordering, which is robust to downsampling, corresponds well with developmental age but may additionally distinguish earlier vs. later individuals/pools at each intended timepoint (**Supplementary Fig. 12a-d**).

Next, for each epiblast-derived cell type that was detectable at E13.5, we calculated a smoothed expression profile along its inferred history, as illustrated in **Supplementary Fig. 12e** for selected genes in one cell type from each germ layer. Despite including the data source as a covariate, these inferred

trajectories remained modestly confounded by batch effects across E8.5a → E8.5b, *i.e.* the switch from cell-based 10X Genomics data to nucleus-based sci-RNA-seq3 data (**Supplementary Fig. 13-14**). Nonetheless, at least anecdotally, TFs with established roles in a given cell type were often upregulated in association with their first appearance (**Supplementary Fig. 12e**)
.

Supplementary Note 6

For zebrafish, we integrated data from two studies that used different technologies but together included 15 developmental stages, beginning at the high stage (hpf 3.3) and ending at the early pharyngula stage (hpf 24), essentially spanning epiboly and segmentation (**Fig. 6a**; **Supplementary Table 1**)[10,11]. The resulting graph contains 221 nodes, each assigned one of 63 cell type annotations, and 257 edges with weights greater than 0.2 (**Fig. 6b**; http://tome.gs.washington.edu). Marker genes used to annotate cell types are provided in **Supplementary Table 14**, and all edge weights in **Supplementary Table 15**. We also nominated key upregulated and downregulated TFs using the same approach described for mouse development (**Supplementary Tables 16-17**).

For frog, we re-analyzed one dataset spanning 10 developmental stages, from S8 and S22[12], spanning gastrulation and neurulation (**Fig. 6a**; **Supplementary Table 1**). The resulting graph contains 192 nodes, each assigned one of 60 cell type annotations, and 221 edges with weights greater than 0.2 (**Fig. 6c**; http://tome.gs.washington.edu). Marker genes used to annotate cell types are provided in **Supplementary Table 18**, all edge weights in **Supplementary Table 19**, and candidate key TFs in **Supplementary Tables 20-21**.

In our original attempt, treating cells of each state from each timepoint as a "pseudo-cell", we integrated data from all three species with anchor-based batch correction[4]. Within the resulting UMAP co-embedding of 825 pseudo-cells, we could identify 15 major groups — epiblast & germline, early gastrulation, neuroectoderm, surface ectoderm & epithelium, mesoderm, notochord & notoplate, endoderm & gut, retinal primordium, neural crest, brain & spinal cord, neurons, endothelium, myocytes & cardiomyocytes, white blood cells and erythroid cells — each containing cell states from all three species (**Extended Data Fig. 9**). However, within each such major group, the homology between specific cell types generally remained ambiguous.

We therefore pivoted to two additional strategies, the results of which are presented in **Fig. 7**.

1) 'nnls' strategy: Here we performed all possible pairwise comparisons between the transcriptomes of cell types of each pair of species, excluding extraembryonic lineages[13]. First, we performed cell type correlation analysis[3], which uses a regression framework to ask, between each pair of species, which cell types are the best reciprocal best matches to one another (**Extended Data Fig. 10a**; **Supplementary Table 22**; **Methods**). We then manually reviewed the highest ranking cell type pairings for biological plausibility. For mouse vs. zebrafish, out of 5,133 pairings tested, 138 were highly ranked, of which we selected 44 as the most biologically plausible (**Supplementary Table 23**). Exclusion criteria included the cell types arising from different germ layers or major groups (as defined in **Extended Data Fig. 9**), arising at very different temporal stages, or if a cell type was exclusive to one species. In cases where multiple related matches were observed, we generally selected the match with the highest β score. Applying this

same approach to mouse vs. frog and zebrafish vs. frog, we identified 28 and 48 plausible cell type homologs pairings, respectively (**Extended Data Fig. 10b**; **Supplementary Table 23**).

2) <u>'key TF' strategy</u>: Here we focused on overlaps between the candidate key TFs associated with the emergence of each cell type in each species. For each possible interspecies pairing of cell types, we identified orthologous TFs that were nominated in both, and then adopted a permutation approach to identify instances in which an excess of orthologous candidate key TFs were shared between the cell types. For mouse vs. zebrafish, out of 5,046 pairings tested, 75 exhibited more sharing than >99% of permutations, of which we retained 25 as the most biologically plausible (**Supplementary Table 24**). Applying this same approach to mouse vs. frog and zebrafish vs. frog, we identified 18 and 10 plausible cell type homolog pairings, respectively (**Extended Data Fig. 10c**; **Supplementary Table 24**).

Some candidate cell type homologs overlapped between the 'nnls' and 'key TF' strategies (**Fig. 7a**; **Supplementary Table 25**).

<u>Supplementary Figure Legend</u>

Supplementary Fig. 1 | Higher quality sci-RNA-seq3 data generated by either application of an optimized protocol (E8.5b) or deeper sequencing of previously reported libraries (E9.5 - E13.5). a, The cell number, median UMI count per cell, median genes detected per cell, and duplicate rate, are shown for a previously published dataset on E9.5 - E13.5 embryos (light blue bars)[3], deeper sequencing and reanalysis of those same sequencing libraries (dark blue bars) or data newly generated on E8.5 embryos using an optimized sci-RNA-seq3 protocol (green bars). The optimized protocol markedly outperforms the previous protocol in terms of the numbers of UMIs obtained and genes detected per cell, even after deeper sequencing of the Cao *et al.*[3] libraries to a similar duplication rate. **b,** Histograms of log2(UMI count) per cell for the newly created E8.5 dataset (left) and more deeply sequenced Cao *et al.*[3] libraries (right). Upper and lower thresholds used for quality filtering, which correspond to the mean +/- 2 standard deviations of log2-scaled values, after excluding cells with >85% of reads mapping to exonic regions (except for the lower bound of 800, which was manually assigned), are shown with dotted vertical lines. **c,** Histograms of the proportion of reads mapping to the exonic regions per cell for the newly created E8.5 dataset (left) and more deeply sequenced Cao *et al.*[3] libraries (right). Cells with greater than 85% of reads mapping to exonic regions were excluded.

Supplementary Fig. 2 | Anterior and posterior floor plate subpopulations emerging from forebrain/hindbrain and spinal cord, respectively. a, Subview of global 3D UMAP visualization highlighting anterior (red) and posterior (blue) floor plate subpopulations in E8.5 data generated with optimized sci-RNA-seq3 protocol. **b,** Re-embedded 2D UMAP of cells from anterior floor plate and posterior floor plate. **c,** The same UMAP as in panel b, colored by somite counts. **d,** The same UMAP as in panel b, colored by gene expression of marker genes which are shared by anterior and posterior floor plate subpopulations. **e,** The same UMAP as in panel b, colored by gene expression of marker genes which appear specific to anterior floor plate subpopulation. **f,** The same UMAP as in panel b, colored by gene expression of marker genes which are specific to posterior floor plate subpopulation. Gene

expression values shown in panel d-f were calculated by normalizing the UMI counts by the estimated size factors followed by log10-transformation.

Supplementary Fig. 3 | Resolution of the first and second heart fields during early somitogenesis. a, Subview of global 3D UMAP visualization highlighting the first (red) and second (blue) heart fields in E8.5 data generated with optimized sci-RNA-seq3 protocol. **b,** Re-embedded 2D UMAP of cells from the first and second heart fields. **c,** The same UMAP as in panel b, colored by somite counts. **d,** The same 2D UMAP as in panel b, colored by gene expression of marker genes which are shared by the first and second heart fields. The same genes are highlighted in the 3D subview of panel a are shown below. **e,** The same UMAP as in panel b, colored by gene expression of marker genes which are specific to the first heart field. The same genes are highlighted in the 3D subview of panel a are shown below. **f,** The same UMAP as in panel b, colored by gene expression of marker genes which are specific to the second heart field. The same genes are highlighted in the 3D subview of panel a are shown below. Gene expression values shown in panel d-f were calculated by normalizing the UMI counts by the estimated size factors followed by log10-transformation.

Supplementary Fig. 4 | Three distinct subpopulations of neural crest cells (NCCs) may correspond to mesencephalic NCCs and pharyngeal arch (PA) contributions. a, Subview of global 3D UMAP visualization highlighting the three subpopulations of NCCs. **b,** Re-embedded 2D UMAP of cells annotated as forebrain, midbrain, presumptive cerebellum, r1 - r6 rhombomeres, spinal cord and neural crest, with highlighting of the three subpopulations of NCCs. **c,** The same 2D UMAP as in panel b, colored by gene expression of marker genes which are shared by the three subpopulations of NCCs. **d,** The same UMAP as in panel b, colored by gene expression of Hox genes used for rough annotation of three subpopulations of NCCs. NC1 (which may correspond to mesencephalic and PA1 NCCs): *Hox*-; NC2 (which may correspond to PA2 NCCs): *Hoxb1*+, *Hox2*+, *Hox3*-; NC3 (which may correspond to PA3 NCCs): *Hox3*+, *Hox4*-[14]. **e,** The same UMAP as in panel b, colored by gene expression of marker genes which are specific to NC1 (*Cdh19*, *Cped1*, *Ebf2*), NC2 (*Ret*), or NC3 (*Lmx1a*). Gene expression values shown in panel c-e were calculated by normalizing the UMI counts by the estimated size factors followed by log10-transformation.

Supplementary Fig. 5 | Benchmarking of the robustness of cell type annotations (E4.5 - E8.5a). We applied the *sklearn.svm.LinearSVC* function in *scikit-learn*/1.0 with 5-fold cross-validation, using the expression values of all genes as predictors. Each heatmap shows the confusion matrix between true cell-type labels (rows) and predicted cell-type labels (columns) for cells within each individual timepoint, normalized to total counts per column (*i.e.* each column sums to one). The accuracy (*Acc*) across the whole matrix is shown above each heatmap.

Supplementary Fig. 6 | Benchmarking of the robustness of cell type annotations (E8.5b - E13.5). We applied the *sklearn.svm.LinearSVC* function in *scikit-learn*/1.0 with 5-fold cross-validation, using the expression values of all genes as predictors. Each heatmap shows the confusion matrix between true cell-type labels (rows) and predicted cell-type labels (columns) for cells within each individual timepoint, normalized to total counts per column (*i.e.* each column sums to one). The accuracy (*Acc*) across the whole matrix is shown above each heatmap. PNS: peripheral nervous system. MHB: midbrain-hindbrain boundary. Di: Diencephalon.

Supplementary Fig. 7 | Inference of epiblast derivatives between E6.25 and E7.0. a, A portion of the UMAP corresponding to the epiblast and its inferred derivatives is shown for co-embeddings of E6.25 → E6.5 (left column), E6.5 → E6.75 (middle column) and E6.75 → E7.0 (right column). Within each column is the same UMAP visualization, but showing only cells from the earlier timepoint (top row), the later timepoint (middle row) or both timepoints (bottom row). **b,** Directed acyclic graph showing inferred relationships between cell states amongst early epiblast derivatives. All edges with weights above 0.2 are shown in grey scale.

Supplementary Fig. 8 | Heatmap of edge weights between cell states at each pair of adjacent timepoints (E3.5 - E8.5a). Each heatmap shows edge weights between all cell states at a given timepoint (rows) and potential pseudo-ancestral cell states from the immediately preceding timepoint (columns). Edge weights were calculated based on a *k*-nearest neighbor (*k*-NN) based heuristic that was applied to a co-embedding of separately annotated cells from the adjacent timepoints. The edge weights range from 0 to 1, and edges with weights greater than 0.2 were carried forward.

Supplementary Fig. 9 | Heatmap of edge weights between cell states at each pair of adjacent timepoints (E8.5a - E13.5). Each heatmap shows edge weights between all cell states at a given timepoint (rows) and potential pseudo-ancestral cell states from the immediately preceding timepoint (columns). Edge weights were calculated based on a *k*-nearest neighbor (*k*-NN) based heuristic that was applied to a co-embedding of separately annotated cells from the adjacent timepoints. The edge weights range from 0 to 1, and edges with weights greater than 0.2 were carried forward. PNS: peripheral nervous system. MHB: midbrain-hindbrain boundary. Di: Diencephalon.

Supplementary Fig. 10 | RNA velocity-based inference of potential cell state relationships across pairs of adjacent timepoints (E6.5 - E8.25). Briefly, we integrated cells from each pair of adjacent timepoints, and then applied RNA velocity analysis using *scVelo*[8]. The resulting transition probabilities between individual cells (stored in a *velocity_graph* matrix), were calculated using cosine correlation between the potential cell-to-cell transitions and the inferred velocity vector (ranging from 0 to 1). To calculate the transition probability from cell state *A* at the earlier timepoint to cell state *B* at the later timepoint, we summed the transition probabilities of all cells within *A* to all cells within *B*, followed by normalizing the total cell number of *B*. Each heatmap shows the transition probabilities from cell states at the earlier timepoint (rows) to cell states at the later timepoint (columns) for each pair of adjacent timepoints, normalized to total probabilities per row (*i.e.* each row sums to one). These transition probabilities, after normalizing per row, are also summarized in **Supplementary Table 5**. Of note, we did not perform RNA velocity analysis for cell states before E6.5 (because of limited numbers of cells and heterogeneous datasets within some of the earlier timepoints; only cells from Pijuan-Sala *et al.* were retained for E6.5) nor for the E8.5a-E8.5b transition (because of the major technical differences). For cell states from E8.5b onward, we performed a random downsampling on each cell state to 1,500 cells prior to RNA velocity analysis, in order to reduce computational costs.

Supplementary Fig. 11 | RNA velocity-based inference of potential cell state relationships across pairs of adjacent timepoints (E8.25 - E13.5). Briefly, we integrated cells from each pair of adjacent timepoints, and then applied RNA velocity analysis using *scVelo*[8]. The resulting transition probabilities between individual cells (stored in a *velocity_graph* matrix), were calculated using cosine correlation

between the potential cell-to-cell transitions and the inferred velocity vector (ranging from 0 to 1). To calculate the transition probability from cell state *A* at the earlier timepoint to cell state *B* at the later timepoint, we summed the transition probabilities of all cells within *A* to all cells within *B*, followed by normalizing the total cell number of *B*. Each heatmap shows the transition probabilities from cell states at the earlier timepoint (rows) to cell states at the later timepoint (columns) for each pair of adjacent timepoints, normalized to total probabilities per row (*i.e.* each row sums to one). These transition probabilities, after normalizing per row, are also summarized in **Supplementary Table 5**. Of note, we did not perform RNA velocity analysis for cell states before E6.5 (because of limited numbers of cells and heterogeneous datasets within some of the earlier timepoints; only cells from Pijuan-Sala *et al.* were retained for E6.5) nor for the E8.5a-E8.5b transition (because of the major technical differences). For cell states from E8.5b onward, we performed a random downsampling on each cell state to 1,500 cells prior to RNA velocity analysis, in order to reduce computational costs. PNS: peripheral nervous system. MHB: midbrain-hindbrain boundary. Di: Diencephalon.

Supplementary Fig. 12 | Inferring continuous molecular histories of individual cell types. a, Pseudotime trajectory analysis of pseudobulk RNA-seq profiles of mouse embryos. Briefly, epiblast-derived cells from individual embryos (or pools of embryos comprising each sample, in the case of (Pijuan-Sala *et al.*)[1]) were aggregated to create 111 pseudobulk samples, on which we performed pseudotime trajectory analysis. Each point in the resulting 2D embedding corresponds to an embryo, and the curve to pseudotime trajectory. **b,** Pseudotime of embryos from staged timepoints between E6.25 and E13.5 (*n* = 111 pseudobulk samples). The center lines show the medians; the box limits indicate the 25th and 75th percentiles; the whiskers extend to the 5th and 95th percentiles; the individual samples are represented by the dots. **c,** Pseudotime of male and female embryos from staged timepoints between E8.5b to E13.5 (*n* = 73 pseudobulk samples). The center lines show the medians; the box limits indicate the 25th and 75th percentiles; the whiskers extend to the 5th and 95th percentiles; the individual samples are represented by the dots. For sex separation of embryos, we counted reads mapping to a female-specific non-coding RNA (*Xist*) or chrY genes (except *Erdr1* which is in both chrX and chrY). Embryos were readily separated into females (more reads mapping to Xist than chrY genes) and males (more reads mapping to chrY genes than *Xist*). **d,** Pseudotime of individual embryos with different somite counts from E8.5b. **e,** Smoothed expression profiles for four selected genes for each of four selected cell types (rows; one from each germ layer), along their inferred trajectories (key at left). We selected linear paths corresponding to strongest pseudo-ancestor edges, working back from each E13.5 cell state to the E6.25 epiblast cell state. The first and second columns of plots correspond to key regulators or marker genes, and the third and fourth columns to the genes most positively and negatively correlated with pseudotime, respectively. Each plotted point corresponds to gene expression within a cell state for an individual embryo. Pseudotime values (*x*-axes) as in panel b. The error bands indicate the 95% confidence intervals (CIs) for predictions from a linear model (*stat_smooth* function in R). Although the distributions of estimated pseudotime for E8.5a and E8.5b samples are overlapping, the vertical red lines approximately mark this transition; specifically, it marks the pseudotime of the oldest embryo from (Pijuan-Sala *et al.*)[1]. Gene expression (*y*-axes) calculated as aggregated UMI within a cell state normalized to total UMI per individual, followed by natural-log transformation. The inferred trajectory for the neural crest (PNS glia) spanned epiblast (E6.25 → E7.25), rostral neuroectoderm (E7.5 → E8.0), neural crest (E8.25 → E8.5b) and neural crest (PNS glia) (E9.5 → E13.5). The inferred trajectory for the otic epithelium spanned epiblast (E6.25), primitive streak and adjacent ectoderm (E6.5 → E7.25), surface ectoderm (E7.5 → E8.25),

placodal area (E8.5a & E8.5b), and otic epithelium (E9.5 → E13.5). The inferred trajectory for the midgut/hindgut epithelium spanned epiblast (E6.25), primitive streak and adjacent ectoderm (E6.5 → E6.75), anterior primitive streak (E7), definitive endoderm (E7.25), gut (E7.25 → E8.5b), gut and lung epithelium (E9.5 → E10.5), and midgut/hindgut epithelium (E11.5 → E13.5). The inferred trajectory for the cardiomyocytes spanned epiblast (E6.25), primitive streak and adjacent ectoderm (E6.5), nascent mesoderm (E6.75 → E7.25), splanchnic mesoderm (E7.5), first heart field (E7.75 → E9.5), and cardiomyocytes (E10.5 → E13.5). PNS: peripheral nervous system.

Supplementary Fig. 13 | Gene dynamics across the inferred molecular trajectories of four selected cell types (neural crest (PNS glia) and otic epithelium). a, 155 genes were identified as significantly associated with pseudotime of the neural crest (PNS glia) trajectory, based on linear regression with the origin of the data as a covariate. Genes with bonferroni-adjusted p-value (on the variable of pseudotime) < 0.05 and absolute value of beta coefficient (on the variable of pseudotime) > the mean + 2 standard deviations of beta coefficient were nominated as genes which are correlated to pseudotime. Similarly, genes with bonferroni-adjusted p-value (on the variable of data source) < 0.05 and absolute value of beta coefficient (on the variable of data source) > the mean + 2 standard deviations of beta coefficient were nominated as genes which are correlated to data source. Finally, genes which were nominated as correlated to pseudotime but not correlated to the data source were retained and hierarchically clustered (*y*-axis of heatmap). The columns of the heatmap correspond to different embryos/samples, ordered by pseudotime (below) as shown in **Supplementary Fig. 12a-b**. Although the distributions of estimated pseudotime for E8.5a and E8.5b samples are overlapping, the vertical red lines approximately mark this transition; specifically, it marks the pseudotime of the oldest embryo from (Pijuan-Sala *et al.*)[1]. **b,** 122 genes were identified as significantly associated with pseudotime of the otic epithelium trajectory. Axes as well as thresholds for identifying genes and modules in panels b as in panel a. The inferred trajectory of each cell type included the same cell states as described in **Supplementary Fig. 12e**, in each case starting from epiblast. PNS: peripheral nervous system.

Supplementary Fig. 14 | Gene dynamics across the inferred molecular trajectories of four selected cell types (midgut/hindgut epithelium and cardiomyocyte). a, 124 genes were identified as significantly associated with pseudotime of the midgut/hindgut epithelium trajectory, based on linear regression with the origin of the data as a covariate. Genes with bonferroni-adjusted p-value (on the variable of pseudotime) < 0.05 and absolute value of beta coefficient (on the variable of pseudotime) > the mean + 2 standard deviations of beta coefficient were nominated as genes which are correlated to pseudotime. Similarly, genes with bonferroni-adjusted p-value (on the variable of data source) < 0.05 and absolute value of beta coefficient (on the variable of data source) > the mean + 2 standard deviations of beta coefficient were nominated as genes which are correlated to data source. Finally, genes which were nominated as correlated to pseudotime but not correlated to the data source were retained and hierarchically clustered (*y*-axis of heatmap). The columns of the heatmap correspond to different embryos/samples, ordered by pseudotime (below) as shown in **Supplementary Fig. 12a-b**. Although the distributions of estimated pseudotime for E8.5a and E8.5b samples are overlapping, the vertical red lines approximately mark this transition; specifically, it marks the pseudotime of the oldest embryo from (Pijuan-Sala *et al.*)[1]. **b,** 85 genes were identified as significantly associated with pseudotime of the cardiomyocyte trajectory. Axes as well as thresholds for identifying genes and modules in panels b as in

panel a. The inferred trajectory of each cell type included the same cell states as described in **Supplementary Fig. 12e**, in each case starting from epiblast.

Supplementary Fig. 15 | Recurrence of individual TFs or genes as candidate upregulated or downregulated key TFs or genes for mouse cell type specification. a, TFs that are most often nominated as downregulated key TFs, e.g. *Pou5f1* (*Oct4*) are identified with red labels, while those most often nominated as upregulated key TFs, e.g. *Klf7*, are identified with green labels. Candidate key TFs frequently recurring in both sets are identified with black labels. The size of each dot corresponds to the number of TFs represented by it on a log2 scale. **b,** Genes that are most often nominated as downregulated key genes, *e.g. Fubp1*, are identified with red labels, while those most often nominated as upregulated key genes, *e.g. Srgap1*, are identified with green labels. Candidate key genes frequently recurring in both sets are identified with black labels. The size of each dot corresponds to the number of TFs represented by it on a log2 scale. **c,** A file containing repressive tendency scores (RTS) was downloaded from (Shim et al. 2020)[15]. It includes 16,298 mouse genes with RTS which define the association between each gene and broad H3K27me3 domains. As described in (Shim et al. 2020)[15], broad H3K27me3 domains occur mostly over important cell-type-specific regulatory genes (*i.e.* high RTS); in contrast, genes with housekeeping or non-regulatory roles rarely host broad H3K27me3 domains (*i.e.* low RTS). Here the distribution of RTS is compared between 462 upregulated key TFs, 194 downregulated key TFs, 590 non-key TFs, and 15,052 non-TF genes. Unadjusted p-values were calculated by two-sided Wilcoxon rank-sum test and are shown above the plots (unadjusted p-values < 0.05 are highlighted in red). Of note, the two groups of upregulated key TFs and downregulated key TFs are mutually exclusive in this comparison, as each key TF was simply categorized as upregulated or downregulated based on the role in which it is nominated the most (TFs that appeared equally often as upregulated and downregulated were put in the upregulated group for this comparison).

References

1. Pijuan-Sala, B. *et al.* A single-cell molecular map of mouse gastrulation and early organogenesis. *Nature* **566**, 490–495 (2019).

2. Martin, B. K. *et al.* An optimized protocol for single cell transcriptional profiling by combinatorial indexing. *arXiv* (2021).

3. Cao, J. *et al.* The single-cell transcriptional landscape of mammalian organogenesis. *Nature* **566**, 496–502 (2019).

4. Stuart, T. *et al.* Comprehensive Integration of Single-Cell Data. *Cell* **177**, 1888–1902.e21 (2019).

5. Mohammed, H. *et al.* Single-Cell Landscape of Transcriptional Heterogeneity and Cell Fate Decisions during Mouse Early Gastrulation. *Cell Rep.* **20**, 1215–1228 (2017).

6. Wagner, D. E. & Klein, A. M. Lineage tracing meets single-cell omics: opportunities and challenges. *Nat. Rev. Genet.* **21**, 410–427 (2020).

7. La Manno, G. *et al.* RNA velocity of single cells. *Nature* **560**, 494–498 (2018).

8. Bergen, V., Lange, M., Peidli, S., Wolf, F. A. & Theis, F. J. Generalizing RNA velocity to transient cell states through dynamical modeling. *Nat. Biotechnol.* **38**, 1408–1414 (2020).

9. Sagner, A. *et al.* Temporal patterning of the central nervous system by a shared transcription factor code. doi:10.1101/2020.11.10.376491.

10. Wagner, D. E. *et al.* Single-cell mapping of gene expression landscapes and lineage in the zebrafish embryo. *Science* **360**, 981–987 (2018).

11. Farrell, J. A. *et al.* Single-cell reconstruction of developmental trajectories during zebrafish embryogenesis. *Science* **360**, (2018).

12. Briggs, J. A. *et al.* The dynamics of gene expression in vertebrate embryogenesis at single-cell resolution. *Science* **360**, (2018).

13. Fukazawa, C. *et al.* poky/chuk/ikk1 is required for differentiation of the zebrafish embryonic epidermis. *Dev. Biol.* **346**, 272–283 (2010).

14. Parker, H. J., Pushel, I. & Krumlauf, R. Coupling the roles of Hox genes to regulatory networks patterning cranial neural crest. *Dev. Biol.* **444 Suppl 1**, S67–S78 (2018).

15. Shim, W. J. *et al.* Conserved Epigenetic Regulatory Logic Infers Genes Governing Cell Identity. *Cell Syst* **11**, 625–639.e13 (2020).

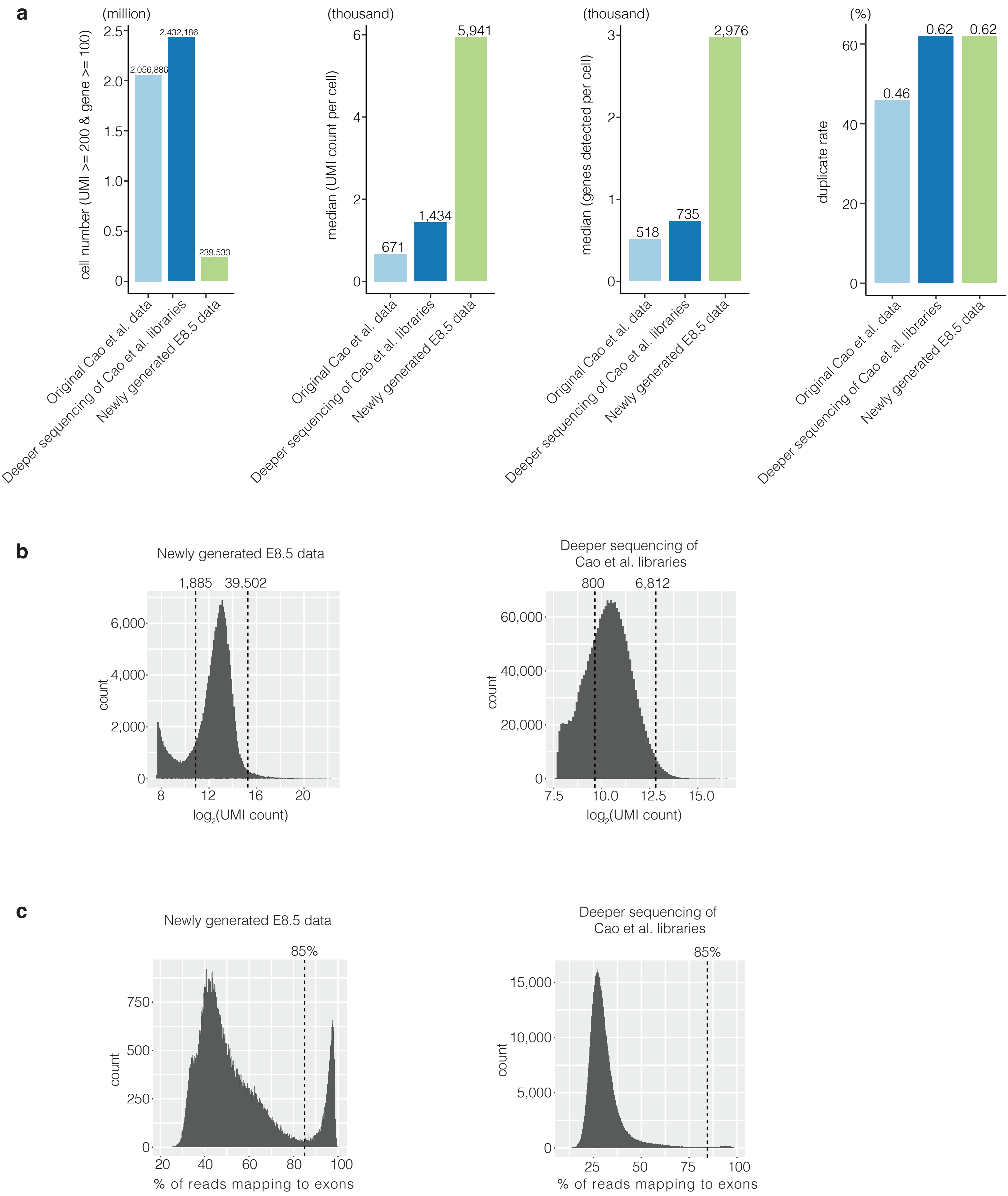

Supplementary Fig. 1 l Higher quality sci-RNA-seq3 data generated by either application of an optimized protocol (E8.5b) or deeper sequencing of previously reported libraries (E9.5 - E13.5).

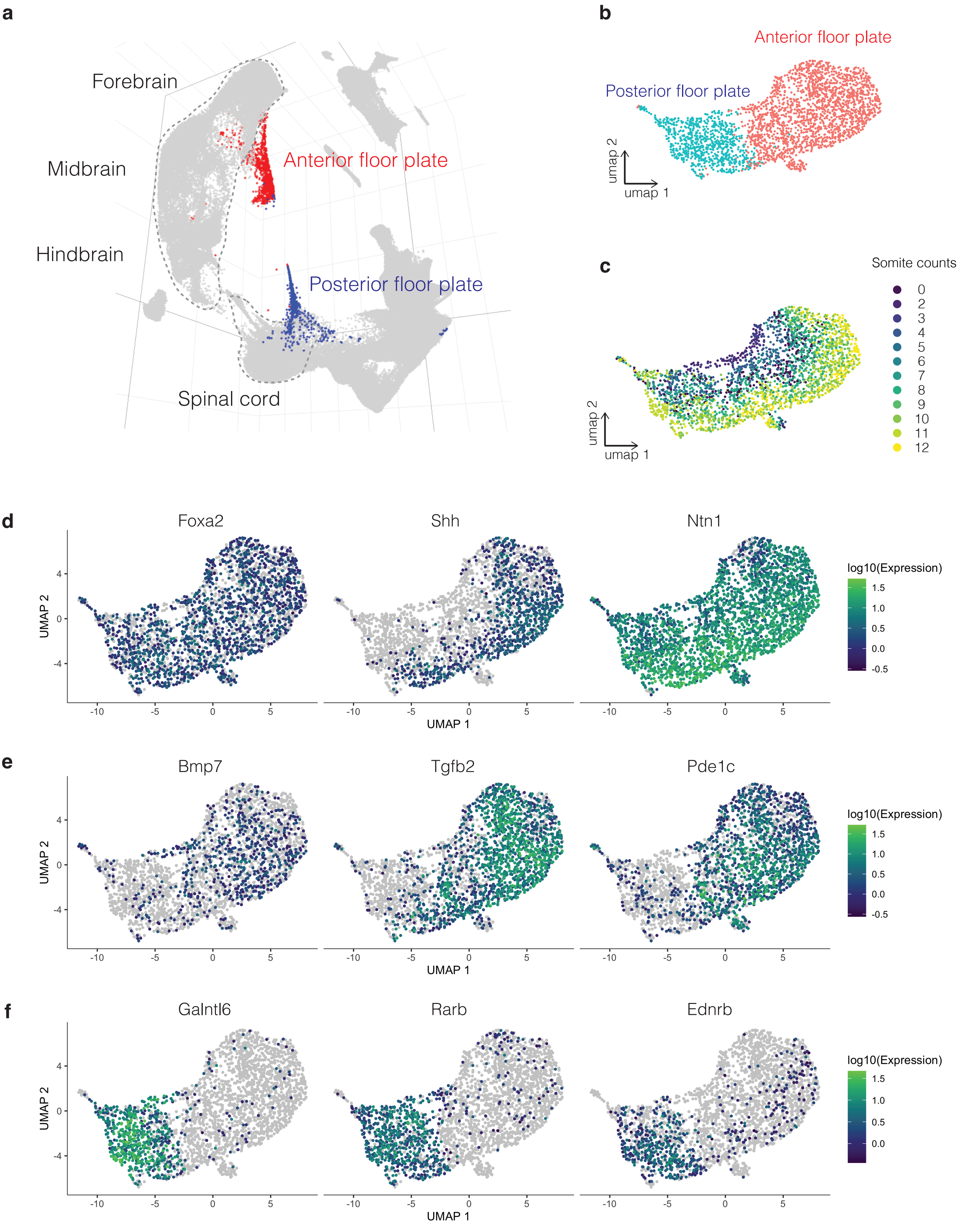

Supplementary Fig. 2 | Anterior and posterior floor plate subpopulations emerging from forebrain/hindbrain and spinal cord, respectively.

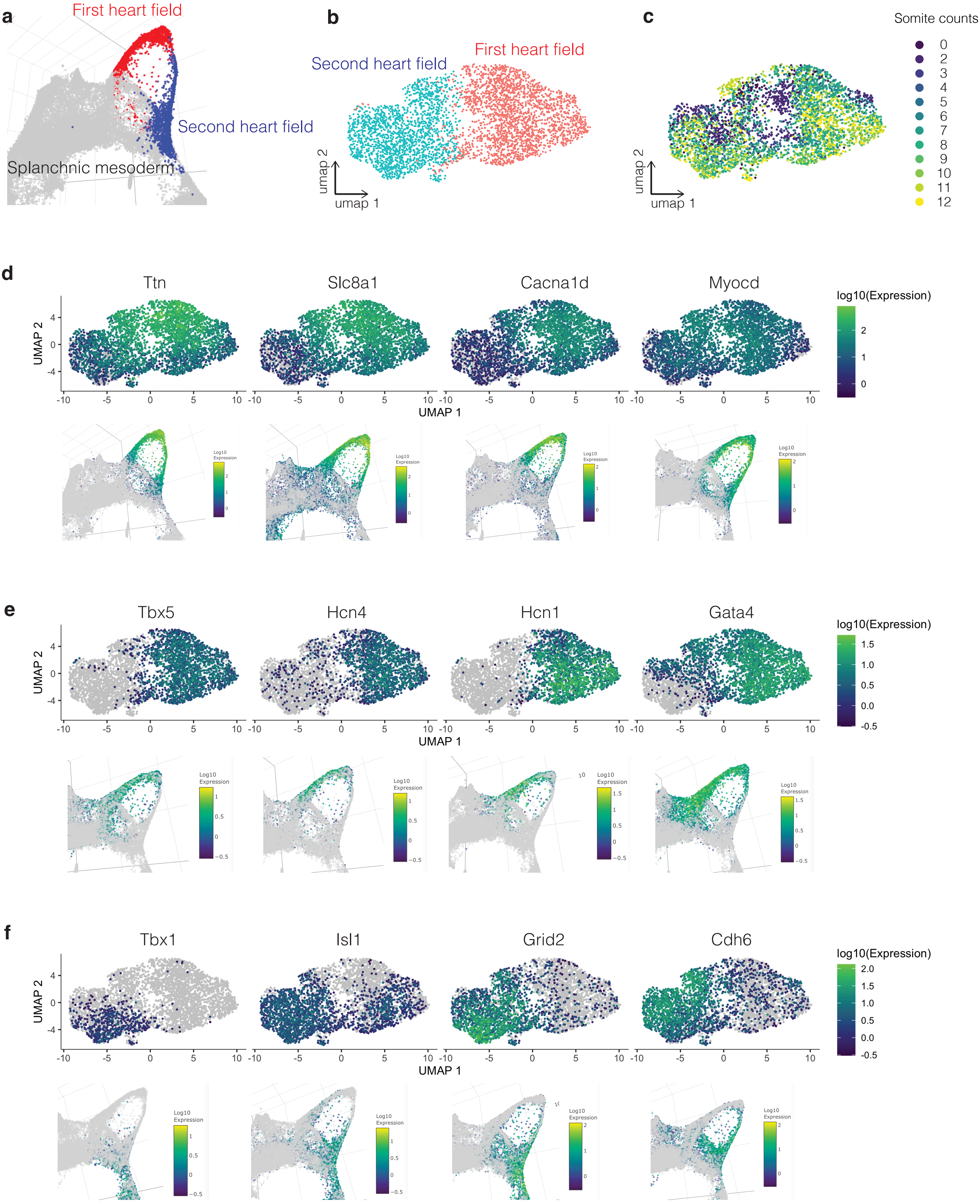

Supplementary Fig. 3 | Resolution of the first and second heart fields during early somitogenesis.

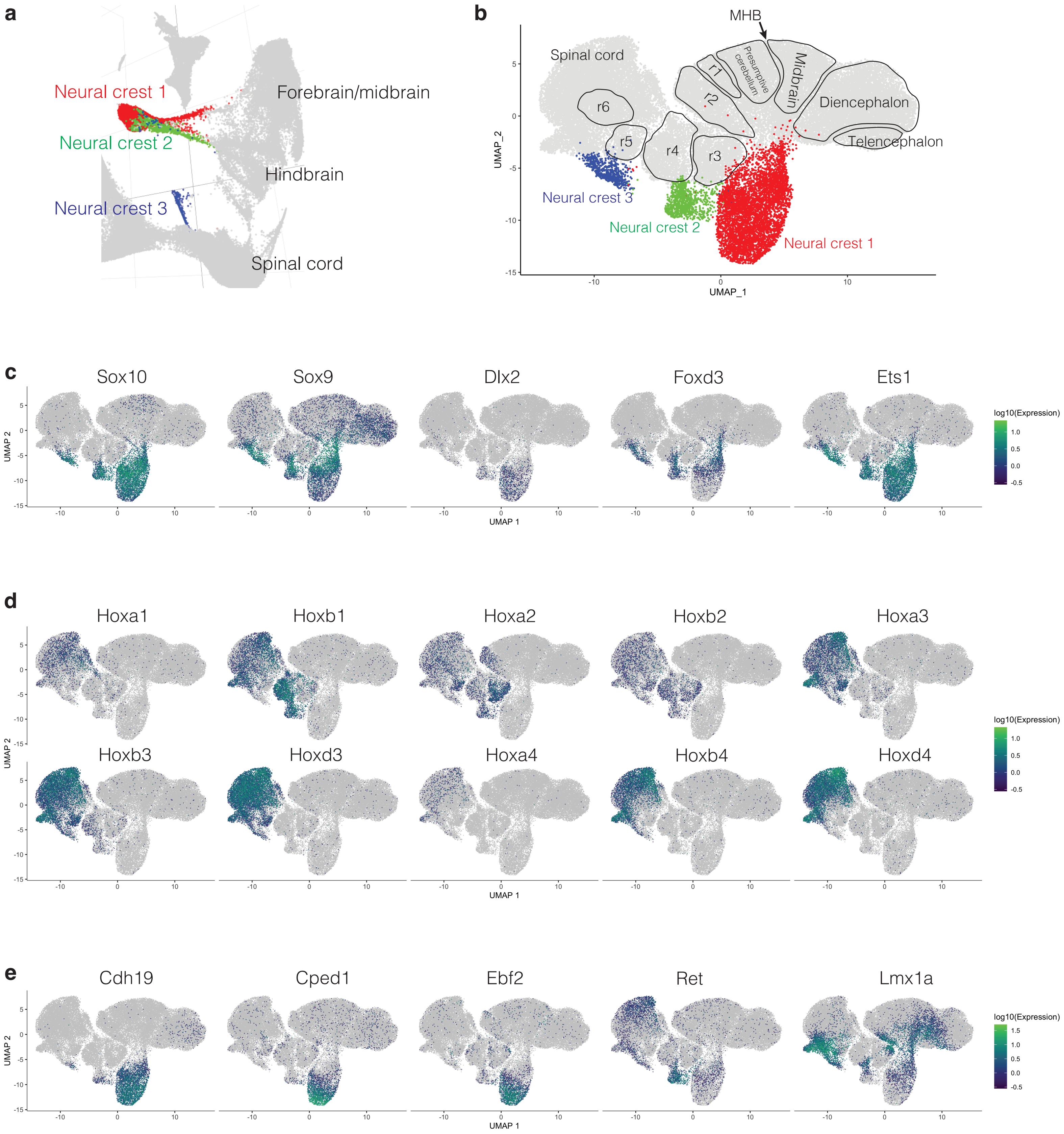

Supplementary Fig. 4 | Three distinct subpopulations of neural crest cells (NCCs) may correspond to mesencephalic NCCs and pharyngeal arch (PA) contributions.

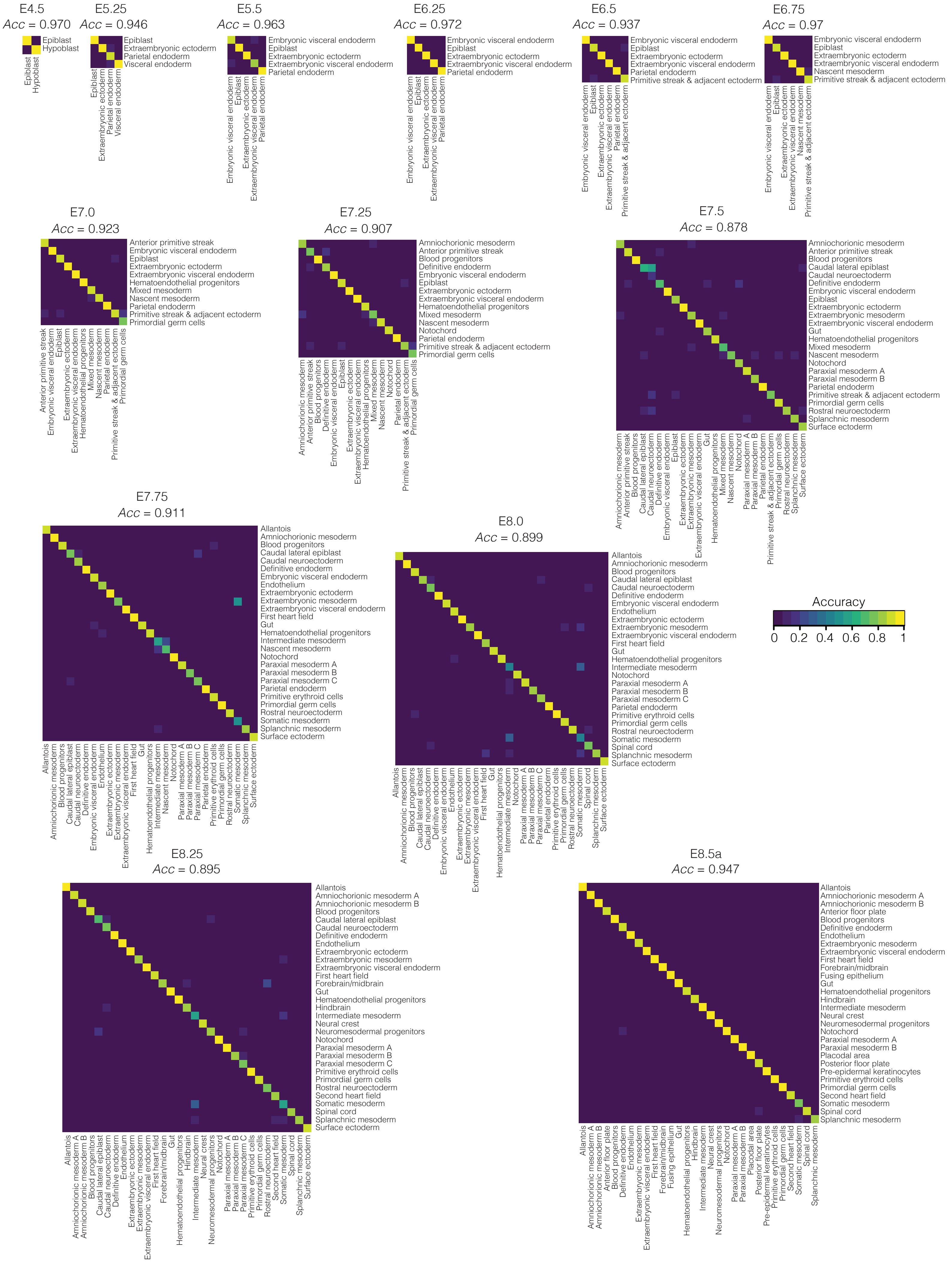

Supplementary Fig. 5 | Benchmarking of the robustness of cell type annotations (E4.5 - E8.5a).

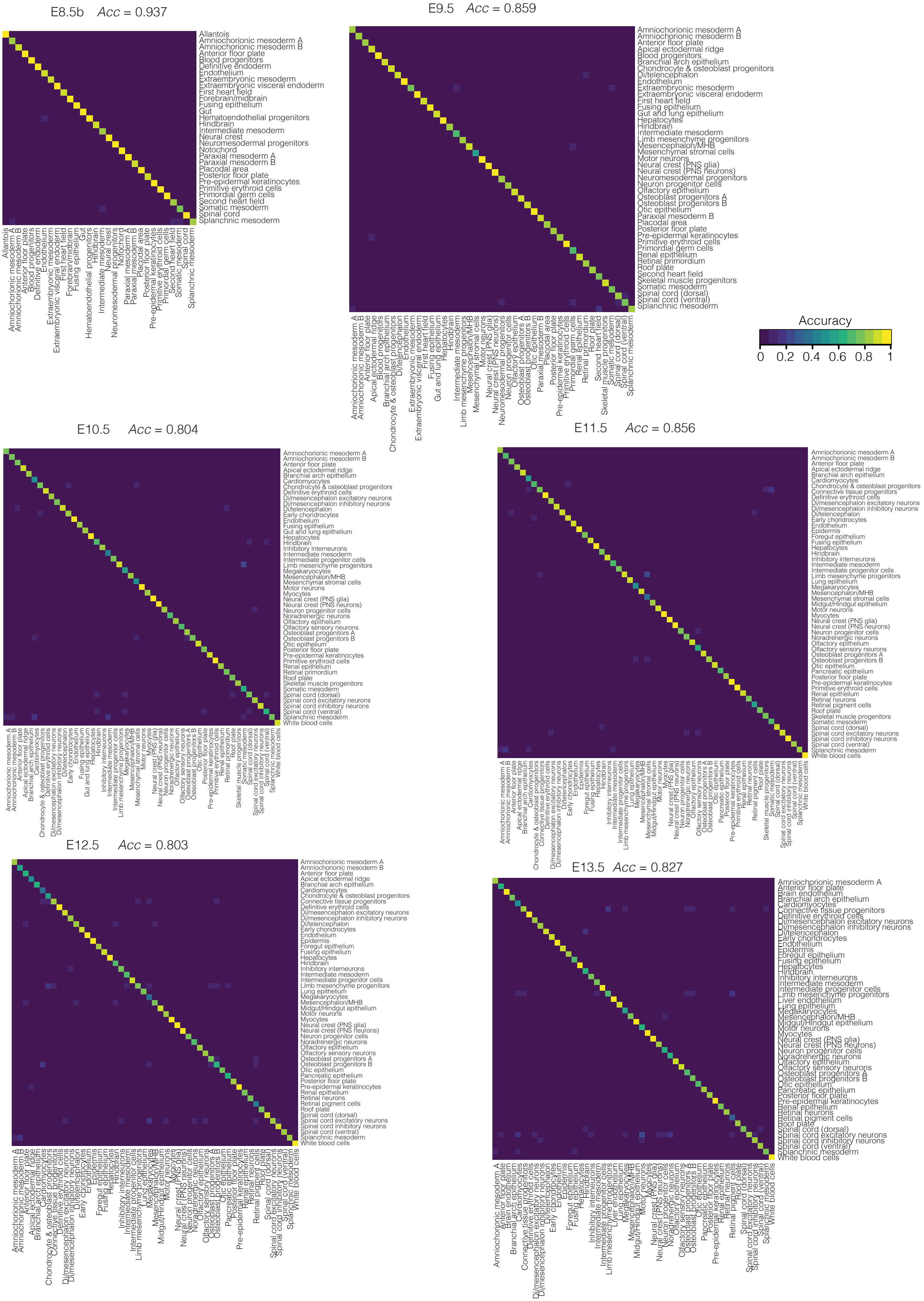

Supplementary Fig. 6 | Benchmarking of the robustness of cell type annotations (E8.5b - E13.5).

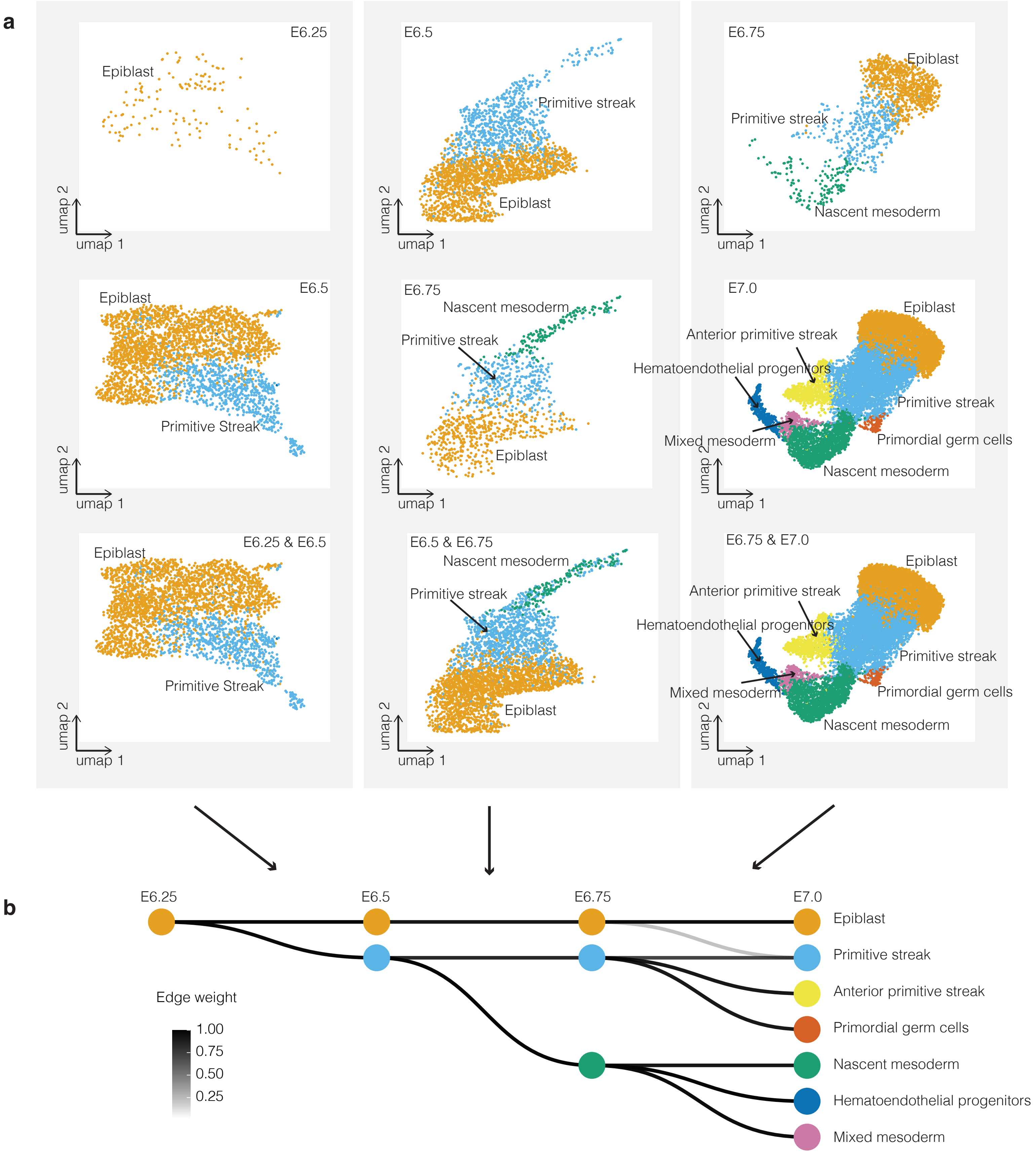

Supplementary Fig. 7 | Inference of epiblast derivatives between E6.25 and E7.0.

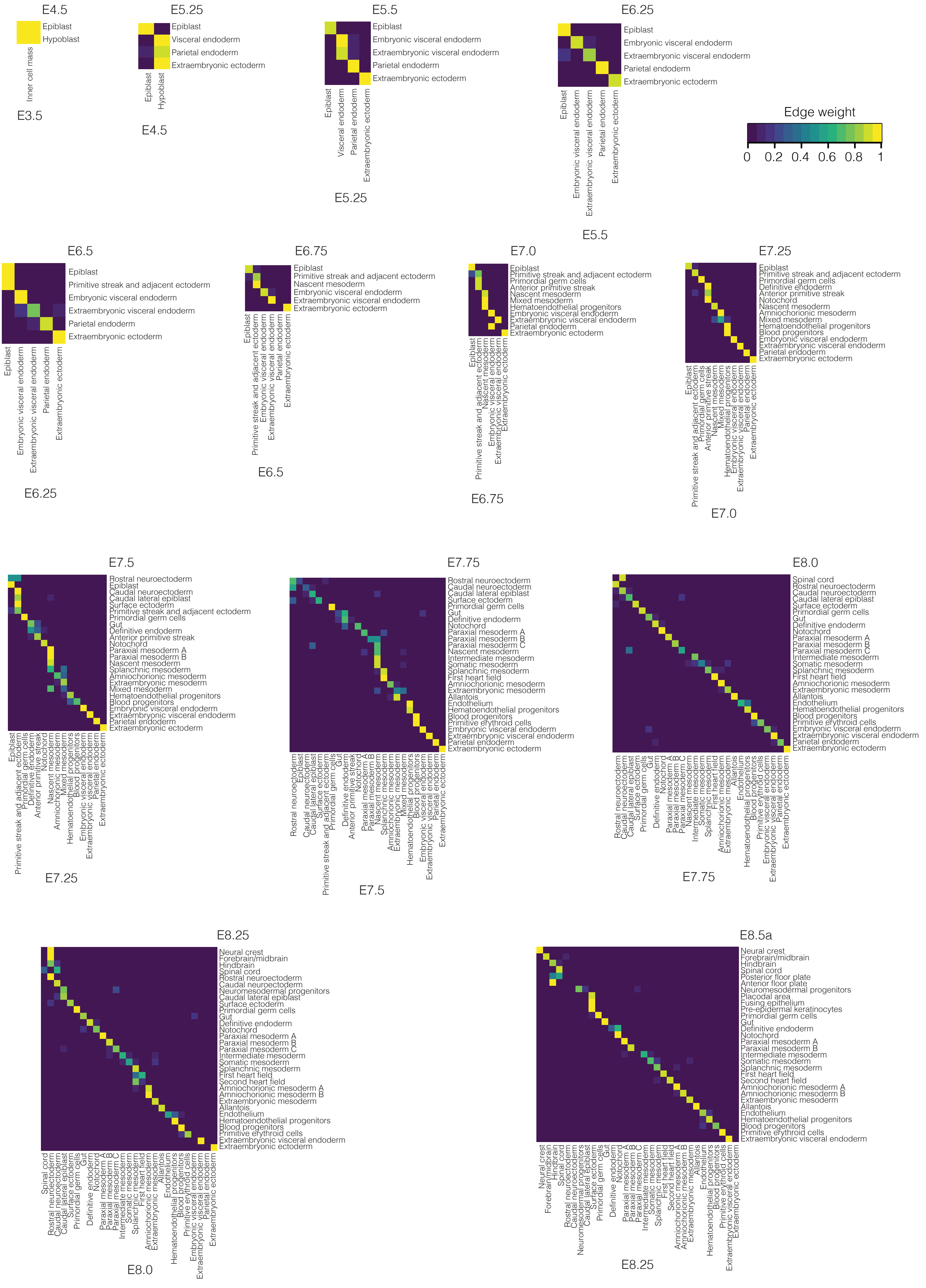

Supplementary Fig. 8 | Heatmap of edge weights between cell states at each pair of adjacent timepoints (E3.5 - E8.5a).

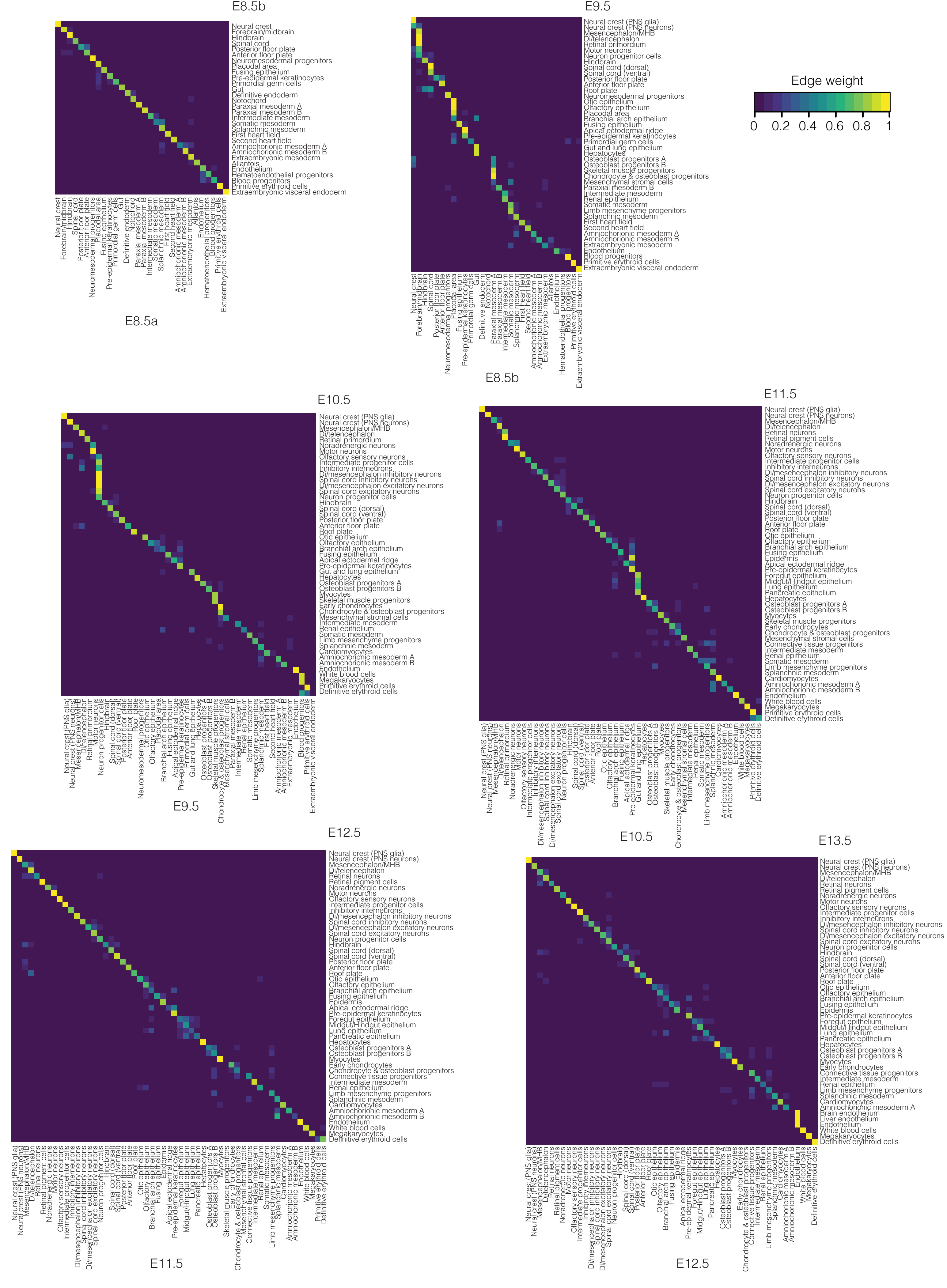

Supplementary Fig. 9 | Heatmap of edge weights between cell states at each pair of adjacent timepoints (E8.5a - E13.5).

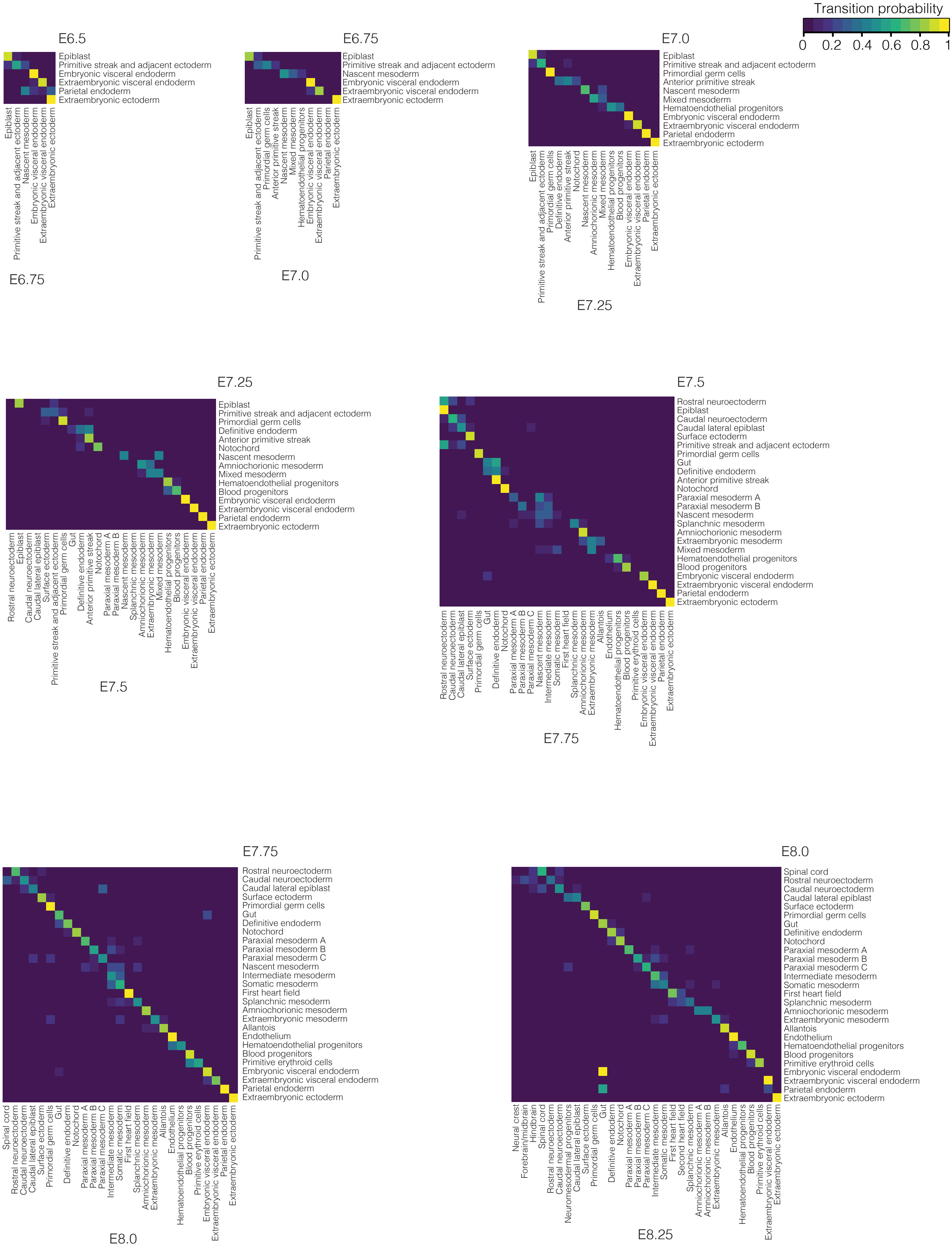

Supplementary Fig. 10 | RNA velocity-based inference of potential cell state relationships across pairs of adjacent timepoints (E6.5 - E8.25).

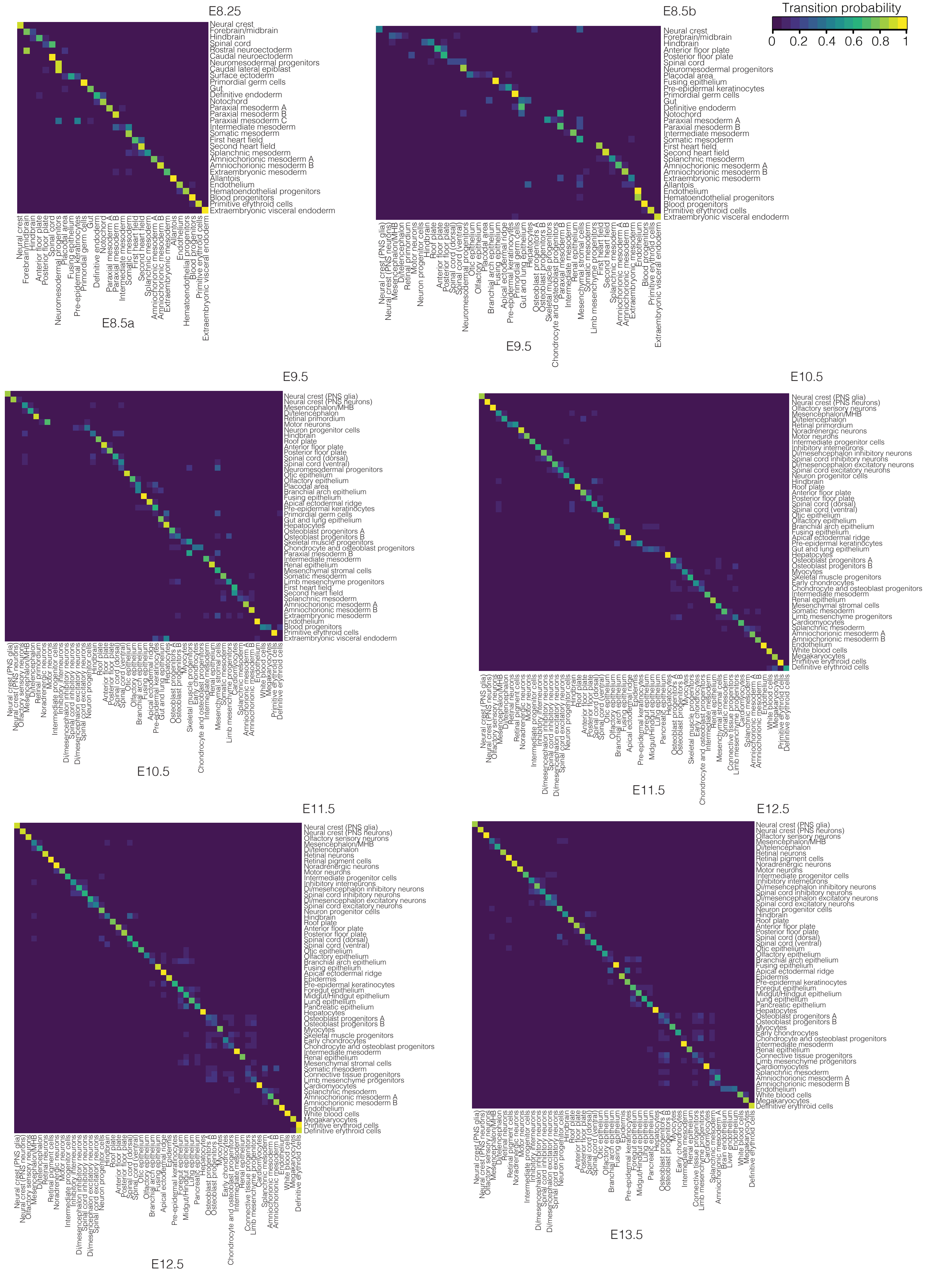

Supplementary Fig. 11 | RNA velocity-based inference of potential cell state relationships across pairs of adjacent timepoints (E8.25 - E13.5).

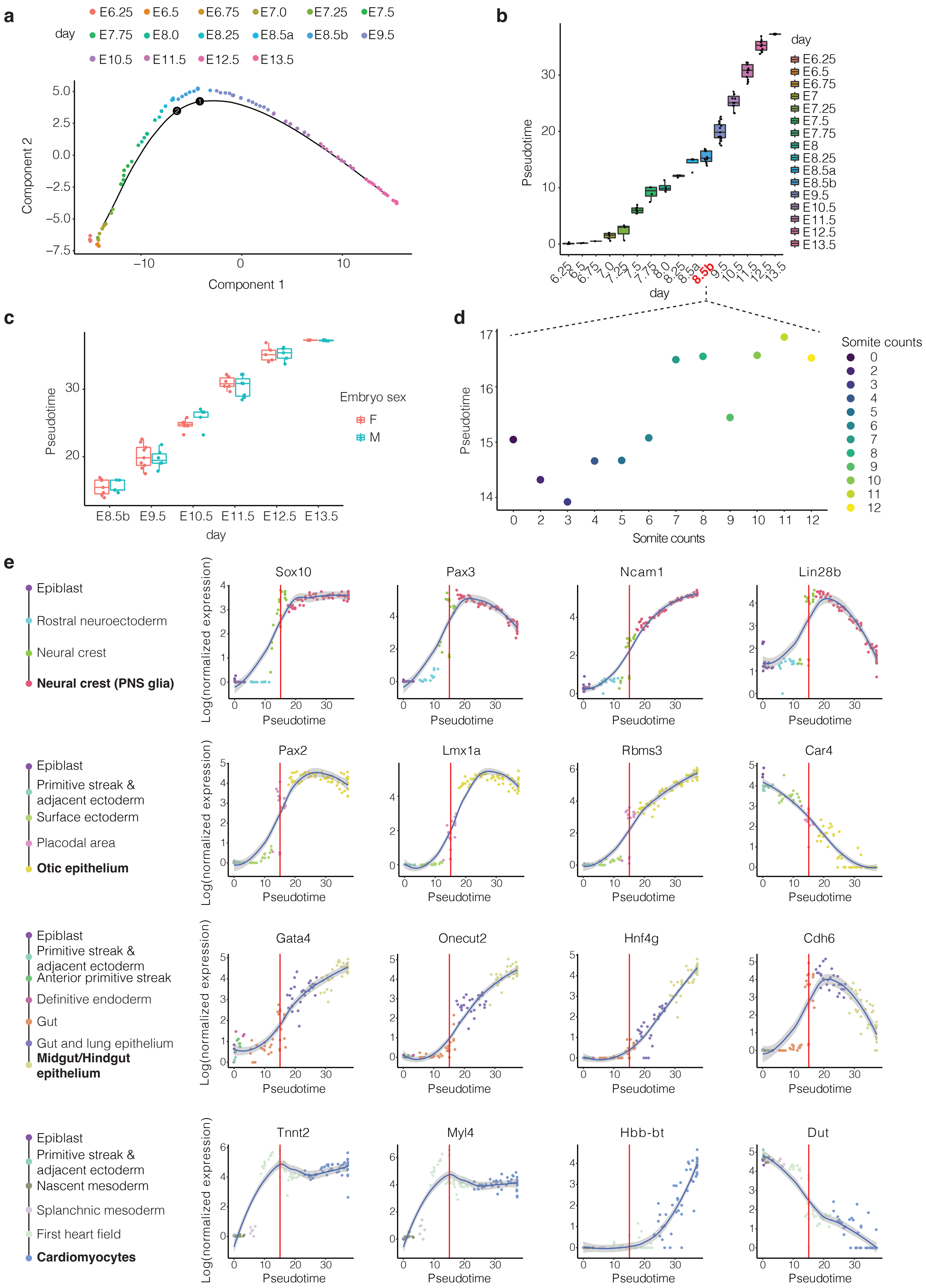

Supplementary Fig. 12 | Inferring continuous molecular histories of individual cell types.

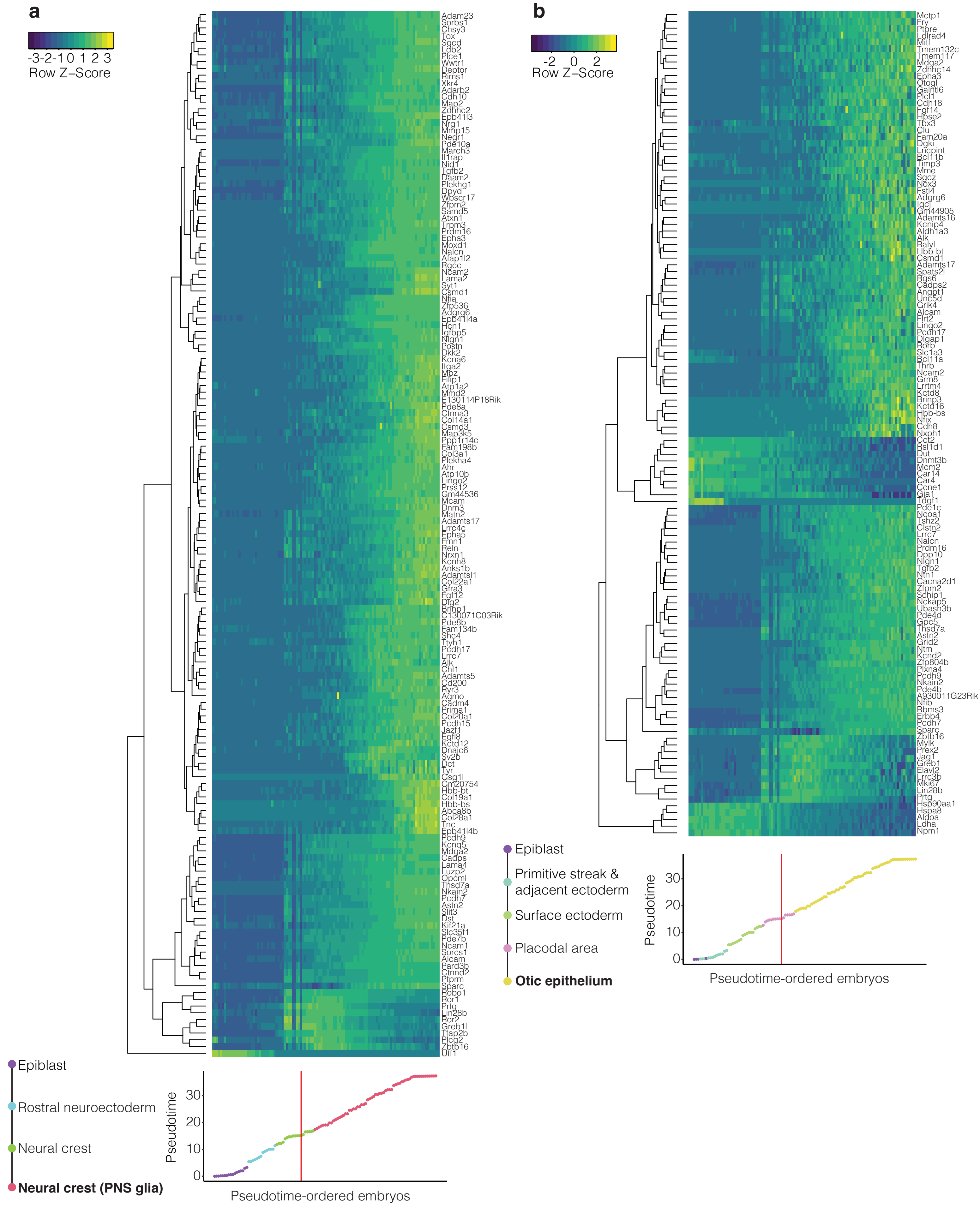

Supplementary Fig. 13 | Gene dynamics across the inferred molecular trajectories of four selected cell types (neural crest (PNS glia) and otic epithelium).

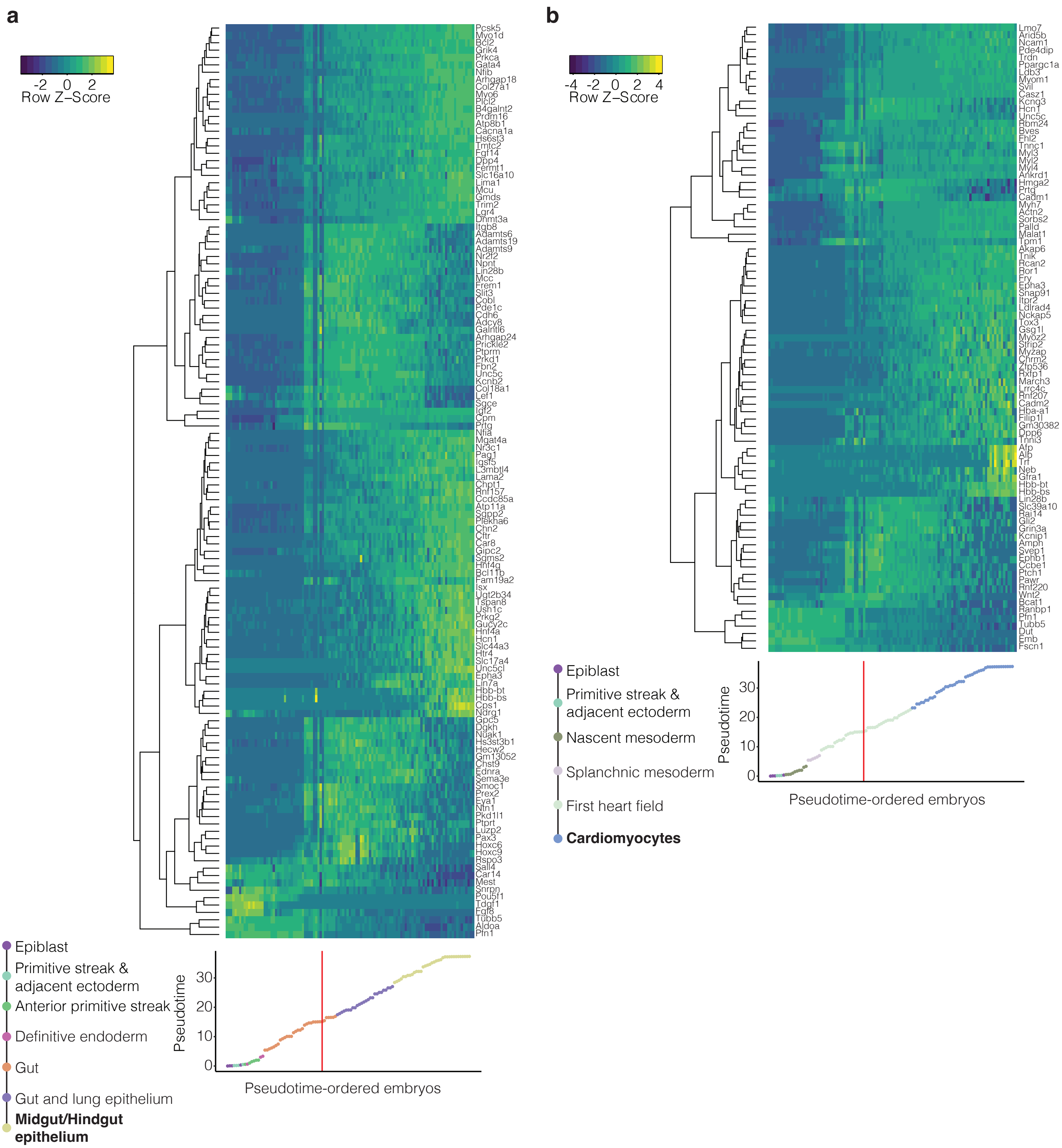

Supplementary Fig. 14 I Gene dynamics across the inferred molecular trajectories of four selected cell types (midgut/hindgut epithelium and cardiomyocyte).

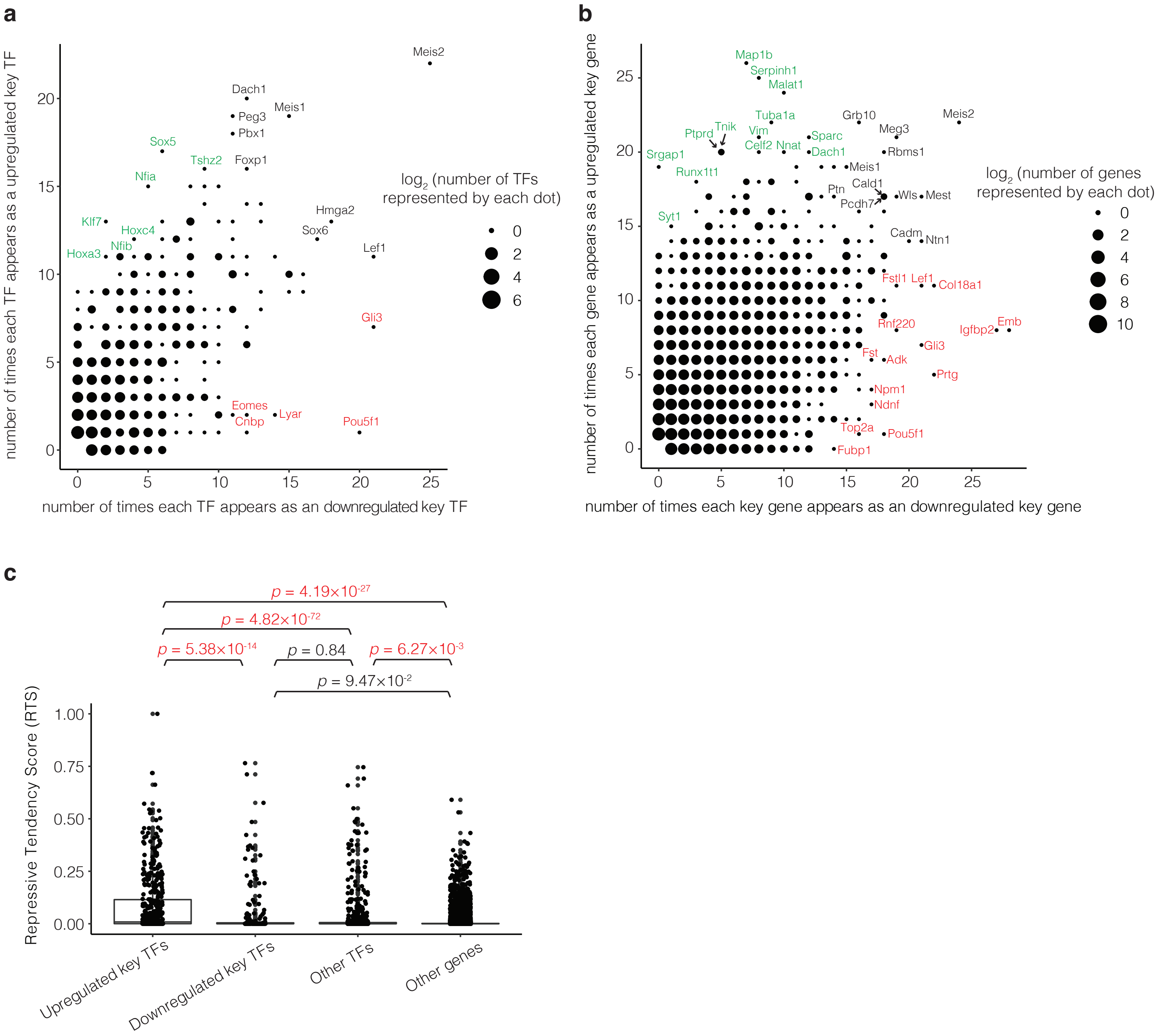

Supplementary Fig. 15 | Recurrence of individual TFs or genes as candidate upregulated or downregulated key TFs or genes for mouse cell type specification.
