# Supplementary material for: Systematic reconstruction of cellular trajectories across mouse embryogenesis
Source: Nat Genet. 2022 Mar 14;54(3):328–41. doi: 10.1038/s41588-022-01018-x (PMC8920898; doi:10.1038/s41588-022-01018-x)
Supplement: Supplementary file 2 — Reporting Summary. [file 41588_2022_1018_MOESM2_ESM.pdf]

nature research

Corresponding author(s): Jay Shendure

Last updated by author(s): Dec 23, 2021

Reporting Summary

Nature Research wishes to improve the reproducibility of the work that we publish. This form provides structure for consistency and transparency in reporting. For further information on Nature Research policies, see our Editorial Policies and the Editorial Policy Checklist.

Statistics

For all statistical analyses, confirm that the following items are present in the figure legend, table legend, main text, or Methods section.

n/a	Confirmed	
☐	☒	The exact sample size (*n*) for each experimental group/condition, given as a discrete number and unit of measurement
☐	☒	A statement on whether measurements were taken from distinct samples or whether the same sample was measured repeatedly
☐	☒	The statistical test(s) used AND whether they are one- or two-sided<br>*Only common tests should be described solely by name; describe more complex techniques in the Methods section.*
☐	☒	A description of all covariates tested
☐	☒	A description of any assumptions or corrections, such as tests of normality and adjustment for multiple comparisons
☐	☒	A full description of the statistical parameters including central tendency (e.g. means) or other basic estimates (e.g. regression coefficient) AND variation (e.g. standard deviation) or associated estimates of uncertainty (e.g. confidence intervals)
☐	☒	For null hypothesis testing, the test statistic (e.g. *F*, *t*, *r*) with confidence intervals, effect sizes, degrees of freedom and *P* value noted<br>*Give P values as exact values whenever suitable.*
☒	☐	For Bayesian analysis, information on the choice of priors and Markov chain Monte Carlo settings
☒	☐	For hierarchical and complex designs, identification of the appropriate level for tests and full reporting of outcomes
☐	☒	Estimates of effect sizes (e.g. Cohen's *d*, Pearson's *r*), indicating how they were calculated

Our web collection on statistics for biologists contains articles on many of the points above.

Software and code

Policy information about availability of computer code

Data collection	No software was used except Illumina RTA basecalling at this stage.
Data analysis	The Python and R codes used to analyze the RNA-seq data are available at https://github.com/ChengxiangQiu/tome_code.<br>The following common, freely available data analysis software were used to analyze data: bcl2fastq version 2.20 (https://support.illumina.com), deML version 1.1.3 (https://github.com/grenaud/deML), HTseq version 0.6.1 (https://github.com/htseq/htseq), trim_galore version 0.6.5 (https://github.com/FelixKrueger/TrimGalore), STAR version 2.6.1d (https://github.com/alexdobin/STAR), scrublet version 0.1 (https://github.com/swolock/scrublet), Scanpy version 1.6.0 (https://github.com/theislab/scanpy), Monocle version 2, 3, and 3-alpha (https://cole-trapnell-lab.github.io/monocle3), DDRTree version 0.1.5 (https://github.com/cole-trapnell-lab/DDRTree), Seurat version 3 (https://github.com/satijalab/seurat), scikit-learn version 1.0 (https://github.com/scikit-learn/scikit-learn), kb-python version 0.25.0 (https://github.com/pachterlab/kb_python), velocyto version 0.6 (https://github.com/velocyto-team/velocyto.py), CIBERSORTx (https://github.com/ysuzukilab/Cibersortx), HOMER version 4.11 (https://github.com/IGBIllinois/HOMER).

For manuscripts utilizing custom algorithms or software that are central to the research but not yet described in published literature, software must be made available to editors and reviewers. We strongly encourage code deposition in a community repository (e.g. GitHub). See the Nature Research guidelines for submitting code & software for further information.

Data

Policy information about availability of data

All manuscripts must include a data availability statement. This statement should provide the following information, where applicable:

- Accession codes, unique identifiers, or web links for publicly available datasets
- A list of figures that have associated raw data
- A description of any restrictions on data availability

All data have been made freely available via http://tome.gs.washington.edu. The data generated in this study can be downloaded in raw and processed forms from the NCBI Gene Expression Omnibus under accession number GSE186069 (new E8.5 data) & GSE186068 (deeper sequencing of Cao et al. libraries).
The following publicly available datasets were used in this project: AnimalTFDB/v3 (http://bioinfo.life.hust.edu.cn/AnimalTFDB/). The mouse gastrulation dataset generated by Pijuan-Sala et al. (https://github.com/MarioniLab/EmbryoTimecourse2018 and ArrayExpress (E-MTAB-6967)). The mouse pre-gastrulation dataset generated by Mohammed et al. (NCBI GEO (GSE100597)). The mouse pre-gastrulation dataset generated by Cheng et al. (NCBI GEO (GSE109071)). The zebrafish embryogenesis dataset generated by Farrell et al. (NCBI GEO (GSE106587)). The zebrafish embryogenesis dataset generated by Wagner et al. (NCBI GEO (GSE112294)). The Xenopus embryogenesis dataset generated by Briggs et al. (NCBI GEO (GSE113074)).

Field-specific reporting

Please select the one below that is the best fit for your research. If you are not sure, read the appropriate sections before making your selection.

☒ Life sciences ☐ Behavioural & social sciences ☐ Ecological, evolutionary & environmental sciences

For a reference copy of the document with all sections, see nature.com/documents/nr-reporting-summary-flat.pdf

Life sciences study design

All studies must disclose on these points even when the disclosure is negative.

Sample size	For newly generated E8.5b data, no statistical methods were used to predetermine sample size. In the previous single-cell mouse gastrulation study, Pijuan-Sala et al. recovered 16,909 cells from 10 mouse embryos staged at E8.5. The cell types what they identified by sc-RNA-seq data were generally consistent with our current knowledge on the mouse development at that stage. To bridge technologies, our new data, generated via optimized sci-RNA-seq3 at E8.5 (n = 154,313 cells, from 12 mouse embryos), enabled the identification of the same 30 cell types as we identified with E8.5 data from (Pijuan-Sala et al. 2019). Moreover, the depth of the new data, together with the fact that we separately processed individual somite-resolved embryos, facilitated the resolution of substantial substructure (e.g. A-P floor plates, different segmentations of the hindbrain).
Data exclusions	For newly generated E8.5b data and deeper sequencing of the original libraries (Cao et al. 2019), we excluded cells which are potential doublets and low-quality cells by investigating the numbers of UMIs and the proportion of reads mapping to the exonic regions per cell. Except that, no data were excluded from the study.
Replication	We pooled twelve mouse embryos at E8.5 to perform sc-RNA-seq experiments. As a benchmark, the new data enabled the identification of the same 30 cell types as we identified with E8.5 data from (Pijuan-Sala et al. 2019).
Randomization	For newly generated E8.5b data, embryos used in experiments were randomized before sample preparation.
Blinding	For newly generated E8.5b data, investigators were blinded to group allocation during data collection and analysis: embryo collection and sci-RNA-seq3 analysis were performed by different researchers in different locations.

Reporting for specific materials, systems and methods

We require information from authors about some types of materials, experimental systems and methods used in many studies. Here, indicate whether each material, system or method listed is relevant to your study. If you are not sure if a list item applies to your research, read the appropriate section before selecting a response.

Materials & experimental systems

n/a	Involved in the study	
☒	☐	Antibodies
☒	☐	Eukaryotic cell lines
☒	☐	Palaeontology and archaeology
☐	☒	Animals and other organisms
☒	☐	Human research participants
☒	☐	Clinical data
☒	☐	Dual use research of concern

Methods

n/a	Involved in the study	
☒	☐	ChIP-seq
☒	☐	Flow cytometry
☒	☐	MRI-based neuroimaging

Animals and other organisms

Policy information about studies involving animals; ARRIVE guidelines recommended for reporting animal research

Laboratory animals	We collected mouse embryos (C57BL/6, 5 males and 7 females) at E8.5. Mice were housed in a barrier research animal facility that maintained a 12 hours light:12 hours dark light cycle, ambient temperature of 65-75°F (~18-23°C), and 40-60% humidity.
Wild animals	Study did not involve wild animals
Field-collected samples	Study did not involve field-collected samples
Ethics oversight	All animal use at The Jackson Laboratory was done in accordance with the Animal Welfare Act and the AVMA Guidelines on Euthanasia, in compliance with the ILAR Guide for Care and Use of Laboratory Animals, and with prior approval from the animal care and use committee (ACUC) under protocol AUS20028.

Note that full information on the approval of the study protocol must also be provided in the manuscript.
