# Supplementary material for: Systematic reconstruction of cellular trajectories across mouse embryogenesis
Source: Nat Genet. 2022 Mar 14;54(3):328–41. doi: 10.1038/s41588-022-01018-x (PMC8920898; doi:10.1038/s41588-022-01018-x)
Supplement: Supplementary file 3 — Peer Review Information. [file 41588_2022_1018_MOESM3_ESM.pdf]

naturereseach

Peer Review Information

Journal: Nature Genetics
Manuscript Title: Systematic reconstruction of cellular trajectories across mouse embryogenesis
Corresponding author name(s): Professor Jay Shendure

Reviewer Comments & Decisions:

Decision Letter, initial version:

11th May 2021

Dear Jay,

Your Analysis, "Systematic reconstruction of the cellular trajectories of mammalian embryogenesis", has now been seen by 3 referees. I apologize for the long review process. Unfortunately, reviewer #4 (single-cell omics expert) has not submitted a timely report. We have now decided to proceed based on the current feedback.

You will see from the reviewers' comments copied below that while they find your work of potential interest, they have raised quite substantial concerns that must be carefully addressed. In light of these comments, we cannot accept the manuscript for publication, but would be interested in considering a revised version that addresses these serious concerns.

Reviewer #1 is quite positive about this work overall. Their comments are mainly aimed at trying to refine the analysis.
Reviewer #2 thinks there is value in consolidating all these published datasets and that therefore this is a useful resource. However, they note that conceptually this is rather similar to previous analyses. They also highlight that there seems to be only limited new biological insights.
Reviewer #3 raises some potential issues regarding data integration and interpretation, and thinks that the biological findings largely agree with published literature, so there seems to be little novelty here (similar to Reviewer #2's point).
I think it would be very useful to clearly articulate in a revised manuscript what the potentially novel findings are.

We hope you will find the referees' comments useful as you decide how to proceed. If you wish to submit a substantially revised manuscript, please bear in mind that we will be reluctant to approach the referees again in the absence of major revisions.

If you choose to revise your manuscript taking into account all reviewer and editor comments, please highlight all changes in the manuscript text file. At this stage we will need you to upload a copy of the manuscript in MS Word .docx or similar editable format.

We are committed to providing a fair and constructive peer-review process. Do not hesitate to contact me if there are specific requests from the reviewers that you believe are technically impossible or unlikely to yield a meaningful outcome.

If revising your manuscript:

*1) Include a “Response to referees” document detailing, point-by-point, how you addressed each referee comment. If no action was taken to address a point, you must provide a compelling argument. This response will be sent back to the referees along with the revised manuscript.

*2) If you have not done so already please begin to revise your manuscript so that it conforms to our Analysis format instructions, available here.
Refer also to any guidelines provided in this letter.

*3) Include a revised version of any required Reporting Summary:
https://www.nature.com/documents/nr-reporting-summary.pdf
It will be available to referees (and, potentially, statisticians) to aid in their evaluation if the manuscript goes back for peer review.
A revised checklist is essential for re-review of the paper.

Please be aware of our guidelines on digital image standards.

You may use the link below to submit your revised manuscript and related files:

[REDACTED]

Note: This URL links to your confidential home page and associated information about manuscripts you may have submitted, or that you are reviewing for us. If you wish to forward this email to co-authors, please delete the link to your homepage.

If you wish to submit a suitably revised manuscript we would hope to receive it within 6 months. If you cannot send it within this time, please let us know. We will be happy to consider your revision so long as nothing similar has been accepted for publication at Nature Genetics or published elsewhere. Should your manuscript be substantially delayed without notifying us in advance and your article is eventually published, the received date would be that of the revised, not the original, version.

Please do not hesitate to contact me if you have any questions or would like to discuss the required revisions further.

Nature Genetics is committed to improving transparency in authorship. As part of our efforts in this direction, we are now requesting that all authors identified as 'corresponding author' on published papers create and link their Open Researcher and Contributor Identifier (ORCID) with their account on the Manuscript Tracking System (MTS), prior to acceptance. ORCID helps the scientific community achieve unambiguous attribution of all scholarly contributions. You can create and link your ORCID from the home page of the MTS by clicking on 'Modify my Springer Nature account'. For more information please visit please visit www.springernature.com/orcid.

Thank you for the opportunity to review your work.

Sincerely,

Tiago

Tiago Faial, PhD
Senior Editor
Nature Genetics
https://orcid.org/0000-0003-0864-1200

Referee expertise:

Referee #1: transcriptomics, mammalian development

Referee #2: single-cell omics, vertebrate development

Referee #3: mammalian development

Reviewers' Comments:

Reviewer #1:

Remarks to the Author:
Qiu and colleagues integrated independent single-cell datasets covering most mouse embryonic development to infer the relationships between cell states throughout development. The integration results are overall very good, and the inferred cellular and, to a lesser extent, molecular trajectories recapitulate well what is known from mouse development, showcasing the power of the approach. I enjoyed the authors going further and applying their methodology to analogous datasets for zebrafish and Xenopus and then attempting to identify homologous cell types across vertebrates. This is a very challenging problem given the large evolutionary distances involved, and while the results obtained are far from a final answer, they take us one step further in the right direction. I anticipate this work becoming an important resource for the single-cell, development and evolution communities.

Methodologically, this work is strong; state-of-the-art methods/approaches applied throughout. The figures are excellent, complex procedures and results are made intuitive (e.g., Fig. 4d and Supp. Fig. 2 are terrific). For the most part, the authors did an excellent job laying out the goals, assumptions and limitations of their data and analyses. For example, the discussions of the similarities and differences between the molecular trajectories identified for the mouse and the known cellular phylogenies are very good. This was an enjoyable paper to read, and I heartily congratulate the authors on their work.

1. The integration of the independent single-cell datasets is very good, except for the transition between stages e8.5 and e9.5 when single-cell and single-nuclei datasets are combined. These integration problems are seen in several figures (e.g. Supp. Fig. 1e, Supp. Fig. 5) and likely have downstream consequences. This should be explicitly acknowledged at the start of the manuscript when the integration is first described. If this integration issue creates problems for the inference of cellular trajectories, this should also be explicitly stated, especially if it leads to incongruences with the known cellular phylogenies. Is the e8.5-e9.5 switch the main driver for choosing the 0.2 cutoff for the edge weighs (Fig. 1b)?

2. In lines 264-267, the authors say there is anecdotal evidence for key transcription factors showing sharp up-regulation upon a cell type's first appearance, whereas other genes have more "gradual patterns of change", offering as an example the genes on Sup. Fig. 5c columns 2&3. However, these genes are not "other genes", they are the genes most positively and negatively correlated with pseudotime, which selected for the "gradual pattern of change". I do not see support for a difference between transcription factors and other genes from the data presented, and the batch effects between stages e8.5 and e9.5 do not help.

3. The authors use pseudotime for their molecular reconstruction analyses arguing that "individual embryos do not correspond precisely to their intended timepoints". Male and female mouse embryos have been reported to develop at different rates, is there a sex component to these staging differences (embryo sex should be readily identified using Xist and Y-linked genes)?

4. Is there a way to systematically evaluate how well the key transcription factors identified match previous literature? Without this comparison, it is hard to know how well the approach used for identifying key transcription factors candidates worked. For example, could the authors add to Fig. 4d information on whether or not the transcription factors identified were already known to play a role in the specification of that particular cell lineage?

5. It would also be good to have a way to evaluate how well the approach used to identify cis-regulatory motifs involved in cell-type specification worked. The reported agreement between the key transcription factors identified in the previous section and the enriched binding sites seems poor to me. However, I readily admit that setting expectations for the outcome of this type of analysis is subjective. This is not a sticking point for me; I think this type of question requires ATAC-seq data,

but I could see others benefiting from this analysis.

6. I quite enjoyed the author's attempt to identify homologous cell types across mouse, zebrafish and Xenopus, and the three approaches used are very sensible. I was left very curious about how their third approach would work if, instead of using the key transcription factor candidates, the authors used the 345 +/- 246 key genes associated with the emergence of the cell types.

Minor comments:
1. I don't see a good rationale for using lists of transcription factors for the three species that are 1:1 orthologs with the human set. There are transcription factor databases that include all three species (e.g. AnimalTFDB 3.0). Using 1:1 orthologs makes sense for evolutionary comparisons, but for studying development (within species), it makes sense to use all available genes.

2. In the Methods, the authors should be explicit that in their embeddings and evolutionary analyses, they use sets of genes that are 1:1 orthologs between all species (or pairs of species), not just homologs. Don't forget that paralogs are also homologs. The authors are not using all homologs; they are using orthologs in a 1:1 relationship across species.

3. To this reviewer seeing mammalian in the title and then realizing the work is only for mouse is disappointing. There are already papers comparing development across mammals, and these should be the ones using the word 'mammalian' in the title. Obviously, this is not a sticking point; these are just my two cents.

4. In line 69, "delta in time" should be written in plain language.

5. Please add the colour legend to Figs. 5b,c.

Reviewer #2:
Remarks to the Author:
Qiu et al. have provided a comprehensive re-analysis and integration of existing public scRNA-seq datasets comprising key cell states of the developing mouse, frog, and zebrafish embryos. The study relies heavily on a previously established strategy for reconstructing cell state hierarchies from scRNA-seq data, which was also previously applied to compare vertebrate embryo species (Briggs et al. 2018). The primary achievement of the study is the consolidation of these data into a web resource called "TOME" (trajectories of mammalian embryogenesis), which aims to systematically integrate a "continuous, navigable roadmap of the molecular states of the cell types during mouse development." In addition to scRNA-seq datasets, the authors have incorporated spatial transcriptomics datasets (GEO-seq), they have identified series of differentially expressed transcription factor genes (and inferred cis-regulatory relationships), and they have performed a cross-species comparison with frog and fish datasets to identify putative conserved "cell type homologs". The paper is easy to understand, well written, and the methods used are well explained. The authors also clearly articulate the advantages and pitfalls of their approach and analysis. While the approach used here is not particularly novel, the study performs a much-needed consolidation of existing datasets and delivers an attractive resource.

Overall Comments:

1. Neither the data (see Table S1) nor the primary data analysis strategies (see Briggs et al. 2018) are novel to the present study. The TOME resource (http://tome.gs.washington.edu/) is remarkably similar both in concept and presentation style to previously constructed roadmaps of vertebrate

development (see Briggs et al. 2018 & https://kleintools.hms.harvard.edu/tools/currentDatasetsList_xenopus_v2.html). The notable contribution here is that the resulting resource is for mouse.
2. In the TOME graph, developmental relationships between ancestor and descendant nodes and developmental regulatory genes largely confirm expectations but have apparently not generated any new hypotheses (at least, none mentioned by the authors) that were followed up or tested experimentally. Because no major biological discoveries are being reported, the authors could direct their efforts towards strengthening the usefulness of the TOME resource itself. Given that the overall goal of this study is to generate a "systematic integration" of different 'omics datasets and technologies, it was somewhat disappointing that TOME did not integrate the spatial-omics (GEO-seq), inferred cis-regulatory, or RNA-velocity information, which at present are a bit disconnected from the rest of the study. Could this information be directly incorporated into the TOME resource? Given that the authors have argued in Fig 2 that RNA velocity has indeed helped to resolve specific ambiguities, perhaps such information could be incorporated in the final calculation of edge weights of the TOME graph? Such a strategy has indeed been successfully demonstrated for refining edge weights in a cell state graph in other contexts (e.g. PAGA, Wolf et al. 2019) and could be worth considering here.
3. The online TOME resource should include the zebrafish and frog analyses. At present, only mouse is available.
4. The code provided at http://tome.gs.washington.edu/ is insufficient to reconstruct the analyses presented in the paper.

Specific Comments:

1. In general, the complexity and diversity of cell state structures is expected to increase with developmental time. However, the datasets used in this study (Supplementary Fig. 1b) utilize only minimal sampling depths in the later timepoints. The datasets for the final timepoints (E9.5-E13.5) appear to range from ~500 to ~2000 UMI per cell (the true values are a bit masked by the log2 scale of the y-axis). Can the authors comment on the potential limits of this low sampling depth on their ability to distinguish cell types with highly similar transcriptomes?
2. For the TOME analysis, the authors consider 0.2 as a cutoff to filter edges. What is the rationale for using this cutoff? Could the magnitudes of these edge weights be checked for significance, e.g. by repeating the analysis on randomized data?
3. How are "dummy nodes" constructed?
4. Page 7 - Sagner et al., nd?
5. From Fig. 3D, the authors conclude that they see a convergence of visceral and definitive endoderm. By convergence, here do they mean co-localization? If so, there does not seem to be a complete overlap. Can the authors comment on the potential significance and/or confidence of this result?
6. For integration with GEO-Seq, the authors downsample to 50 cells - is this random sampling and are these patterns reproducible with different subsets of cells?
7. Supplementary Fig. 1E – it would be informative to include a version of the UMAP before batch correction.
8. Authors identify targets of SNAIL1 and RFX3. The authors might consider performing an experimental validation of the repressive and/or inductive activity of these TFs towards candidate target genes identified in their analysis.
9. The authors should describe in detail how transcript counts were compared in the multi-species alignments. Were only 1-to-1 gene ortholog relationships considered? If so, how were these orthologous relationships determined? The authors may consider benchmarking their cell type alignments to those of a recent study (SAMAP, Tarashansky 2020, bioRxiv) that also aligns the same zebrafish and frog scRNA-seq datasets.

Reviewer #3:
Remarks to the Author:
This report described a data analytics paradigm that integrates the single-cell transcriptome dataset of cell population from mouse embryos at pre-implantation (E3.5) to stages of organogenesis (E13.5) and collates the molecular trajectory of cell types during mouse embryogenesis, coined as TOME. Through the mining of TOME, the nominal phylogenetic relationship of cell types and the molecular drivers of cell type diversification, in particular the engagement of combinatorial transcription factors in specific steps of the phylogeny can be retrieved from the dataset. Cross analysis of the transcriptome and overlapping profile of lineage-related transcription factors of cell types of two other model organisms, zebrafish and frog, with reference to the mouse, putative homologous cell types that arise during embryogenesis may be inferred across the three evolutionally diverse species.

Points for clarification:

Data integration and analysis

• One of the key assumptions is that "sampling" were performed "frequently and deeply enough that newly detected cell states will not arise from antecedent cell states that were undetected at the preceding timepoint". It was noted that the datasets used are from 88 developmental timepoints separated by 6 hours to 1 day. A concern is that the transcriptional changes are regulated on the scale of hours during cell differentiation (PMID: 31078527). While the 6-hour gap may be sufficient to capture the successive changes in cell states, the gaps that are larger than this may be a confounding factor. As the frequency of the sampling is a key assumption to capture antecedent cell states for the emergent cell states, the larger time gaps in the data may not hold for such an assumption and could lead to imprecise inference of trajectories.

• A key step in this analysis is to integrate a large number of heterogenous datasets from different developmental time points, generated in different experimental settings and by different sequencing protocols. Sup. Figure 1c-e were to show evidence of effective integration and batch-effort correction. However, these are selected time points and rely largely on visual interpretation. As such, the evidence is rather weak in supporting the effectiveness of the data integration across the whole dataset. For example, while the integration appears to do well in panel d, the results from visual presentation in panel e is less interpretable. It is therefore critical to numerically quantify the quality of integration across each of every time points to assure that all downstream analyses are robust, and the interpretation drawn from these analyses is valid.

• The determination of cell states and types relied primarily on Louvain clustering and the application of marker genes to manually annotation cell types. It is essential to benchmark the robustness of the cell state/type determination by Louvain clustering. In our experience, many clustering algorithms offer the estimation of the number of clusters and the disagreement among them is very high. Furthermore, many of these clustering algorithms are also highly sensitive to small perturbations on the data, leading to different number of estimated clusters. The authors should verify the validity and robustness of the 413 cell states and 84 cell types identified in this study.

• It is not clear how the inference of the approximate spatial location of cell states link to the integrative analysis of the scRNA-seq data. The procedure appeared to be simply running CIBERSORTx with default parameters for cell type deconvolution. It is questionable if new insights can be drawn from this analysis other than corroborating prior knowledge.

• Various heuristic approaches were taken for identifying transcription factors and cis-regulatory motifs involved for cell type specification. The same pipeline was used to characterize zebrafish and

frog embryogenesis and compare with mouse. While it was asserted that these identification and comparisons were systematic, these analyses were highly heuristic and ad-hoc. Although the recapitulation of prior knowledge might support the outcome of analysis cell type homology, further validation would be required to support the claim that the results of this analysis are at "systems" resolution. The ambiguous results of cell type homology, beyond the examples shown (Fig. 6), may stem from the depth of the transcriptome data and quality of the data related to the transcription factor profiles. This limitation may be discussed.

Knowledge gain

• While the TOME provides a global visualization of the molecular trajectory of cells in the embryo up to advanced stages of organogenesis (E12.5-E13.5), which may have bearing of the lineage trajectories during embryogenesis. Much of the outcome is corroborating prior knowledge of lineage relationship gleaned from embryological studies. A few examples of unexpected learning of the molecular drivers at various branching points of the cellular phylogeny and "new" cell types, which were presumed to "pseudo-ancestor" have been highlighted. The concept of "pseudo-ancestor" was not articulated and it is unclear if this is aligned with the notion of "lineage antecedent" or "quasi-progenitors". While it may be a tall order, the merit of this study may be enhanced by experimental validation of the molecular attributes identified that potentially underpinning the transition of "pseudo-ancestors" to emergent cell types embedded in TOME of 413 cell states with 89 cell types (Fig. 4d) or revealed by multilineage priming modelling.

• It is unclear how the data of TOME allow distinguishing between incomplete separation and convergence of cell type in ongoing differentiation. If the anticipated convergence between two cell states (e.g., visceral endoderm and gut (definitive) endoderm) was not supported by the weight analysis, what the relevant biological inference may be drawn from this analysis? Would the analysis of spatial rendered data of cellular heterogeneity or sub-set of data may identify a more credible relationship? How far one may go with this supervised approach like RNA velocity?

• The determination of cell states (Fig. 1) and the candidate TFs in the cellular trajectory (Fig. 4d) appeared to rely primarily on differential gene expression data. An alternative approach may be to anchor the analysis to the stably expressed and repressed genes across multiple steps in the trajectory. This information may be valuable for the elucidation of the transcription factors driving the cell trajectory as the activity of both the up- and down-regulated transcription factors (e.g., Supp Fig 5c and 7) may be instrumental for cell fate choice and identity. It is unclear if the expression of the TF's has any relationship with the enrichment and "diffused enrichment" of motif occupancy. Correlation of the stable changes in gene expression with variations in chromatin accessibility and configuration (e.g., PMID: 33278344), may yield mechanistically relevant information on the molecular activity that accompanies lineage diversification. It may also be informative for elucidate the regulon activity along the molecular trajectory of the cell types.

Reviewer #4:
None

Author Rebuttal to Initial comments

Response to Reviewers

We thank the three reviewers for their constructive feedback on the submitted version of the manuscript. A point-by-point response, which includes summaries of changes made in response to these comments, is provided below. The original reviewer comments are in blue text, while our responses are in black text.

Reviewer 1:

Qiu and colleagues integrated independent single-cell datasets covering most mouse embryonic development to infer the relationships between cell states throughout development. The integration results are overall very good, and the inferred cellular and, to a lesser extent, molecular trajectories recapitulate well what is known from mouse development, showcasing the power of the approach. I enjoyed the authors going further and applying their methodology to analogous datasets for zebrafish and Xenopus and then attempting to identify homologous cell types across vertebrates. This is a very challenging problem given the large evolutionary distances involved, and while the results obtained are far from a final answer, they take us one step further in the right direction. I anticipate this work becoming an important resource for the single-cell, development and evolution communities.

Methodologically, this work is strong; state-of-the-art methods/approaches applied throughout. The figures are excellent, complex procedures and results are made intuitive (e.g., Fig. 4d and Supp. Fig. 2 are terrific). For the most part, the authors did an excellent job laying out the goals, assumptions and limitations of their data and analyses. For example, the discussions of the similarities and differences between the molecular trajectories identified for the mouse and the known cellular phylogenies are very good. This was an enjoyable paper to read, and I heartily congratulate the authors on their work.

We thank the reviewer for these positive comments on the work and its presentation.

1. The integration of the independent single-cell datasets is very good, except for the transition between stages e8.5 and e9.5 when single-cell and single-nuclei datasets are combined. These integration problems are seen in several figures (e.g. Supp. Fig. 1e, Supp. Fig. 5) and likely have downstream consequences. This should be explicitly acknowledged at the start of the manuscript when the integration is first described. If this integration issue creates problems for the inference of cellular trajectories, this should also be explicitly stated, especially if it leads to incongruences with the known cellular phylogenies. Is the e8.5-e9.5 switch the main driver for choosing the 0.2 cutoff for the edge weighs (Fig. 1b)?

We agree that the E8.5 (cells, 10X) → E9.5 (nuclei, sci-RNA-seq3) transition was a major limitation in the submitted manuscript. Numerous cell types appeared or disappeared between these timepoints, and it was unclear which of these changes were due to technical differences vs. *bona fide* developmental progression. It was similarly unclear whether changes in gene expression levels were technical or biological in nature.

Although this was not explicitly requested, we decided that the best way to address this was to generate new data from E8.5 nuclei using sci-RNA-seq3, *i.e.* as a Rosetta stone of sorts between the published Pijuan-Sala *et al.* (2019) and Cao *et al.* (2019) datasets. This new dataset (referred to as

“E8.5b”) ended up being of much higher quality and more interesting than we expected when we set out to generate it, and as such its analysis represents a major portion of the revised manuscript.

In brief, we selected 12 embryos from 2 separate litters harvested at E8.5, including a single primitive streak stage embryo (prior to somitogenesis) and 11 embryos staged in 1-somite increments from 2 to 12 somites. As we have improved sci-RNA-seq3 since 2019, we applied the updated protocol (Martin et al. 2021), which markedly improved data quality, with 9-fold higher UMIs and 6-fold higher gene detection per nucleus, relative to (Cao et al. 2019). This new E8.5 data (nuclei, improved sci-RNA-seq3) enabled the identification of the same 30 cell types as we identified with E8.5 data (cells, 10X) from (Pijuan-Sala et al. 2019) (see new **Fig. 1b-c**, reproduced below).

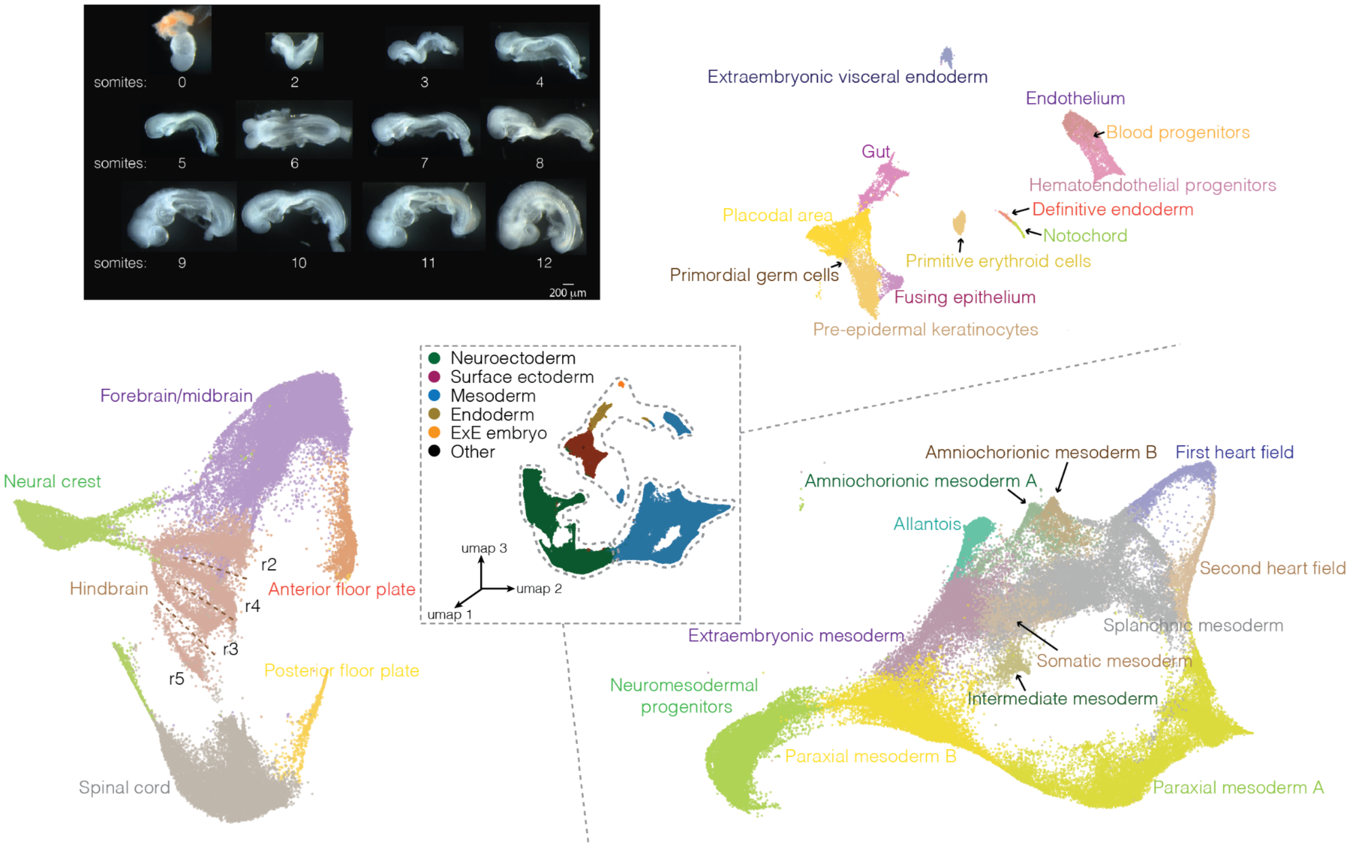

The improvements to dataset integration facilitated by the E8.5b dataset are summarized in new **Supplementary Fig. 2**. In brief, anchor-based batch correction and integration of profiles of E8.5 cells from (Pijuan-Sala et al. 2019) (termed “E8.5a”) and newly generated profiles of E8.5 nuclei (termed “E8.5b”) worked very well with the exception of primitive erythroid cells. As expected because they were generated on nuclei with the same technology, integration of E8.5b and E9.5 profiles also worked well. The improvements in data quality afforded by the updated sci-RNA-seq3 method, as well as by deeper sequencing of the original Cao *et al.* (2019) libraries for E9.5-E13.5, are summarized in new **Supplementary Fig. 3**. The greater resolution of substructure afforded by the new data as well as the availability of somite counts for each profiled E8.5 embryo is presented in new **Fig. 1d-g** and **Supplementary Figs. 4-8** along with corresponding portions of the main text. These new analyses are also summarized below in a response to a query from Reviewer 2.

Regarding the question of how we chose the 0.2 cutoff for the edge weights, we performed the following permutation analysis to choose an appropriate cutoff. Briefly, the same strategy of creating the candidate edges between adjacent timepoints was performed after randomly shuffling the cell-state annotations for cells within each timepoint. This process was repeated 1,000 times to create a null distribution of edge weights. After permutation, fewer than 1% of potential edges are assigned weights greater than 0.2 (new **Supplementary Fig. 9d**, reproduced below).

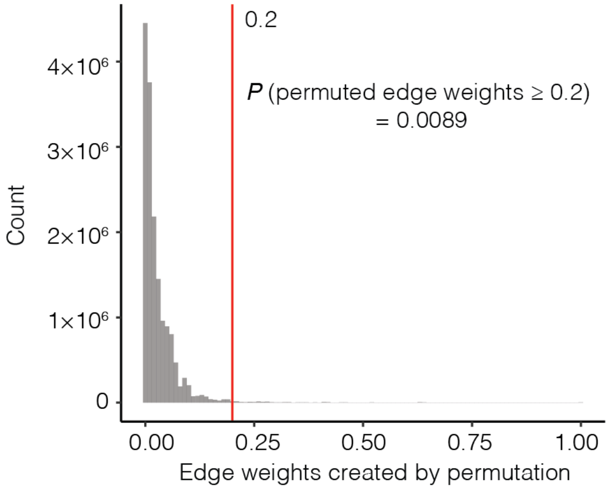

2. In lines 264-267, the authors say there is anecdotal evidence for key transcription factors showing sharp up-regulation upon a cell type's first appearance, whereas other genes have more "gradual patterns of change", offering as an example the genes on Sup. Fig. 5c columns 2&3. However, these genes are not "other genes", they are the genes most positively and negatively correlated with pseudotime, which selected for the "gradual pattern of change". I do not see support for a difference between transcription factors and other genes from the data presented, and the batch effects between stages e8.5 and e9.5 do not help.

The reviewer is correct that we have not done a systematic analysis of TFs vs. non-TFs, and we agree that we should not overgeneralize from the selected examples. We have shortened the corresponding text to simply read: "Nonetheless, at least anecdotally, TFs with established roles in a given cell type were often upregulated in association with its first appearance (**Supplementary Fig. 18**)", which provides a concise transition to the next section, which is focused on the systematic extraction of key TFs across TOME.

3. The authors use pseudotime for their molecular reconstruction analyses arguing that "individual embryos do not correspond precisely to their intended timepoints". Male and female mouse embryos have been reported to develop at different rates, is there a sex component to these staging differences (embryo sex should be readily identified using Xist and Y-linked genes)?

This is an interesting question. For those embryo samples which are not pooled, including 12 samples from the new E8.5 data ("E8.5b") and 61 samples from the deeper sequencing of Cao *et al.* (2019) libraries, we separated them into females (more reads mapping to Xist than chrY genes) and males (more reads mapping to chrY genes than Xist). The estimated pseudotime of male and female embryos from staged timepoints between E8.5b to E13.5 are now shown in **Supplementary Fig. 17c**,

reproduced below. However, a difference in developmental rates is not apparent, except possibly at E10.5, although much larger numbers of embryos would be needed to assess significance. We chose to include the analysis but not comment further on it in the manuscript as we do not think that we have the numbers to draw a clear conclusion one way or the other.

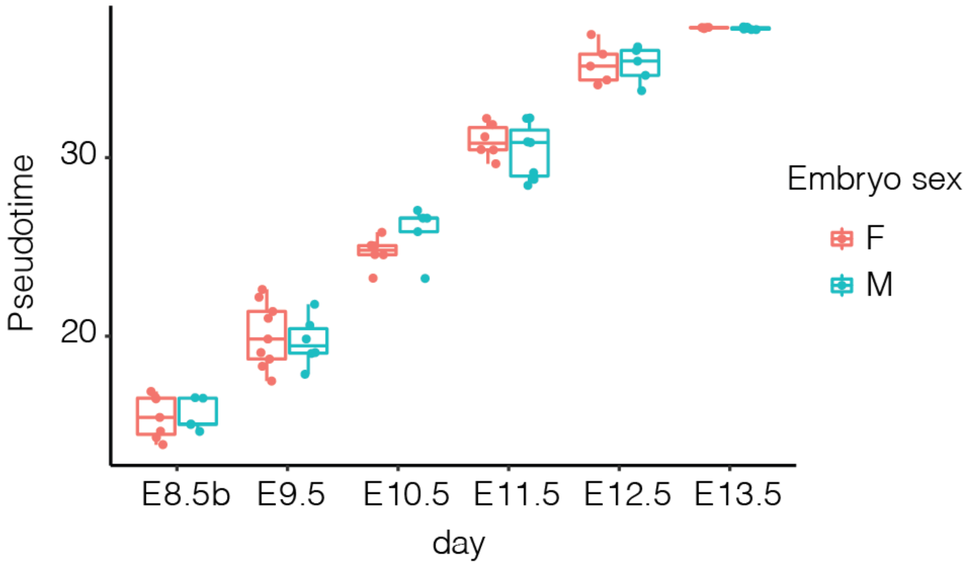

4. Is there a way to systematically evaluate how well the key transcription factors identified match previous literature? Without this comparison, it is hard to know how well the approach used for identifying key transcription factors candidates worked. For example, could the authors add to Fig. 4d information on whether or not the transcription factors identified were already known to play a role in the specification of that particular cell lineage?

This is challenging to accomplish in a systematic way, *i.e.* as PubMed searches may depend on the precise term used for each cell type and each TF, etc., and its inevitably a bit subjective what qualifies as “known to play a role”. However, we attempted to conduct such a search across 533 key TF-cell type relationships and have added a new table (**Supplementary Table 8**) in which, in the cases for which we could find it, 1 or 2 relevant references are provided for the “top” 5 candidate key TFs for each cell type. Of note, we could find relevant references for 494 of 533 key TF-cell type relationships (93%) for which we searched, although many of these correspond to work done in other model organisms or *in vitro* systems.

5. It would also be good to have a way to evaluate how well the approach used to identify cis-regulatory motifs involved in cell-type specification worked. The reported agreement between the key transcription factors identified in the previous section and the enriched binding sites seems poor to me. However, I readily admit that setting expectations for the outcome of this type of analysis is subjective. This is not a sticking point for me; I think this type of question requires ATAC-seq data, but I could see others benefiting from this analysis.

We agree that the agreement between key TFs and the enrichment of their cognate motifs in binding sites of the core promoters of key genes is modest at best, but there are many reasons why this might be the case, including all distal *cis* regulation, which we ignore because we lack corresponding

sc-ATAC-seq data, limitations of the motif database, post-transcriptional mechanisms, etc. We note that we are working to generate an equivalent time series of chromatin accessibility across mouse development. However, we feel that this is beyond the scope of the current manuscript, and the analyses that such a dataset would entail could not be well presented within what is already quite a long paper. We intended for this to be an exploratory analysis that sets the stage for what might be possible when the corresponding sc-ATAC-seq datasets are available, and we hope that the reviewer agrees that it accomplishes at least that.

6. I quite enjoyed the author's attempt to identify homologous cell types across mouse, zebrafish and Xenopus, and the three approaches used are very sensible. I was left very curious about how their third approach would work if, instead of using the key transcription factor candidates, the authors used the 345 +/- 246 key genes associated with the emergence of the cell types.

This is a good suggestion and we attempted this analysis. Unfortunately, the correlated cell types identified by overlapping key genes were noisier than the other approaches. For example, anterior floor plate (mm) was correlated to diencephalon (aplnr2+) (zf) as expected, but it was also correlated to seven other cell types from zebrafish, including erythroid, midbrain ventral, myotome, diencephalon, roof plate, mesoderm lateral plate (tbx1+), dorsal margin involuted. Such noise (which we address by manual review) was also present for the other approaches, but simply less of it with those other approaches. We therefore stuck with the previous approaches as our main strategies. However, as this same thought may occur to readers, we now include the results of the suggested approach in **Supplementary Table 27** and summarize this rationale for not including it in the corresponding section of the Methods.

Minor comments:

1. I don't see a good rationale for using lists of transcription factors for the three species that are 1:1 orthologs with the human set. There are transcription factor databases that include all three species (e.g. AnimalTFDB 3.0). Using 1:1 orthologs makes sense for evolutionary comparisons, but for studying development (within species), it makes sense to use all available genes.

This is a great suggestion. In the revised manuscript, we have switched to using *AnimalTFDB*/v3 (Hu et al. 2019) for the initial systematic nomination of key mouse TFs for cell type specification, as well as for the corresponding analyses of zebrafish and frog. We then use all the possible orthologous gene pairs (see response to next query) when performing the cell-type comparisons between species.

2. In the Methods, the authors should be explicit that in their embeddings and evolutionary analyses, they use sets of genes that are 1:1 orthologs between all species (or pairs of species), not just homologs. Don't forget that paralogs are also homologs. The authors are not using all homologs; they are using orthologs in a 1:1 relationship across species.

Apologies for the confusion. Another reviewer made the same comment, and we were clearly insufficiently detailed in describing our approach. To clarify, in those analyses, we retained all of the possible orthologous gene pairs, including “1-to-1”, “1-to-many” and “many-to-many” categories. We have sought to be clearer on this point in the revised manuscript.

In the analysis of co-embedding of cell states from three species, we first created a list of orthologous genes across the three species by liftover of all gene identities from the three species to the corresponding human gene identities, either based on *BioMart* (Ensembl Genes 102) (Yates et al. 2020) or the original study in the case of frog (Briggs et al. 2018). A list of 22,815 genes was compiled, wherein each of the genes was orthologous in at least two species. We retained all of the possible orthologous gene pairs learned from the BioMart, including “1-to-1”, “1-to-many”, and “many-to-many” categories.

Similarly, in the analysis of identifying correlated cell types between each pair of species, we first created a list of orthologous genes between each pair of species (n = 17,333 for mm vs. zf, n = 14,249 for mm vs. xp, and n = 13,326 for zf vs. xp), either based on *BioMart* (Ensembl Genes 102) (Yates et al. 2020) or the original study in the case of frog (Briggs et al. 2018). Again, we retained all of the possible orthologous gene pairs learned from the *BioMart*, including “1-to-1”, “1-to-many”, and “many-to-many” categories.

3. To this reviewer seeing mammalian in the title and then realizing the work is only for mouse is disappointing. There are already papers comparing development across mammals, and these should be the ones using the word 'mammalian' in the title. Obviously, this is not a sticking point; these are just my two cents.

Fair point. We have changed “mammalian” to “mouse” in the title.

4. In line 69, "delta in time" should be written in plain language.

Agree. We have changed this to “interval between successive timepoints”

5. Please add the colour legend to Figs. 5b,c.

Sorry we missed this. This has been added (now **Fig. 6b-c**).

Reviewer 2:

Qiu et al. have provided a comprehensive re-analysis and integration of existing public scRNA-seq datasets comprising key cell states of the developing mouse, frog, and zebrafish embryos. The study relies heavily on a previously established strategy for reconstructing cell state hierarchies from scRNA-seq data, which was also previously applied to compare vertebrate embryo species (Briggs et al. 2018). The primary achievement of the study is the consolidation of these data into a web resource called “TOME” (trajectories of mammalian embryogenesis), which aims to systematically integrate a “continuous, navigable roadmap of the molecular states of the cell types during mouse development.” In addition to scRNA-seq datasets, the authors have incorporated spatial transcriptomics datasets (GEO-seq), they have identified series of differentially expressed transcription factor genes (and inferred cis-regulatory relationships), and they have performed a cross-species comparison with frog and fish datasets to identify putative conserved “cell type homologs”. The paper is easy to understand, well written, and the methods used are well explained. The authors also clearly articulate the advantages and pitfalls of their approach and analysis. While the approach used here is not particularly

novel, the study performs a much-needed consolidation of existing datasets and delivers an attractive resource.

We thank the reviewer for these positive comments on the work and its presentation.

Overall Comments:

1. Neither the data (see Table S1) nor the primary data analysis strategies (see Briggs et al. 2018) are novel to the present study. The TOME resource (http://tome.gs.washington.edu/) is remarkably similar both in concept and presentation style to previously constructed roadmaps of vertebrate development (Briggs et al. 2018; https://kleintools.hms.harvard.edu/tools/currentDatasetsList_xenopus_v2.html). The notable contribution here is that the resulting resource is for mouse.

These are fair points, and in the original and revised manuscript we have correspondingly sought to be explicit in crediting Briggs *et al.* (2018), *i.e.* "Our primary strategy is inspired by Briggs and colleagues (Briggs et al. 2018)". We very much appreciated the general approach and presentation style in that paper and saw no reason to reinvent the wheel. That being said, we should emphasize that the amount of work and care required to adapt and apply the general strategy to a more extensive window of (mouse) development while also dealing with the technical issues associated with integrating datasets generated by different groups, methods, etc., were substantial, and this was definitely not as simple as applying the Briggs *et al.* strategy out of the box to a few new datasets. This took us several years to get to the point where we were happy with it, *e.g.* the quality of the integrations, annotations, etc.

2. In the TOME graph, developmental relationships between ancestor and descendant nodes and developmental regulatory genes largely confirm expectations but have apparently not generated any new hypotheses (at least, none mentioned by the authors) that were followed up or tested experimentally. Because no major biological discoveries are being reported, the authors could direct their efforts towards strengthening the usefulness of the TOME resource itself.

We concede that we are not reporting major new biological discoveries in this paper. However, that does not mean that new hypotheses are not being generated. For example, the role of nominated key TFs (and key genes) correspond to sets of hypotheses that are inspiring follow-on experiments. In a response to a related comment from Reviewer 1, we conducted a search across 533 key TF-cell type relationships (inclusive of the top 5 key TFs nominated for each cell type) and have added a new table (**Supplementary Table 8**) in which, in the cases for which we could find it, 1 or 2 relevant references are provided for the "top" 5 key TFs that appear for each cell type (as a subset of **Supplementary Table 7**). Of note, we could find relevant references for 494 of 533 key TF-cell type relationships (93%) for which we searched. As the quality of published evidence around each of these relationships varies (and includes relevant references from other model organisms or *in vitro* systems), we are moving forward with systematically perturbing sets of key TFs explicitly nominated by TOME in ESCs as well as mammalian gastruloid models. However, these are complex experiments being conducted by a different set of individuals and their addition would bloat an already long manuscript.

We would further argue that simply verifying and integrating our contemporary understanding of cell type relationships across early mammalian development, built up over several decades of experiments, via a data-driven, largely automated framework, represents an important step forward. As such, we feel the current work stands on its own as a resource for mammalian developmental biology.

Finally, we are reading a bit between the lines here, but we infer that this concern is partly that we are adapting a published strategy (*i.e.* Briggs et al. 2018) and applying it to published data, and that as such the reviewer feels that we are not advancing the ball sufficiently in terms of novelty. On that general point, in response to a comment from Reviewer 1 regarding the poor integration across E8.5 - E9.5, we have performed new, intensive profiling of ~150,000 nuclei from a series of individual, somite-resolved E8.5 embryos with an improved combinatorial indexing protocol. These data and the ensuing analyses add novelty to the manuscript, critically bridging what had been a problematic transition, while also enabling greater resolution of substructure and temporal dynamics around E8.5 than was possible with the Pijuan-Sala *et al.* data (generated from pooled embryos) alone. In response to a different comment from this reviewer, we have also performed deeper sequencing of E9.5 - E13.5 libraries reported in Cao *et al.* (2019), and reanalyzed that data as part of incorporating it to TOME.

In more detail, new data additions include:

1) <u>New, somite-resolved E8.5 data using optimized sci-RNA-seq3</u>: A limitation of TOME in the submitted manuscript is that the integration between E8.5 (cells, 10X Genomics) and E9.5 (nuclei, sci-RNA-seq3) was problematic. Numerous cell types appeared or disappeared between these timepoints, and it was unclear which of these changes were due to technical differences vs. *bona fide* developmental progression. To address this, we sought to generate new data from E8.5 nuclei using sci-RNA-seq3, *i.e.* as a Rosetta stone of sorts between the published Pijuan-Sala *et al.* (2019) and Cao *et al.* (2019) datasets.

In brief, we selected 12 embryos from 2 separate litters harvested at E8.5, including a single primitive streak stage embryo (prior to somitogenesis) and 11 embryos staged in 1-somite increments from 2 to 12 somites. As we have improved sci-RNA-seq3 since 2019, we applied the updated protocol, which markedly improved data quality, with 9-fold higher UMIs and 6-fold higher gene detection per nucleus, relative to (Cao et al. 2019). The improvements to dataset integration facilitated by the E8.5b dataset are summarized in new **Supplementary Fig. 2**. Anchor-based batch correction and integration of profiles of E8.5 cells from (Pijuan-Sala et al. 2019) (termed “E8.5a”) and newly generated profiles of E8.5 nuclei (termed “E8.5b”) worked very well with the exception of primitive erythroid cells. As expected because they were generated on nuclei with the same technology, integration of E8.5b and E9.5 profiles also worked well.

This new E8.5 data (nuclei, improved sci-RNA-seq3) enabled the identification of the same 30 cell types as we identified with E8.5 data (cells, 10X) from (Pijuan-Sala et al. 2019) (see new **Fig. 1b-c**, reproduced on the next page). Importantly, analysis of this new dataset also revealed multiple new cell subpopulations and improved trajectories as further described below.

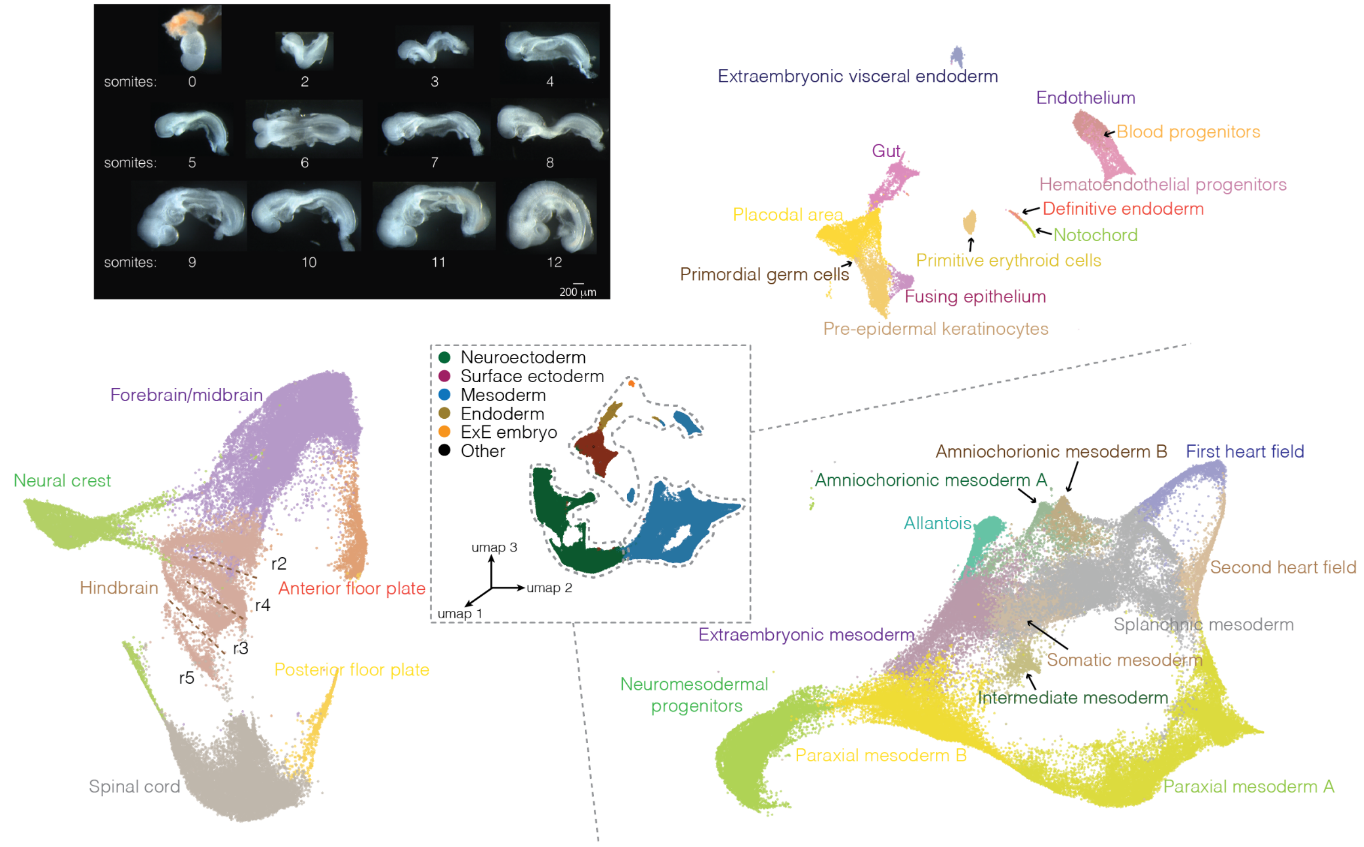

2) <u>Deeper sequencing on the original libraries for E9.5-E13.5</u>: To obtain higher quality data across E9.5 to E13.5, we performed a deeper sequencing (specifically, three additional Novaseq runs) of our previously reported libraries (Cao et al. 2019). We merged the new and previously regenerated reads and reran sci-RNA-seq3 data-processing. Compared to the original study, the median UMI count per nucleus improved from 671 to 1,434, while the median genes detected per nucleus improved from 518 to 735. These improvements, together with the improvements at E8.5 afforded by the updated sci-RNA-seq3 method, are summarized in new **Supplementary Fig. 3**, reproduced below.

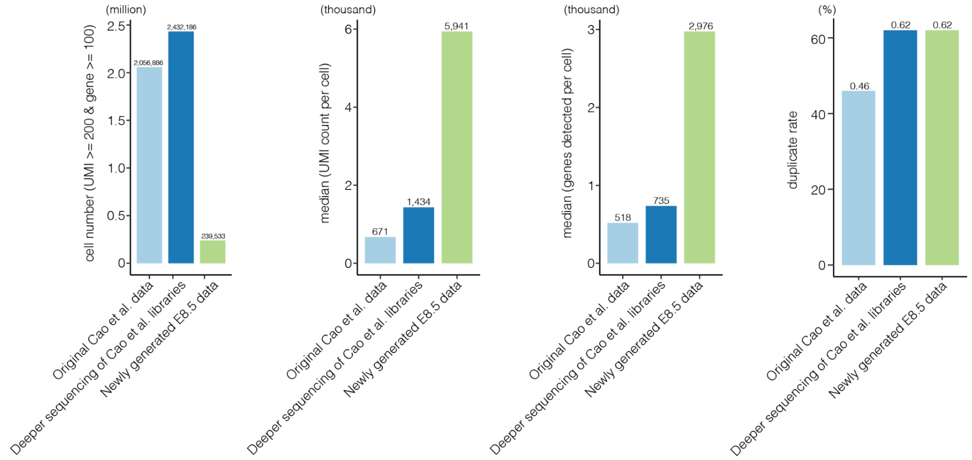

New observations enabled by these new data include:

1) Substructure observed from intensive profiling at E8.5: The depth of the new data, together with the fact that we separately processed individual somite-resolved embryos, facilitated the resolution of substructure beyond what was described at E8.5 by Pijuan-Sala *et al.* (2019) or in our original manuscript. Examples include: 1) Floor Plate: We observe two, clearly distinct subpopulations that express floor plate markers *Foxa2* and *Shh* (**Supplementary Fig. 4**) (Placzek and Briscoe 2005), one inferred to be anterior (*Bmp7*+) and the other posterior (Dale et al. 1999). 2) Heart fields: We observe subpopulations arising from the splanchnic mesoderm that correspond to the first (*Tbx5*+, *Hcn4*+) and second (*Isl1*+, *Tbx1*+) heart fields (**Supplementary Fig. 5**) (Rana et al. 2014; Cai et al. 2003; Herrmann et al. 2011; Später et al. 2013). 3) Rhombomeres: We observe four subpopulations of the hindbrain, as well as two additional subpopulations within midbrain and spinal cord, that we can correspond to rhombomeres r1 to r6 (**Supplementary Fig. 6**, a portion of which is reproduced below). 4) Neural crest: We observe three distinct subpopulations of neural crest cells (NCC) that appear to derive from different subsets of neuroectoderm, which may correspond to mesencephalic & pharyngeal arch 1 (PA1) NCC; PA2 NCC; and PA3 NCC (**Supplementary Fig. 7**).

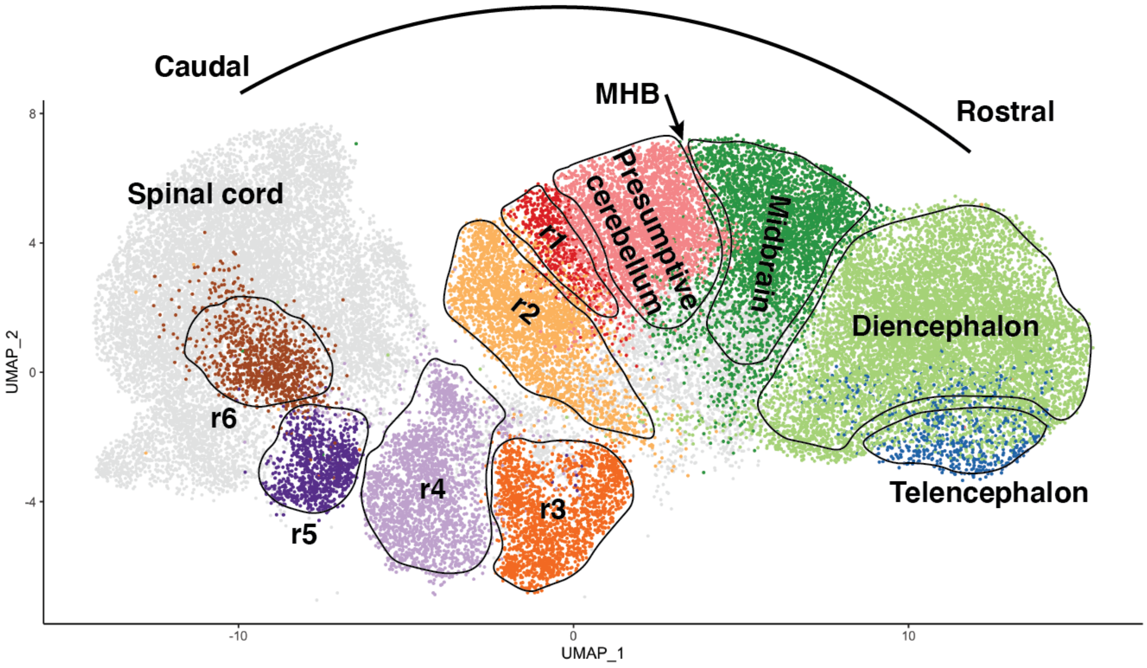

2) Transcriptional dynamics across early somitogenesis: We leveraged the new E8.5 data, derived from the individual, somite-resolved embryos, to systematically explore the extent to which the transcriptional dynamics of individual cell types are coordinated with the timing of somite formation. Interestingly, changes within neuroectodermal cell types (*e.g.* hindbrain, neuromesodermal progenitors (NMPs), floor plate, neural crest, etc.) are more correlated with somite number than the somites themselves are, consistent with rapid, synchronized changes in the developing CNS (**Fig. 1f**). Moreover, we were able to observe, within each individual cell type at E8.5, the composition of cells from embryos with different somite counts. For example, each rhombomere includes cells from embryos spanning somitogenesis, suggesting roughly concurrent, rather than sequential, differentiation of these rhombomeres. However, a subset of cells from rhombomere 4 are from the earliest embryos of the series and express *Hoxa1*

and *Hoxb1*, consistent with the possibility that rhombomere 4 begins to develop first ((Maves, Jackman, and Kimmel 2002; Studer et al. 1998)) (shown in reproduced figure panels below, which highlight the “early” subset of cells from rhombomere 4 with a red circle).

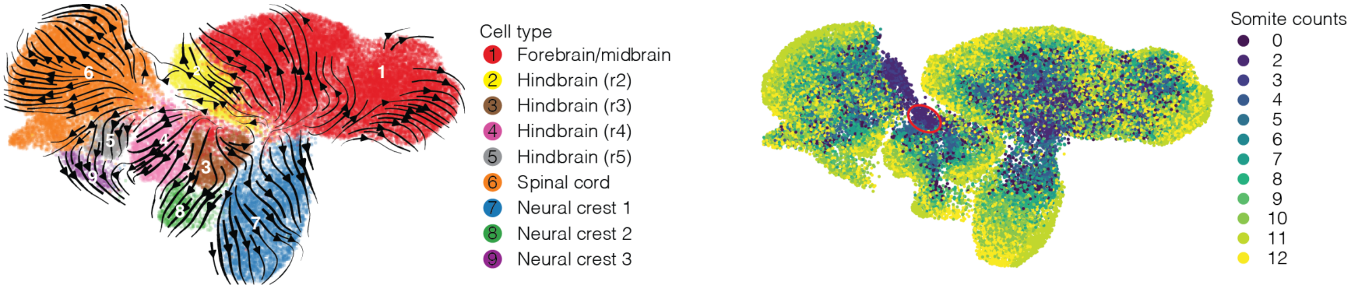

3) <u>Decoding transcriptional heterogeneity of NMPs during somitogenesis</u>: Focusing on NMPs, whose heterogeneous states bridge paraxial mesoderm and spinal cord neuroectoderm, we observe that the top principal components of transcriptional variation are strongly correlated with mesodermal (*T* (Brachyury)+, *Tbx6*+) vs. neuroectodermal (*Sox2*+) state (PC1; 23.7% of variation), cell cycle index (PC2; 15.1% of variation) and somite count (PC3; 8.4% of variation) (Javali et al. 2017; Sambasivan and Steventon 2020). The genes most highly correlated with these PCs are shown in **Fig. 1g**, reproduced below. For example, key regulators of mesoderm (*T*) (Wilson et al. 1995), the somite segmentation clock (*Hes7*) (Hirata et al. 2004) and Wnt signaling (*Wnt3a, Rspo3, Ptk7*) (Berger, Wodarz, and Borchers 2017; de Lau, Snel, and Clevers 2012) are positively correlated with PC1, while regulators or effectors of neural adhesion or neurite outgrown (*Ptprz1, Nrcam, Ptn*) (Shintani et al. 1998; Sakurai 2012; Tang et al. 2019) as well as retinoic acid signaling (*Rarb*) are negatively correlated.

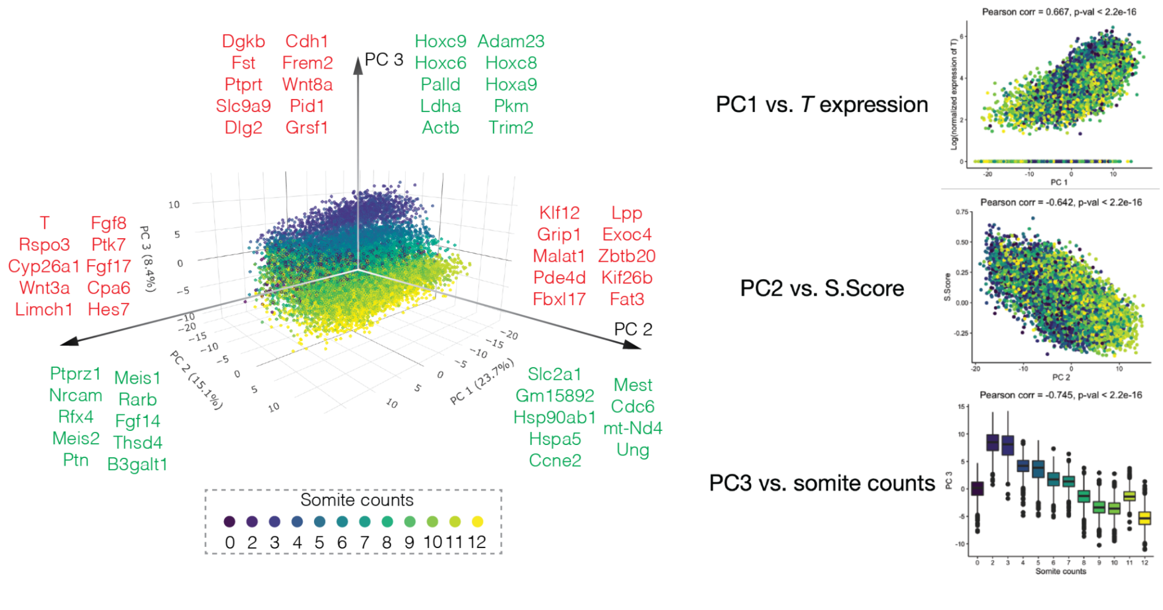

4) <u>Consolidating and improving the reconstructed cellular trajectories</u>: By including the new E8.5 data as well as the deeper sequencing of the previous libraries, we have improved the resolution and coherence of TOME, *e.g.* 94 rather than 84 cell types are now annotated, and the relationships

between cell types at the E8.5 to E9.5 transition are much clearer. Although this is a work in progress and challenging to incorporate into the graph-based representation, we note that continuous aspects of spatial heterogeneity might be retained in co-embeddings across timepoints. For example, for neural tube derived cells from E8.5b and E9.5, the co-embedding is potentially informative in both directions to advance the resolution of annotations.

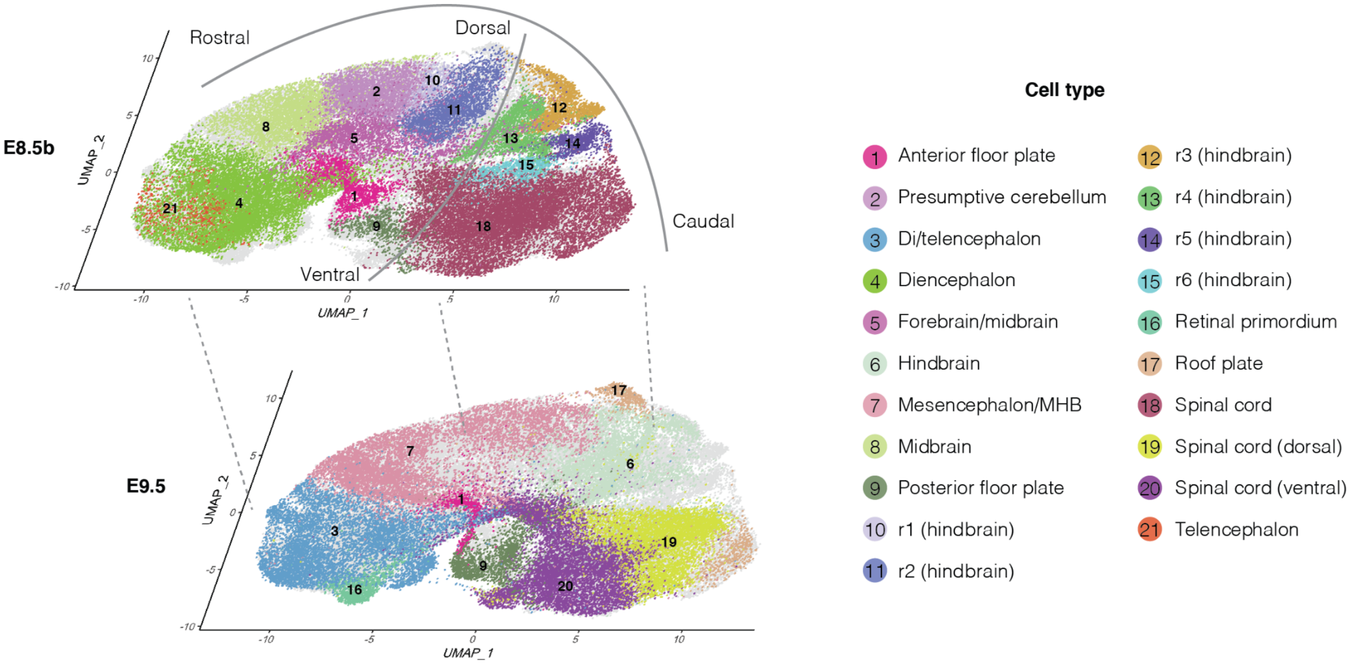

In summary, we believe that both from the perspective of novel data and insights, as well as in terms of the quality of TOME, the revised manuscript is substantially improved.

Given that the overall goal of this study is to generate a “systematic integration” of different ‘omics datasets and technologies, it was somewhat disappointing that TOME did not integrate the spatial-omics (GEO-seq), inferred cis-regulatory, or RNA-velocity information, which at present are a bit disconnected from the rest of the study. Could this information be directly incorporated into the TOME resource? Given that the authors have argued in Fig 2 that RNA velocity has indeed helped to resolve specific ambiguities, perhaps such information could be incorporated in the final calculation of edge weights of the TOME graph? Such a strategy has indeed been successfully demonstrated for refining edge weights in a cell state graph in other contexts (e.g. PAGA, Wolf et al. 2019) and could be worth considering here.

Thank you for this suggestion. To approach this more systematically, we calculated edge weights between cell states at adjacent timepoints with an alternative heuristic that was based on RNA velocity. Specifically, we integrated cells from each pair of adjacent timepoints, and then applied RNA velocity analysis using *scVelo* (Bergen et al. 2020). The resulting transition probabilities between individual cells (stored in a *velocity_graph* matrix), were calculated using cosine correlation between the potential cell-to-cell transitions and the inferred velocity vector (ranging from 0 to 1). To calculate the transition probability from cell state *A* at the earlier timepoint to cell state *B* at the later timepoint, we summed the transition probabilities of all cells within *A* to all cells within *B*, followed by normalizing the total cell

number of *B*. Finally, the edge weight from *A* to *B* was further calculated by normalizing their transition probability to the total transition probabilities which originated from *A*.

Comparing these heuristics, we found that out of 515 *k*-NN nominated edges with weights > 0.2, 392 had RNA velocity-based transition probabilities > 0.2 (76%) (see **Supplementary Fig. 14**, reproduced below). However, there were also 123 edges nominated by the *k*-NN strategy only, and 75 edges nominated by the RNA velocity strategy only (see panel c of **Supplementary Fig. 14**, reproduced below). Although we may assign greater confidence to edges nominated by both methods, edges supported by one method or the other may include both true and false positives. As an example of a likely true positive supported by the RNA velocity method only, the connection between embryonic visceral endoderm (E8.0) and gut (E8.25), fell short of the edge threshold by the *k*-NN strategy (weight 0.14) but was strongly supported by the RNA velocity strategy (weight 0.96).

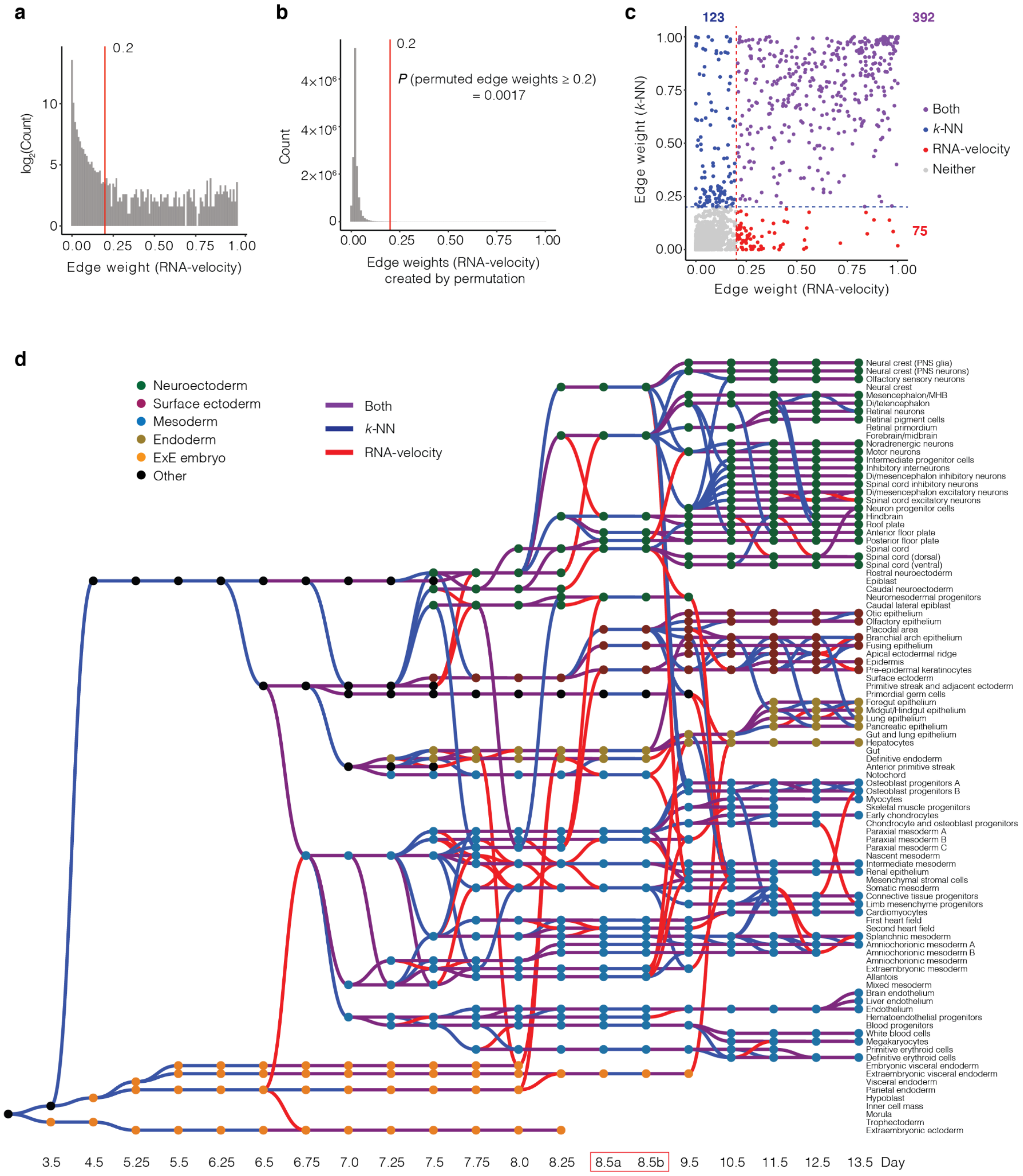

3. The online TOME resource should include the zebrafish and frog analyses. At present, only mouse is available.

Thank you for this suggestion. We have updated the website to include zebrafish and frog as well.

4. The code provided at http://tome.gs.washington.edu/ is insufficient to reconstruct the analyses presented in the paper.

Thanks for pointing this out. We have reorganized the scripts to enable reconstruction of the presented analyses and posted a link to the Github page where they reside on the website.

Specific Comments:

1. In general, the complexity and diversity of cell state structures is expected to increase with developmental time. However, the datasets used in this study (Supplementary Fig. 1b) utilize only minimal sampling depths in the later timepoints. The datasets for the final timepoints (E9.5-E13.5) appear to range from ~500 to ~2000 UMI per cell (the true values are a bit masked by the log2 scale of the y-axis). Can the authors comment on the potential limits of this low sampling depth on their ability to distinguish cell types with highly similar transcriptomes?

Thank you for this comment. As noted above, to obtain higher quality data across E9.5 to E13.5, we performed a deeper sequencing (specifically, three additional Novaseq runs) of our previously reported libraries (Cao et al. 2019). We merged the new and previously regenerated reads and reran sci-RNA-seq3 data-processing. Compared to the original study, the median UMI count per nucleus at these timepoints increased with deeper sequencing from 671 to 1,434. Also as noted above, the new E8.5b data was generated using an improved version of sci-RNA-seq3, resulting in substantially higher UMI counts per nucleus (median 5,941) than we have for E9.5 - E13.5.

Despite these improvements, the UMI counts for sci-RNA-seq3 data still fall short of what is obtained at earlier timepoints on the 10X platform (median 15,136 UMIs for the Pijuan-Sala *et al* (2019) study, across all timepoints; see reproduced figure panel below). This is partly because of our use of nuclei rather than cells (nuclei contain less RNA), and partly due to differences between the technologies. The disadvantage is offset by advantages including a likely fewer cell type-specific biases in isolating nuclei from whole embryos than is the case with cells, the ability to resolve individual embryos rather than needing to rely on pools, and the much greater number of cells/nuclei that can be affordably profiled with sci-RNA-seq3.

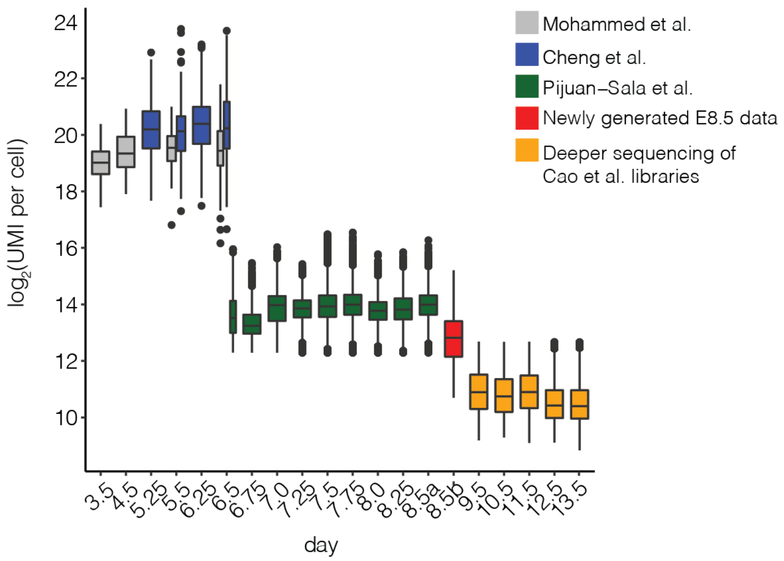

To the specific question of whether this impacts our ability to distinguish cell types, we note that for E8.5, the only timepoint for which we have both 10X and sci-RNA-seq3 data, we are readily able to identify all of the same 30 cell types with sci-RNA-seq3 data that were annotated with 10X data (the latter annotations largely drawing on Pijuan-Sala study). We were moreover able to go even deeper in terms of resolution than what was presented and discussed in the Pijuan-Sala study (*e.g.* first & second heart fields, anterior & posterior floor plate, individual rhombomeres, correlations with somite number, etc.). We recognize that the E8.5b data is of higher quality than the E9.5-E13.5 data, but it is challenging to know whether there is an impact at E9.5-E13.5 because we do not have 10X data at those timepoints. However, we at least do not observe extensive 'collapsing' of cell types as might be expected if our resolution were substantially decaying.

2. For the TOME analysis, the authors consider 0.2 as a cutoff to filter edges. What is the rationale for using this cutoff? Could the magnitudes of these edge weights be checked for significance, e.g. by repeating the analysis on randomized data?

To choose the 0.2 cutoff for the edge weights, we performed the following permutation analysis. Briefly, the same strategy of creating the candidate edges between adjacent timepoints was performed after randomly shuffling the cell-state annotations for cells within each timepoint. This process was repeated 1,000 times to create a null distribution of edge weights. After permutation, fewer than 1% of potential edges are assigned weights greater than 0.2 (new **Supplementary Fig. 9d**, reproduced below).

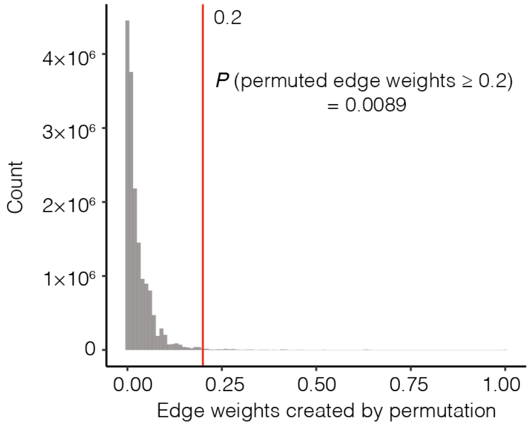

3. How are “dummy nodes” constructed?

Apologies for any confusion. In the directed acyclic graph presented in **Fig. 2c**, we introduced 4 “dummy nodes”, corresponding to morula at E3.0 (as a root for trophectoderm and inner cell mass), trophectoderm at E3.5 and E4.5 (which had been removed at these timepoints by immunosurgery (Mohammed et al. 2017)) and parietal endoderm at E6.75 (undetected, likely due to undersampling). By “dummy nodes”, we simply mean that these nodes were not derived from the data but rather inserted into the directed acyclic graph. In the revision, because the integration of sci-RNA-seq3 and 10X data at E8.5 was problematic (solely) for primitive erythroid cells, we also introduced an artificial edge to connect these cell states across E8.5a and E8.5b.

4. Page 7 - Sagner et al., nd?

Sorry that was a typo. The reference should be “Sagner et al. Establishing neuronal diversity in the spinal cord: a time and a place. Development (2019).” (PMID: 31767567). This has been fixed.

5. From Fig. 3D, the authors conclude that they see a convergence of visceral and definitive endoderm. By convergence, here do they mean co-localization? If so, there does not seem to be a complete overlap. Can the authors comment on the potential significance and/or confidence of this result?

Apologies -- we did mean convergence, but the citation to Fig. 3D (now **Fig. 4d**) was not sufficient to see what we were referring to. It is easier to see in **Supplementary Fig. 16**, if one tracks the positioning of these two lineages over time, and we have now added a citation to that figure at the end of this sentence as well. In any case, we agree that the resolution of the GEO-seq data is limited. We have added a clause noting that the overlap is not complete (although it may be too early to expect it to be complete, and GEO-seq data is not available for later timepoints).

6. For integration with GEO-Seq, the authors downsample to 50 cells - is this random sampling and are these patterns reproducible with different subsets of cells?

Thank you for this suggestion. The previous analysis was based on random downsampling of each cell state to 50 cells. The reason that we did the downsampling was to reduce the compute time of running *CIBETSORTx*. In the revised manuscript, we have repeated this analysis but including all the cells from each cell state. In addition, we compared the new results with the previous results based on

downsampling. Generally, the inferred cell state proportions of each GEO-seq territory are robust to downsampling (**Supplementary Fig. 15**; reproduced below)

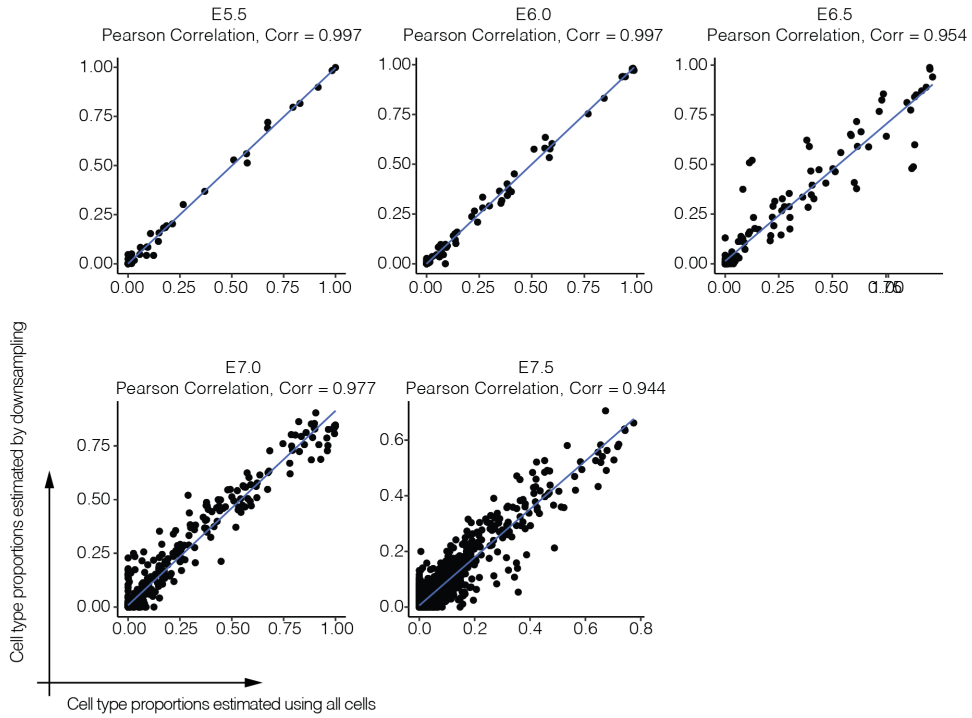

7. Supplementary Fig. 1E – it would be informative to include a version of the UMAP before batch correction.

Thank you for this suggestion. We now present UMAP visualizations of co-embedding on cells from E8.5a (Pijuan-Sala et al.) and nuclei from E9.5 (after deeper sequencing) before and after batch correction (**Supplementary Fig. 1c-d;** reproduced below). The point is to illustrate a context where the success of batch correction is at its nadir. As noted above, we have generated new E8.5 data (termed E8.5b) using optimized sci-RNA-seq3, and we now also show co-embedding results of: 1) E8.5a and E9.5; 2) E8.5a and E8.5b; 3) E8.5b and E9.5 in **Supplementary Fig. 2**, also reproduced below.

Co-embedding of E8.5a (cells, 10X) and E9.5 (nuclei, sci-RNA-seq3), pre- and post- batch correction:

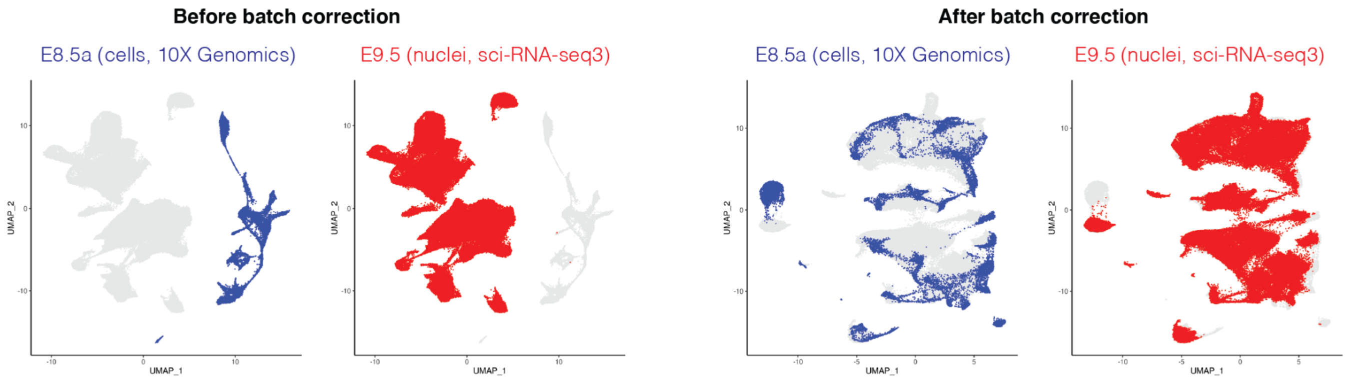

Co-embedding of E8.5a & E9.5, E8.5a & E8.5b, E8.5b & E9.5, all post batch correction:

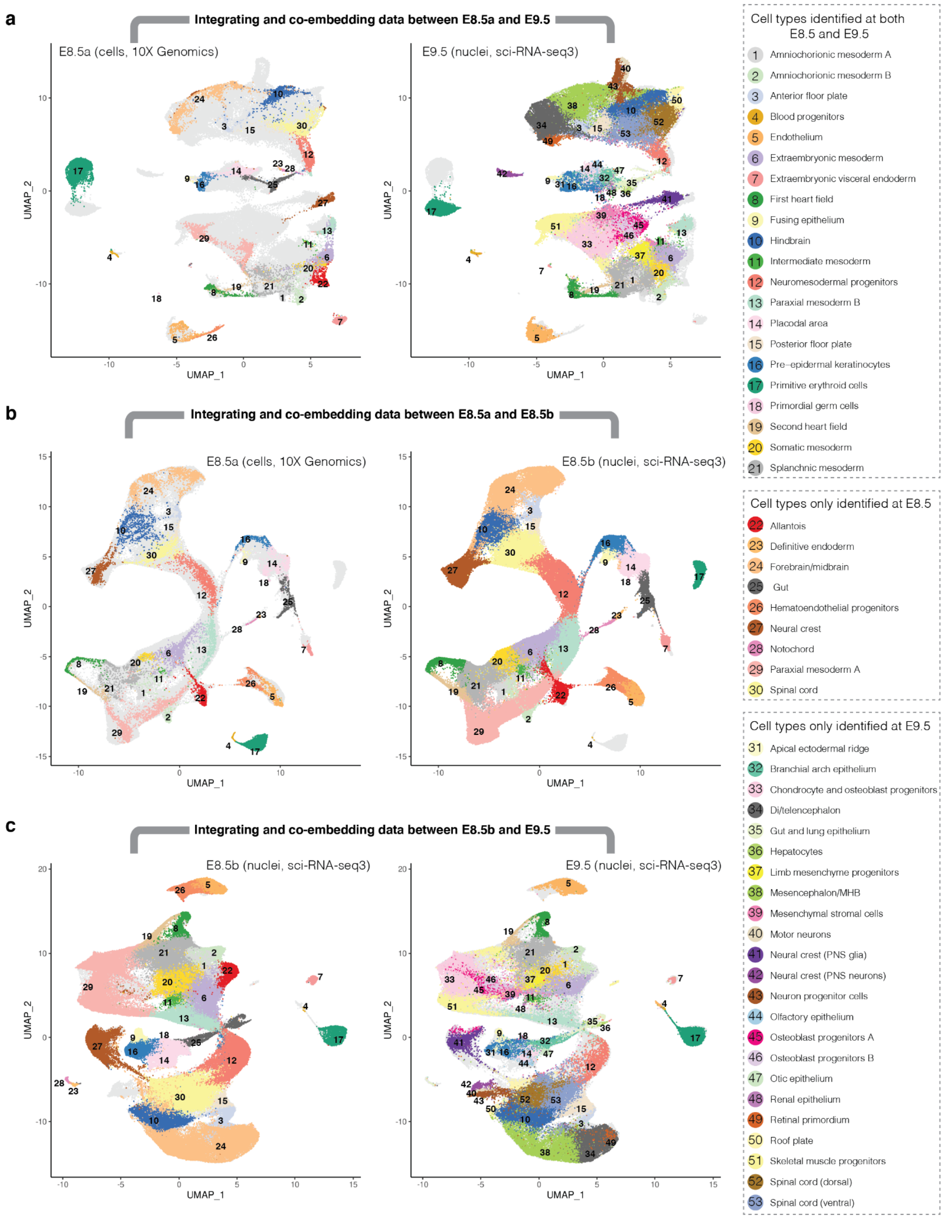

8. Authors identify targets of SNAIL1 and RFX3. The authors might consider performing an experimental validation of the repressive and/or inductive activity of these TFs towards candidate target genes identified in their analysis.

Thanks for this suggestion. We are undertaking the development of new methods to scalably test such potential TF-regulatory element interactions in the context of dynamic, *in vitro* models of early development, *e.g.* gastruloids and similar systems. We would argue that such *in vitro* models that include gastrulation are in fact critical to be able to meaningfully interpret results that would come out of a validation experiment (*e.g.* simply overexpressing these TFs in a cancer cell line and hoping that the corresponding genes are activated would not be terribly satisfying, regardless of the outcome). Developing and implementing assays of reporter activity in a dynamic, multi-cell type model is a major undertaking, and although we are committed to this line of work, we would argue that it is beyond the scope of this already long paper.

9. The authors should describe in detail how transcript counts were compared in the multi-species alignments. Were only 1-to-1 gene ortholog relationships considered? If so, how were these orthologous relationships determined? The authors may consider benchmarking their cell type alignments to those of a recent study (SAMAP, Tarashansky 2020, bioRxiv) that also aligns the same zebrafish and frog scRNA-seq datasets.

Apologies for the confusion. Another reviewer made the same comment, and we were clearly insufficiently detailed in describing our approach. To clarify, in those analyses, we retained all of the possible orthologous gene pairs, including “1-to-1”, “1-to-many” and “many-to-many” categories. We have sought to be clearer on this point in the revised manuscript.

In the analysis of co-embedding of cell states from three species, we first created a list of orthologous genes across the three species by liftover of all gene identities from the three species to the corresponding human gene identities, either based on *BioMart* (Ensembl Genes 102) (Yates et al. 2020) or the original study in the case of frog (Briggs et al. 2018). A list of 22,815 genes was compiled, wherein each of the genes was orthologous in at least two species. We retained all of the possible orthologous gene pairs learned from the *BioMart*, including “1-to-1”, “1-to-many”, and “many-to-many” categories.

Similarly, in the analysis of identifying correlated cell types between each pair of species, we first created a list of orthologous genes between each pair of species (n = 17,333 for mm vs. zf, n = 14,249 for mm vs. xp, and n = 13,326 for zf vs. xp), either based on *BioMart* (Ensembl Genes 102) (Yates et al. 2020) or the original study in the case of frog (Briggs et al. 2018). Again, we retained all of the possible orthologous gene pairs learned from the *BioMart*, including “1-to-1”, “1-to-many”, and “many-to-many” categories.

As suggested by the reviewer, we compared our cell-type alignments between zebrafish vs. frog to a recent study (Tarashansky et al. 2021) that also aligns the same datasets. We could find consistent alignments for 35 of 46 pairs of cell types which they identified (**Supplementary Table 28**). Note that neither we nor they simply used the original data, but rather we re-processed them in different ways. For example, we combined sc-RNA-seq data from two zebrafish studies followed by reannotation of the merged set of cells from each individual timepoint, while the other study sometimes merged multiple

cell types into one (optic cup and retina pigmented epithelium → optic). These differences make a full comparison challenging. Nonetheless, this initial comparison of these entirely independent efforts are mostly in agreement, which is encouraging.

Reviewer 3:

This report described a data analytics paradigm that integrates the single-cell transcriptome dataset of cell population from mouse embryos at pre-implantation (E3.5) to stages of organogenesis (E13.5) and collates the molecular trajectory of cell types during mouse embryogenesis, coined as TOME. Through the mining of TOME, the nominal phylogenetic relationship of cell types and the molecular drivers of cell type diversification, in particular the engagement of combinatorial transcription factors in specific steps of the phylogeny can be retrieved from the dataset. Cross analysis of the transcriptome and overlapping profile of lineage-related transcription factors of cell types of two other model organisms, zebrafish and frog, with reference to the mouse, putative homologous cell types that arise during embryogenesis may be inferred across the three evolutionally diverse species.

Points for clarification:

Data integration and analysis

• One of the key assumptions is that "sampling" were performed "frequently and deeply enough that newly detected cell states will not arise from antecedent cell states that were undetected at the preceding timepoint". It was noted that the datasets used are from 88 developmental timepoints separated by 6 hours to 1 day. A concern is that the transcriptional changes are regulated on the scale of hours during cell differentiation (PMID: 31078527). While the 6-hour gap may be sufficient to capture the successive changes in cell states, the gaps that are larger than this may be a confounding factor. As the frequency of the sampling is a key assumption to capture antecedent cell states for the emergent cell states, the larger time gaps in the data may not hold for such an assumption and could lead to imprecise inference of trajectories.

We fully agree with the reviewer. In ongoing work, we are working to rectify this by sampling post-E8.5 development at higher temporal resolution, *i.e.* every 6 hours or even more frequently. From a logistical perspective (*i.e.* timing of matings vs. collections being out of sync with mouse facility staff work schedules), this is very challenging to execute on. However, we have partnered with Jackson Kabs (JAX) and it is underway. Indeed, the new E8.5b data are from the early stages of such collections at JAX, and showcase the power of careful staging to enable even higher temporal resolution than 6 hour gaps (*e.g.* embryos staged in 1-somite increments from 2 to 12 somites). The full set of embryo collections and data generation may take another year or more to complete, and we believe that it is beyond the scope of the current manuscript. However, we do think that the reviewer's point could be better emphasized. We now say in the discussion: "Of note, by profiling individual embryos staged around E8.5, we observe dramatic changes in gene expression for some cell types (*e.g.* hindbrain, NMPs) occurring within short periods of time (1 somite or ~2 hour increments). Particularly at later timepoints, it remains possible that our daily temporal sampling compromises our assumption that antecedent states will be relatable to descendent states. As mouse development is further profiled, improving temporal resolution should be a high priority."

• A key step in this analysis is to integrate a large number of heterogenous datasets from different developmental time points, generated in different experimental settings and by different sequencing protocols. Sup. Figure 1c-e were to show evidence of effective integration and batch-effort correction. However, these are selected time points and rely largely on visual interpretation. As such, the evidence is rather weak in supporting the effectiveness of the data integration across the whole dataset. For example, while the integration appears to do well in panel d, the results from visual presentation in panel e is less interpretable. It is therefore critical to numerically quantify the quality of integration across each of every time points to assure that all downstream analyses are robust, and the interpretation drawn from these analyses is valid.

Thanks for pointing this out. To address this comment, we sought to develop a summary metric corresponding to the quality of integration between adjacent timepoints. For each co-embedding, we focused on cells at the later timepoint assigned to annotations that were also present at the earlier timepoint. We then calculated the fraction of these cells' ancestral *k*-nearest neighbors (in the global 3D UMAP co-embedding, post-batch correction) that were assigned the identical annotation. The mean proportion for different values of *k* are reported in the below histogram (which also in the revised manuscript as **Supplementary Fig. 9e**). We observe a modest decrease in these proportions (which are robust to the choice of *k*) in later timepoints, possibly due to the greater complexity of cell types that are present in the co-embeddings or alternatively due to lower data quality or lower temporal resolution at timepoints profiled with sci-RNA-seq3 (although the slow decline, even within the Pijuan-Sala timepoints, supports the first hypothesis). Nonetheless, the relatively high proportion of 'concordant' neighbors across time suggests reasonably high and consistent quality of data integration. Further, as we now note in the legend of this figure panel, "the lower value of this metric for E8.5a-E9.5 (red label) than E8.5a-E8.5b or E8.5b-E9.5 provides quantitative support for our claim that the new E8.5b data improved integration across the E8.5 to E9.5 (**Supplementary Fig. 2**).".

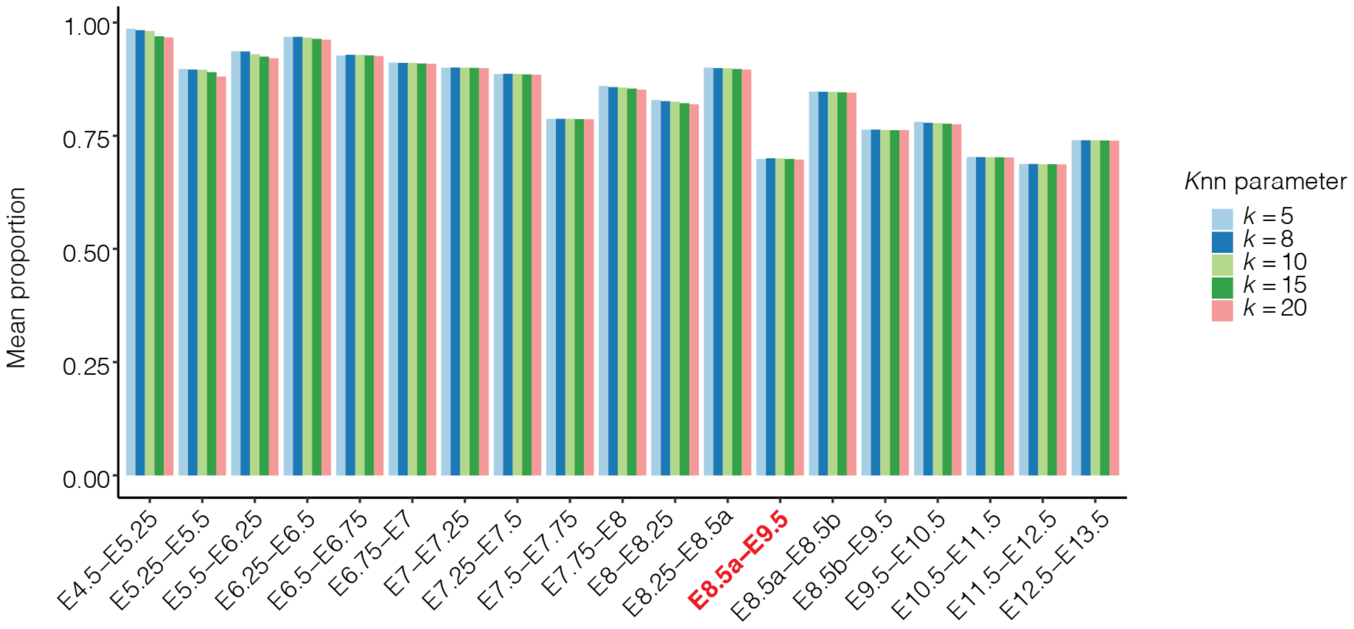

• The determination of cell states and types relied primarily on Louvain clustering and the application of marker genes to manually annotation cell types. It is essential to benchmark the robustness of the cell state/type determination by Louvain clustering. In our experience, many clustering algorithms offer the estimation of the number of clusters and the disagreement among them is very high. Furthermore, many of these clustering algorithms are also highly sensitive to small perturbations on the data, leading to different number of estimated clusters. The authors should verify the validity and robustness of the 413 cell states and 84 cell types identified in this study.

Thank you for this comment. To quantify the robustness of cell type annotations, we applied the *sklearn.svm.LinearSVC* function in scikit-learn/1.0 with 5-fold cross-validation, using the expression values of all genes as predictors. In the below figure (which is also in the revised manuscript as **Supplementary Fig. 10**), each heatmap shows the confusion matrix between true cell-type labels (rows) and predicted cell-type labels (columns) for cells within each individual timepoint, normalized to total counts per column (i.e. each column sums to one). The accuracy (*Acc*) across the whole matrix is shown above each heatmap. Overall, the high accuracy across timepoints indicate our cell type annotations are relatively robust.

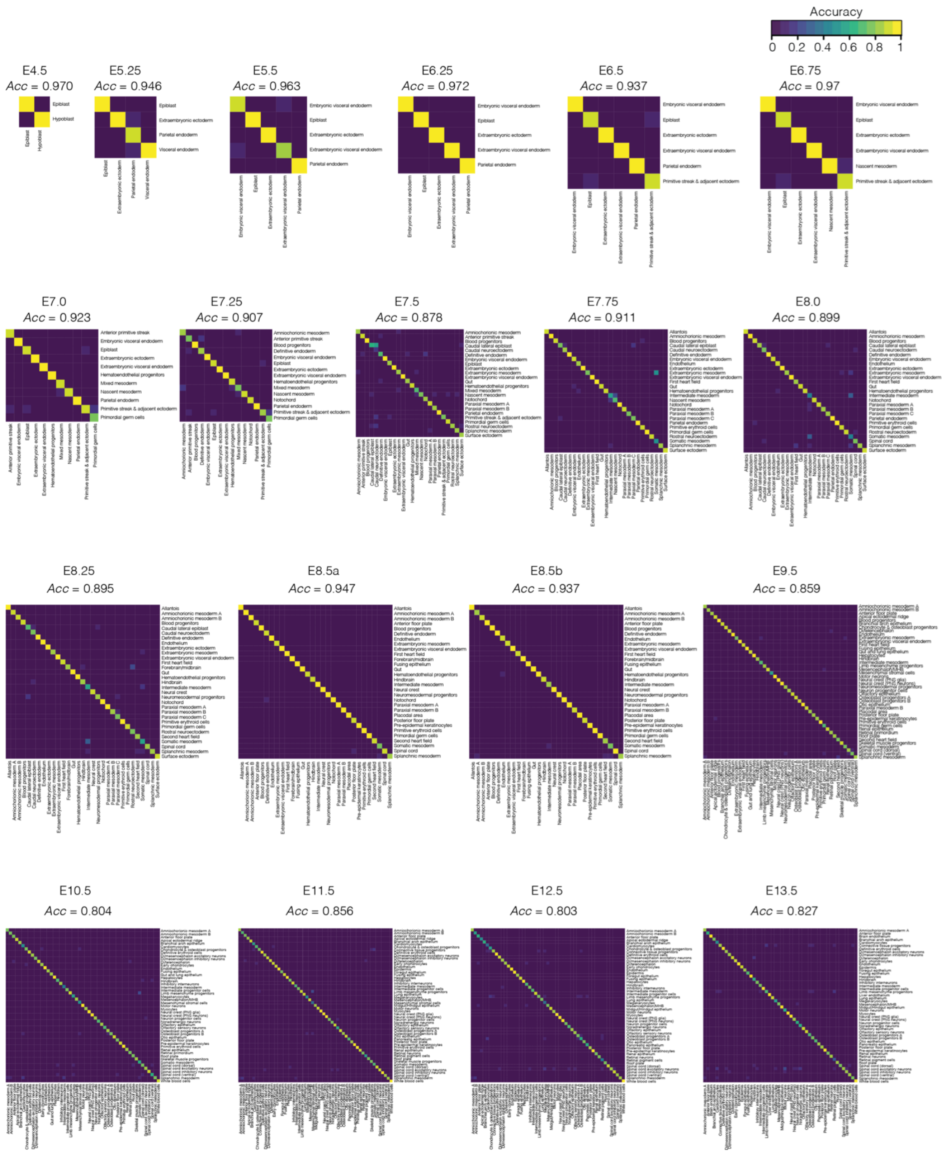

• It is not clear how the inference of the approximate spatial location of cell states link to the integrative analysis of the scRNA-seq data. The procedure appeared to be simply running CIBERSORTx with default parameters for cell type deconvolution. It is questionable if new insights can be drawn from this analysis other than corroborating prior knowledge.

The goal of this analysis was to demonstrate that there is a path to systematically linking the non-spatial-but-cell-type-resolved data in TOME to the spatially-resolved-but-bulk data generated by methods like GEO-seq. At least to our knowledge, “whole embryo” scRNA-seq data has not previously been integrated with “whole embryo” spatially resolved bulk RNA-seq data in this manner, at least not in mouse. We acknowledge there are no new biological insights that can be drawn from this set of analyses, and it is largely corroborative. However, we believe that this may change as methods like GEO-seq are applied at higher spatial and temporal resolution.

• Various heuristic approaches were taken for identifying transcription factors and cis-regulatory motifs involved for cell type specification. The same pipeline was used to characterize zebrafish and frog embryogenesis and compare with mouse. While it was asserted that these identification and comparisons were systematic, these analyses were highly heuristic and ad-hoc. Although the recapitulation of prior knowledge might support the outcome of analysis cell type homology, further validation would be required to support the claim that the results of this analysis are at “systems” resolution. The ambiguous results of cell type homology, beyond the examples shown (Fig. 6), may stem from the depth of the transcriptome data and quality of the data related to the transcription factor profiles. This limitation may be discussed.

This is a very fair point. We agree that the manual filtering, while necessary, was non-systematic. We have removed the term “systematic” from this section, and further have added the following sentence to the discussion: “Of note, the set of apparent cell type homologs was noisy prior to manual filtering; and fully automating these assignments remains an outstanding goal.”

Knowledge gain

• While the TOME provides a global visualization of the molecular trajectory of cells in the embryo up to advanced stages of organogenesis (E12.5-E13.5), which may have bearing of the lineage trajectories during embryogenesis. Much of the outcome is corroborating prior knowledge of lineage relationship gleaned from embryological studies. A few examples of unexpected learning of the molecular drivers at various branching points of the cellular phylogeny and “new” cell types, which were presumed to “pseudo-ancestor” have been highlighted. The concept of “pseudo-ancestor” was not articulated and it is unclear if this is aligned with the notion of “lineage antecedent” or “quasi-progenitors”. While it may be a tall order, the merit of this study may be enhanced by experimental validation of the molecular attributes identified that potentially underpinning the transition of “pseudo-ancestors” to emergent cell types embedded in TOME of 413 cell states with 89 cell types (Fig. 4d) or revealed by multilineage priming modelling.

We apologize for failing to more explicitly define the term “pseudo-ancestor”. We now state: “Because these are inferred relationships based on transcriptional similarity, analogous to pseudotime, we use the terms pseudo-ancestor and pseudo-descendant to refer to cell states at the immediately preceding or immediately following cell state to which an edge has been drawn.” Although arguably aligned with the term “lineage antecedent”, we feel that term would be too strong, as it implies that we are explicitly

recording lineage relationships, as opposed to merely inferring them. In our experience, the term ‘quasi-progenitor’ is not widely used in the field (*e.g.* fewer than 200 Google hits to the search term). We therefore argue that relating our terminology to pseudotime, which is now a broadly understood concept, makes more sense.

To the other point, we recognize that many of the inferred relationships are corroborating existing literature, and even for our inference of key TFs, over 90% have some support in the literature (see response to query #4 from Reviewer 1). However, as the quality of published evidence around each of these relationships varies (and includes references from other model organisms or *in vitro* systems), we are moving forward with systematically perturbing sets of key TFs explicitly nominated by TOME in ESCs as well as mammalian gastruloid models. However, these are complex experiments being conducted by a different set of individuals and their addition would bloat an already long manuscript.

We would further argue that simply verifying and integrating our contemporary understanding of cell type relationships across early mammalian development, built up over several decades of experiments, via a data-driven, largely automated framework, represents an important step forward. As such, we feel the current work stands on its own as a resource for mammalian developmental biology.

• It is unclear how the data of TOME allow distinguishing between incomplete separation and convergence of cell type in ongoing differentiation. If the anticipated convergence between two cell states (e.g., visceral endoderm and gut (definitive) endoderm) was not supported by the weight analysis, what the relevant biological inference may be drawn from this analysis? Would the analysis of spatial rendered data of cellular heterogeneity or sub-set of data may identify a more credible relationship? How far one may go with this supervised approach like RNA velocity?

This is a challenge that we recognize, and may not fully be addressed until embryo-scale, high-resolution lineage tracing methods are fully implemented in the mouse system. In response to this comment as well as a related comment from Reviewer 2, we sought to more systematically ask how far we could push RNA velocity towards the same goal. For this, we recalculated edge weights between cell states at adjacent timepoints with an alternative heuristic that was based on RNA velocity. Specifically, we integrated cells from each pair of adjacent timepoints, and then applied RNA velocity analysis using *scVelo* (Bergen et al. 2020). The resulting transition probabilities between individual cells (stored in a *velocity_graph* matrix), were calculated using cosine correlation between the potential cell-to-cell transitions and the inferred velocity vector (ranging from 0 to 1). To calculate the transition probability from cell state *A* at the earlier timepoint to cell state *B* at the later timepoint, we summed the transition probabilities of all cells within *A* to all cells within *B*, followed by normalizing the total cell number of *B*. Finally, the edge weight from *A* to *B* was further calculated by normalizing their transition probability to the total transition probabilities which originated from *A*.

Comparing these heuristics, we found that out of 515 *k*-NN nominated edges with weights > 0.2, 392 had RNA velocity-based transition probabilities > 0.2 (76%) (see **Supplementary Fig. 14**, reproduced below). However, there were also 123 edges nominated by the *k*-NN strategy only, and 75 edges nominated by the RNA velocity strategy only (see panel c of **Supplementary Fig. 14**, reproduced below).

The result illustrates how the methods are complementary. Although we may assign greater confidence to edges nominated by both methods, edges supported by one method or the other may include both

true and false positives. To the reviewer's specific question and as an example of a likely true positive supported by the RNA velocity method only, the connection between embryonic visceral endoderm (E8.0) and gut (E8.25), fell short of the edge threshold by the *k*-NN strategy (0.14) but was strongly supported by the RNA velocity strategy (0.96).

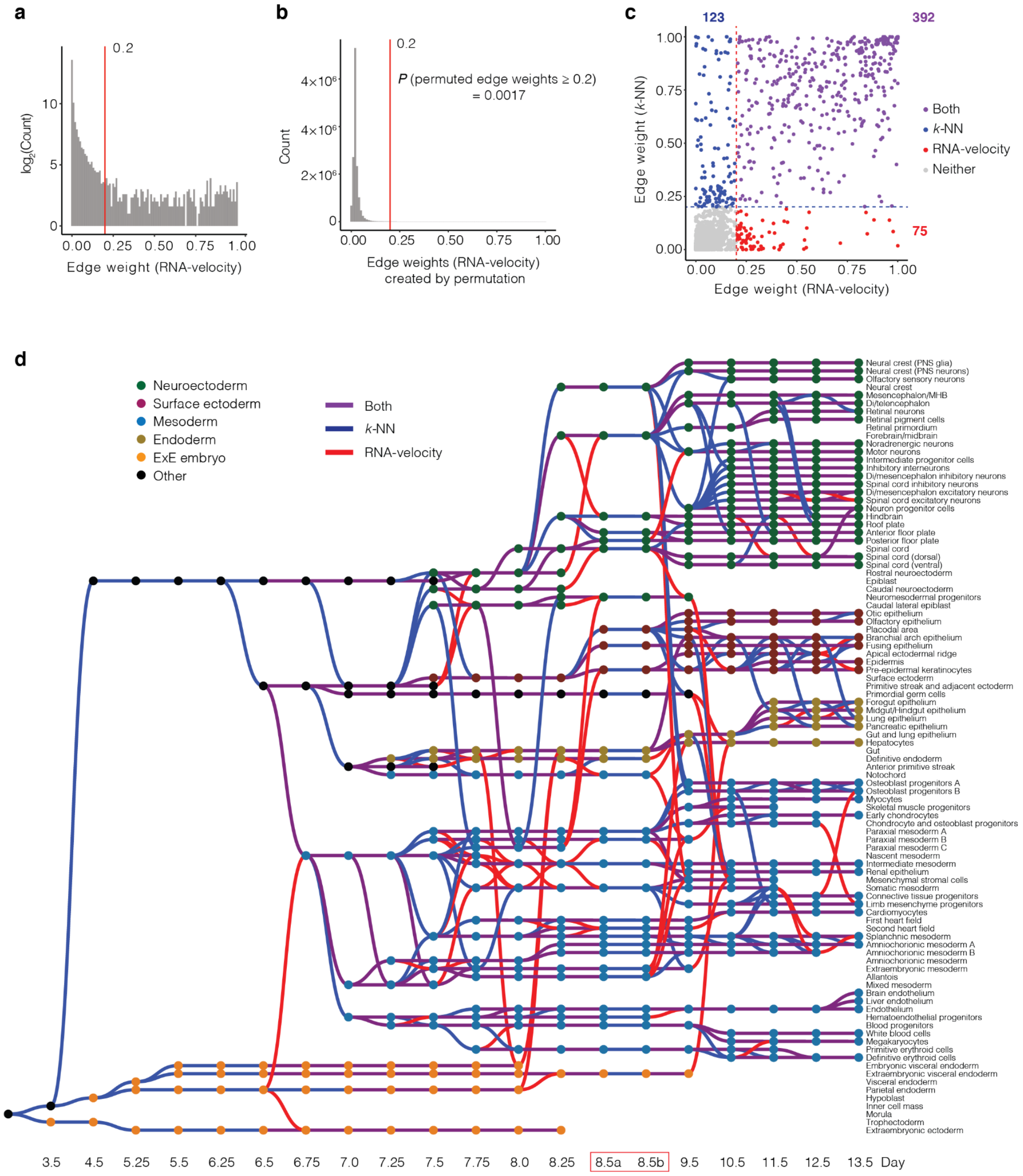

• The determination of cell states (Fig. 1) and the candidate TFs in the cellular trajectory (Fig. 4d) appeared to rely primarily on differential gene expression data. An alternative approach may be to anchor the analysis to the stably expressed and repressed genes across multiple steps in the trajectory. This information may be valuable for the elucidation of the transcription factors driving the cell trajectory as the activity of both the up- and down-regulated transcription factors (e.g., Supp Fig 5c and 7) may be instrumental for cell fate choice and identity. It is unclear if the expression of the TF's has any relationship with the enrichment and "diffused enrichment" of motif occupancy. Correlation of the stable changes in gene expression with variations in chromatin accessibility and configuration (e.g., PMID: 33278344), may yield mechanistically relevant information on the molecular activity that accompanies

lineage diversification. It may also be informative for elucidate the regulon activity along the molecular trajectory of the cell types.

Thank you for this interesting suggestion. We downloaded the repressive tendency score (RTS) file from (Shim et al. 2020). It includes 16,298 mouse genes with RTS that define the association between each gene and broad H3K27me3 domains. As described in (Shim et al. 2020), broad H3K27me3 domains occur mostly over important cell-type-specific regulatory genes (with high RTS); in contrast, genes with housekeeping or non-regulatory roles rarely host broad H3K27me3 domains (with low RTS). Indeed, we found that upregulated key TFs had relatively higher RTS compared to downregulated key TFs ($p = 5.38\times10^{-14}$, Wilcoxon rank-sum test) and non-key TFs ($p < 2.2\times10^{-16}$, Wilcoxon rank-sum test), while downregulated key TFs had similar RTS compared to non-key TFs ($p = 0.84$, Wilcoxon rank-sum test). This result supports the conclusion that upregulated key TFs nominated by TOME are associated with the broad H3K27me3 domains, consistent with expectation based on (Shim et al. 2020). This analysis is now presented as **Supplementary Fig. 20c**, reproduced below and now referenced in the discussion section.

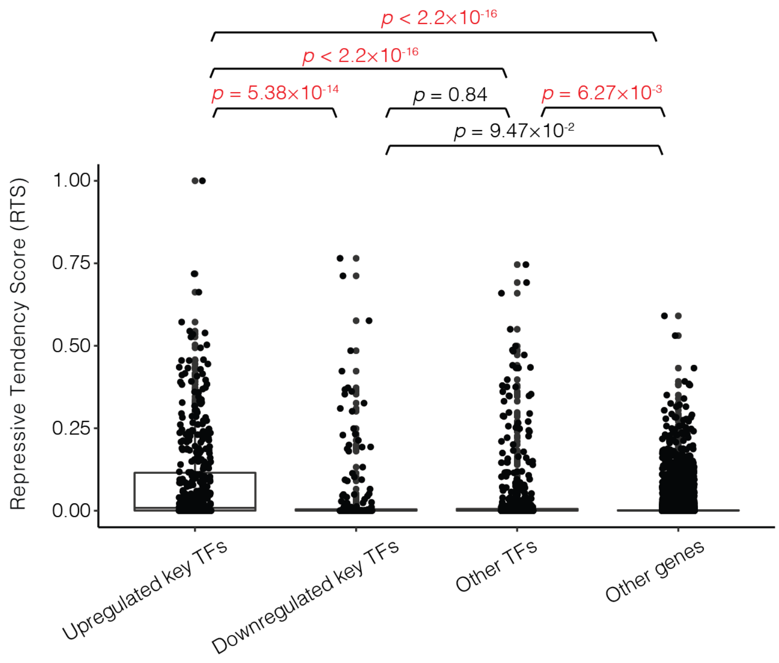

References

Bergen, Volker, Marius Lange, Stefan Peidli, F. Alexander Wolf, and Fabian J. Theis. 2020. "Generalizing RNA Velocity to Transient Cell States through Dynamical Modeling." *Nature Biotechnology* 38 (12): 1408–14.

Berger, Hanna, Andreas Wodarz, and Annette Borchers. 2017. "PTK7 Faces the Wnt in Development and Disease." *Frontiers in Cell and Developmental Biology* 5 (April): 31.

Briggs, James A., Caleb Weinreb, Daniel E. Wagner, Sean Megason, Leonid Peshkin, Marc W. Kirschner, and Allon M. Klein. 2018. "The Dynamics of Gene Expression in Vertebrate Embryogenesis at Single-Cell Resolution." *Science* 360 (6392). https://doi.org/10.1126/science.aar5780.

Cai, Chen-Leng, Xingqun Liang, Yunqing Shi, Po-Hsien Chu, Samuel L. Pfaff, Ju Chen, and Sylvia Evans. 2003. "Isl1 Identifies a Cardiac Progenitor Population That Proliferates prior to Differentiation and Contributes a Majority of Cells to the Heart." *Developmental Cell* 5 (6): 877–89.

Cao, Junyue, Malte Spielmann, Xiaojie Qiu, Xingfan Huang, Daniel M. Ibrahim, Andrew J. Hill, Fan Zhang, et al. 2019. "The Single-Cell Transcriptional Landscape of Mammalian Organogenesis." *Nature* 566 (7745): 496–502.

Dale, K., N. Sattar, J. Heemskerk, J. D. Clarke, M. Placzek, and J. Dodd. 1999. "Differential Patterning of Ventral Midline Cells by Axial Mesoderm Is Regulated by BMP7 and Chordin." *Development* 126 (2): 397–408.

Herrmann, Franziska, Karin Bundschu, Susanne J. Kühl, and Michael Kühl. 2011. "Tbx5 Overexpression Favors a First Heart Field Lineage in Murine Embryonic Stem Cells and in Xenopus Laevis Embryos." *Developmental Dynamics: An Official Publication of the American Association of Anatomists* 240 (12): 2634–45.

Hirata, Hiromi, Yasumasa Bessho, Hiroshi Kokubu, Yoshito Masamizu, Shuichi Yamada, Julian Lewis, and Ryoichiro Kageyama. 2004. "Instability of Hes7 Protein Is Crucial for the Somite Segmentation Clock." *Nature Genetics* 36 (7): 750–54.

Hu, Hui, Ya-Ru Miao, Long-Hao Jia, Qing-Yang Yu, Qiong Zhang, and An-Yuan Guo. 2019. "AnimalTFDB 3.0: A Comprehensive Resource for Annotation and Prediction of Animal Transcription Factors." *Nucleic Acids Research* 47 (D1): D33–38.

Javali, Alok, Aritra Misra, Karolis Leonavicius, Debalina Acharyya, Bhakti Vyas, and Ramkumar Sambasivan. 2017. "Co-Expression of Tbx6 and Sox2 Identifies a Novel Transient Neuromesoderm Progenitor Cell State." *Development* 144 (24): 4522–29.

Lau, Wim B. M. de, Berend Snel, and Hans C. Clevers. 2012. "The R-Spondin Protein Family." *Genome Biology* 13 (3): 242.

Martin, Beth K., Chengxiang Qiu, Eva Nichols, Melissa Phung, Rula Green-Gladden, Sanjay Srivatsan, Ronnie Blecher-Gonen, et al. 2021. "An Optimized Protocol for Single Cell Transcriptional Profiling by Combinatorial Indexing." *arXiv [q-bio.GN]*. arXiv. http://arxiv.org/abs/2110.15400.

Maves, Lisa, William Jackman, and Charles B. Kimmel. 2002. "FGF3 and FGF8 Mediate a Rhombomere 4 Signaling Activity in the Zebrafish Hindbrain." *Development* 129 (16): 3825–37.

Mohammed, Hisham, Irene Hernando-Herraez, Aurora Savino, Antonio Scialdone, Iain Macaulay, Carla Mulas, Tamir Chandra, et al. 2017. "Single-Cell Landscape of Transcriptional Heterogeneity and Cell Fate Decisions during Mouse Early Gastrulation." *Cell Reports* 20 (5): 1215–28.

Pijuan-Sala, Blanca, Jonathan A. Griffiths, Carolina Guibentif, Tom W. Hiscock, Wajid Jawaid, Fernando J. Calero-Nieto, Carla Mulas, et al. 2019. "A Single-Cell Molecular Map of Mouse Gastrulation and Early Organogenesis." *Nature* 566 (7745): 490–95.

Placzek, Marysia, and James Briscoe. 2005. "The Floor Plate: Multiple Cells, Multiple Signals." *Nature Reviews. Neuroscience* 6 (3): 230–40.

Rana, M. Sameer, Magali Théveniau-Ruissy, Christopher De Bono, Karim Mesbah, Alexandre Francou, Mayyasa Rammah, Jorge N. Domínguez, et al. 2014. "Tbx1 Coordinates Addition of Posterior Second Heart Field Progenitor Cells to the Arterial and Venous Poles of the Heart." *Circulation Research* 115 (9): 790–99.

Sakurai, Takeshi. 2012. "The Role of NrCAM in Neural Development and Disorders--beyond a Simple Glue in the Brain." *Molecular and Cellular Neurosciences* 49 (3): 351–63.

Sambasivan, Ramkumar, and Benjamin Steventon. 2020. “Neuromesodermal Progenitors: A Basis for Robust Axial Patterning in Development and Evolution.” *Frontiers in Cell and Developmental Biology* 8: 607516.
Shim, Woo Jun, Enakshi Sinniah, Jun Xu, Burcu Vitrinel, Michael Alexanian, Gaia Andreoletti, Sophie Shen, et al. 2020. “Conserved Epigenetic Regulatory Logic Infers Genes Governing Cell Identity.” *Cell Systems* 11 (6): 625–39.e13.
Shintani, T., E. Watanabe, N. Maeda, and M. Noda. 1998. “Neurons as Well as Astrocytes Express Proteoglycan-Type Protein Tyrosine Phosphatase zeta/RPTPbeta: Analysis of Mice in Which the PTPzeta/RPTPbeta Gene Was Replaced with the LacZ Gene.” *Neuroscience Letters* 247 (2-3): 135–38.
Später, Daniela, Monika K. Abramczuk, Kristina Buac, Lior Zangi, Maxine W. Stachel, Jonathan Clarke, Makoto Sahara, Andreas Ludwig, and Kenneth R. Chien. 2013. “A HCN4+ Cardiomyogenic Progenitor Derived from the First Heart Field and Human Pluripotent Stem Cells.” *Nature Cell Biology* 15 (9): 1098–1106.
Studer, M., A. Gavalas, H. Marshall, L. Ariza-McNaughton, F. M. Rijli, P. Chambon, and R. Krumlauf. 1998. “Genetic Interactions between Hoxa1 and Hoxb1 Reveal New Roles in Regulation of Early Hindbrain Patterning.” *Development* 125 (6): 1025–36.
Tang, Changyong, Min Wang, Peijian Wang, Lei Wang, Qingfeng Wu, and Weixiang Guo. 2019. “Neural Stem Cells Behave as a Functional Niche for the Maturation of Newborn Neurons through the Secretion of PTN.” *Neuron* 101 (1): 32–44.e6.
Wilson, V., L. Manson, W. C. Skarnes, and R. S. Beddington. 1995. “The T Gene Is Necessary for Normal Mesodermal Morphogenetic Cell Movements during Gastrulation.” *Development* 121 (3): 877–86.
Yates, Andrew D., Premanand Achuthan, Wasiu Akanni, James Allen, Jamie Allen, Jorge Alvarez-Jarreta, M. Ridwan Amode, et al. 2020. “Ensembl 2020.” *Nucleic Acids Research* 48 (D1): D682–88.

natureresearch

Decision Letter, first revision:

Our ref: NG-A57219R

18th Nov 2021

Dear Jay,

Thank you for submitting your revised manuscript entitled "Systematic reconstruction of the cellular trajectories of mouse embryogenesis" (NG-A57219R). It has now been seen by the three original referees and their comments are below. The reviewers find that the paper has improved in revision, and therefore we'll be happy in principle to publish it in Nature Genetics, pending minor revisions to satisfy reviewer #3's final request and to comply with our editorial and formatting guidelines.

Since the current version of your manuscript is in a PDF format, please email us (Cc: genetics@us.nature.com) a copy of the file in an editable format (Microsoft Word)- we can not proceed with PDFs at this stage.

We will then be performing detailed checks on your paper and will send you a checklist detailing our editorial and formatting requirements soon afterwards. Please do not upload the final materials and make any revisions until you receive this additional information from us.

Thank you again for your interest in Nature Genetics. Please do not hesitate to contact me if you have any questions.

Congratulations!

Sincerely,

Tiago

Tiago Faial, PhD
Senior Editor
Nature Genetics
https://orcid.org/0000-0003-0864-1200

Reviewer #1 (Remarks to the Author):

The authors have addressed all of my concerns. The revisions are excellent. I fully agree with the authors that adding the new E8.5 dataset considerably strengthens their work (which was already pretty strong, in my view).

Reviewer #2 (Remarks to the Author):

The authors have addressed all of my comments admirably. The extensive new analyses, clarifications, experimental data, and online data/code resources have strengthened the study considerably. Congratulations to the authors on a thoughtful study and important resource. The manuscript in its present and substantially improved form should be suitable for publication.

Reviewer #3 (Remarks to the Author):

In the revised manuscript, the authors have adequately addressed the attributes and limitations of TOME as a scaffolding tool to integrate single-cell data across time to construct molecular trajectory and putative TF drivers of the acquisition of propensity of cell lineage development.
Additional single-cell data of a time/somite-stage series of early somite embryos were incorporate in the analysis to reduce the gaps in the information of transitional cell states at the time of expeditious cell differentiation and morphogenesis.
It is nevertheless unclear how far advanced in embryonic development should more frequent samplings (than a 24-hour interval) would be needed to mitigate the impact of "rapid" changes in transcriptome for connecting "concordant" pseudo-ancestors and pseudo-descendants in the molecular developmental trajectory. A note on this point may be added to the Discussion.

Author Rebuttal, first revision:

To address the final comment from Reviewer 3, the discussion now reads (added clause bolded): "Although it remains unclear exactly how frequent sampling needs to be in order to fully mitigate confounding by rapid transitions in transcriptional states, near-term goals that we are pursuing include consistent sc-RNA-seq sampling of mouse development at least every 6 hours, from fertilization to birth."

naturereseach

Final Decision Letter:

In reply please quote: NG-A57219R1 Shendure

21st Jan 2022

Dear Jay,

I am delighted to say that your manuscript, entitled "Systematic reconstruction of cellular trajectories across mouse embryogenesis", has been accepted for publication in an upcoming issue of Nature Genetics.

Over the next few weeks, your paper will be copy-edited to ensure that it conforms to Nature Genetics style. Once your paper is typeset, you will receive an email with a link to choose the appropriate publishing options for your paper and our Author Services team will be in touch regarding any additional information that may be required.

After the grant of rights is completed, you will receive a link to your electronic proof via email with a request to make any corrections within 48 hours. If, when you receive your proof, you cannot meet this deadline, please inform us at rjsproduction@springernature.com immediately.

You will not receive your proofs until the publishing agreement has been received through our system.

Due to the importance of these deadlines, we ask that you please let us know now whether you will be difficult to contact over the next month. If this is the case, we ask you provide us with the contact information (email, phone and fax) of someone who will be able to check the proofs on your behalf, and who will be available to address any last-minute problems.

Your paper will be published online after we receive your corrections and will appear in print in the next available issue. You can find out your date of online publication by contacting the Nature Press Office (press@nature.com) after sending your e-proof corrections. Now is the time to inform your Public Relations or Press Office about your paper, as they might be interested in promoting its publication. This will allow them time to prepare an accurate and satisfactory press release. Include your manuscript tracking number (NG-A57219R1) and the name of the journal, which they will need when they contact our Press Office.

Before your paper is published online, we shall be distributing a press release to news organizations worldwide, which may very well include details of your work. We are happy for your institution or funding agency to prepare its own press release, but it must mention the embargo date and Nature Genetics. Our Press Office may contact you closer to the time of publication, but if you or your Press Office have any inquiries in the meantime, please contact press@nature.com.

Acceptance is conditional on the data in the manuscript not being published elsewhere, or announced

in the print or electronic media, until the embargo/publication date. These restrictions are not intended to deter you from presenting your data at academic meetings and conferences, but any enquiries from the media about papers not yet scheduled for publication should be referred to us.

Please note that <i>Nature Genetics</i> is a Transformative Journal (TJ). Authors may publish their research with us through the traditional subscription access route or make their paper immediately open access through payment of an article-processing charge (APC). Authors will not be required to make a final decision about access to their article until it has been accepted. Find out more about Transformative Journals

<B>Authors may need to take specific actions to achieve compliance with funder and institutional open access mandates. For submissions from January 2021, if your research is supported by a funder that requires immediate open access (e.g. according to Plan S principles) then you should select the gold OA route, and we will direct you to the compliant route where possible. For authors selecting the subscription publication route our standard licensing terms will need to be accepted, including our self-archiving policies. Those standard licensing terms will supersede any other terms that the author or any third party may assert apply to any version of the manuscript.

Please note that Nature Research offers an immediate open access option only for papers that were first submitted after 1 January, 2021.

If you have any questions about our publishing options, costs, Open Access requirements, or our legal forms, please contact ASJournals@springernature.com

If you have posted a preprint on any preprint server, please ensure that the preprint details are updated with a publication reference, including the DOI and a URL to the published version of the article on the journal website.

To assist our authors in disseminating their research to the broader community, our SharedIt initiative provides you with a unique shareable link that will allow anyone (with or without a subscription) to read the published article. Recipients of the link with a subscription will also be able to download and print the PDF.

As soon as your article is published, you will receive an automated email with your shareable link.

You can now use a single sign-on for all your accounts, view the status of all your manuscript submissions and reviews, access usage statistics for your published articles and download a record of your refereeing activity for the Nature journals.

An online order form for reprints of your paper is available at https://www.nature.com/reprints/author-reprints.html. Please let your coauthors and your institutions' public affairs office know that they are also welcome to order reprints by this

method.

Sincerely,

Tiago

Tiago Faial, PhD
Senior Editor
Nature Genetics
https://orcid.org/0000-0003-0864-1200

Click here if you would like to recommend Nature Genetics to your librarian
http://www.nature.com/subscriptions/recommend.html#forms

** Visit the Springer Nature Editorial and Publishing website at www.springernature.com/editorial-and-publishing-jobs for more information about our career opportunities. If you have any questions please click here.**
